# Are interventions to promote healthy eating equally effective for all? Systematic review of socioeconomic inequalities in impact

**DOI:** 10.1186/s12889-015-1781-7

**Published:** 2015-05-02

**Authors:** Rory McGill, Elspeth Anwar, Lois Orton, Helen Bromley, Ffion Lloyd-Williams, Martin O’Flaherty, David Taylor-Robinson, Maria Guzman-Castillo, Duncan Gillespie, Patricia Moreira, Kirk Allen, Lirije Hyseni, Nicola Calder, Mark Petticrew, Martin White, Margaret Whitehead, Simon Capewell

**Affiliations:** Department of Public Health and Policy, University of Liverpool, Liverpool, UK; Public and Environmental Health Research Unit, London School of Hygiene and Tropical Medicine, Liverpool, UK; UKCRC Centre for Diet and Activity Research (CEDAR), MRC Epidemiology Unit, University of Cambridge School of Clinical Medicine, Institute of Metabolic Science, Cambridge, UK; Institute of Health and Society, Newcastle University, Newcastle upon Tyne, UK

**Keywords:** Noncommunicable diseases, Socioeconomic inequalities, Healthy eating, Intervention

## Abstract

**Background:**

Interventions to promote healthy eating make a potentially powerful contribution to the primary prevention of non communicable diseases. It is not known whether healthy eating interventions are equally effective among all sections of the population, nor whether they narrow or widen the health gap between rich and poor.

We undertook a systematic review of interventions to promote healthy eating to identify whether impacts differ by socioeconomic position (SEP).

**Methods:**

We searched five bibliographic databases using a pre-piloted search strategy. Retrieved articles were screened independently by two reviewers. Healthier diets were defined as the reduced intake of salt, sugar, trans-fats, saturated fat, total fat, or total calories, or increased consumption of fruit, vegetables and wholegrain. Studies were only included if quantitative results were presented by a measure of SEP.

Extracted data were categorised with a modified version of the *“4Ps”* marketing mix, expanded to *6 “Ps”:* “Price, Place, Product, Prescriptive, Promotion, and Person”.

**Results:**

Our search identified 31,887 articles. Following screening, 36 studies were included: 18 “Price” interventions, 6 “Place” interventions, 1 “Product” intervention, zero “Prescriptive” interventions, 4 “Promotion” interventions, and 18 “Person” interventions.

“Price” interventions were most effective in groups with lower SEP, and may therefore appear likely to reduce inequalities. All interventions that combined taxes and subsidies consistently decreased inequalities. Conversely, interventions categorised as “Person” had a greater impact with increasing SEP, and may therefore appear likely to reduce inequalities. All four dietary counselling interventions appear likely to widen inequalities.

We did not find any “Prescriptive” interventions and only one “Product” intervention that presented differential results and had no impact by SEP. More “Place” interventions were identified and none of these interventions were judged as likely to widen inequalities.

**Conclusions:**

Interventions categorised by a “6 Ps” framework show differential effects on healthy eating outcomes by SEP. “Upstream” interventions categorised as “Price” appeared to decrease inequalities, and “downstream” “Person” interventions, especially dietary counselling seemed to increase inequalities.

However the vast majority of studies identified did not explore differential effects by SEP. Interventions aimed at improving population health should be routinely evaluated for differential socioeconomic impact.

**Electronic supplementary material:**

The online version of this article (doi:10.1186/s12889-015-1781-7) contains supplementary material, which is available to authorized users.

## Background

Non communicable diseases (NCD’s e.g. cardiovascular disease (CVD), chronic obstructive pulmonary disease, diabetes, cancer, etc.) remain the major cause of disease, disability and death, accounting for over 63% of deaths worldwide in 2012 [1]. A substantial amount of the NCD burden is attributable to four behavioural risk factors (notably poor diet, also smoking, alcohol and physical inactivity). Poor nutrition causes a greater population burden of morbidity and mortality from NCDs than tobacco, alcohol and physical activity combined [2]. Furthermore, the prevalence of NCD risk factors and hence burden of NCDs are not equally distributed throughout the population [3]. There is evidence for an inverse relationship between socioeconomic position (SEP) and most risk factors, with NCD risk factors often being higher in more disadvantaged groups (low SEP) [3].

Thus, eating a healthy diet demonstrates a social gradient with diet among people in lower SEPs being poorer in quality when compared to more advantaged groups. The World Health Organisation (WHO) define a healthy diet as achieving energy balance, limiting energy intake from total fats, free sugars and salt and increasing consumption of fruits and vegetables, legumes, whole grains and nuts [4] Lower SEP is associated with a higher intake of energy dense, nutrient poor foods (which are high in saturated fat and sugar), and with lower intake of fruit, vegetables and wholegrains [5].

Socioeconomic inequalities in diet are influenced by factors including cost, access and knowledge. A diet relatively high in energy is generally less expensive than a diet consisting of less energy dense products, such as vegetables [6]. Food selection is not only a behavioural choice, but also an economic one [7]. Access to healthy foods can also be inequitable. This can be a lack of healthy food options provided in shops within disadvantaged areas [8] which has been described in the US in terms of “food deserts”, however evidence for these have not been found within other settings e.g. UK [9]. Significant differences in nutritional knowledge have been shown between differing socioeconomic groups, with knowledge declining with lower socioeconomic status [10]. In children, lower SEP is associated with a subsequent increased risk of adult cardiovascular morbidity and mortality, partly reflecting lower exposure to healthy foods [11]. This can then reinforce adult food preferences for less healthy foods [12].

There has been considerable effort to develop population-wide dietary interventions. These primary prevention programmes are aimed at asymptomatic individuals in the normal population, before any negative health event has occurred [13]. Interventions at this stage aim to modify NCD risk factors through the promotion of healthier diets. Potentially powerful interventions are available which target the components outlined above - cost, access and knowledge. Furthermore, such population interventions, by their very nature, should theoretically benefit everyone in the population, including those with a history of NCD such as CVD.

However, there is a lack of evidence concerning the health equity impact of dietary interventions to promote health. This has led to an increase in systematic reviews assessing health equity effects [14,15]. Preventive interventions may not benefit all sub groups of the population equally [16,17]. This has been termed “intervention generated inequalities” or “IGIs” [18].

White et al. have described the points in the implementation of an intervention which may impact upon differential effectiveness by SEP [18]. These include intervention efficacy, service provision or access, uptake, and compliance [15]. Compliance may be higher among more advantaged groups because of better access to resources such as time, finance, and coping skills. “Downstream” interventions (which rely solely on individuals making and sustaining behaviour change) may therefore be more likely to be taken up by those who are of higher SEP and are more likely to widen the health gap between rich and poor. Conversely, those of lower SEP tend to be harder to reach, and find it harder to change behaviour due to a lack of access to the resources previously outlined [19]. “Upstream” interventions remove this reliance on resource availability. Due to a higher risk burden, those of lower SEP are likely to gain extra benefit if a risk factor is uniformly reduced across the entire population. Therefore being more likely to reduce inequalities [16,20].

Thomas and colleagues demonstrated differential impact of tobacco control policy interventions. They showed that population level tobacco control interventions, such as increasing the price of tobacco products had a greater potential to benefit more disadvantaged groups and thereby reduce health inequalities [17]. With deprived groups already having a higher NCD burden (in 2008 worldwide age standardised mortality rates from NCDs were almost twice as high for lower income groups when compared to higher income groups [1]), there is an urgent need to further explore this important issue relating to the major NCD risk factor, diet [2,21].

Oldroyd and colleagues [22] previously examined the differential effects of healthy eating interventions by relative social disadvantage. In their small number of included studies they found limited evidence of greater impact in less disadvantaged groups [22]. This may be due to their chosen time frame (1990–2007) and limited databases searched (MEDLINE and CINAHL).

Our aim was to update and expand upon Oldroyd and colleagues review [22]. In order to identify interventions which may reduce inequalities in healthy eating, we undertook a systematic review of interventions (and modelling studies) to promote healthy eating in general populations, to determine whether impacts differ by SEP.

## Methods

### Study design

We conducted a systematic review with a combination of graphical and narrative synthesis of published literature. We followed best practice guidance as detailed by the PRISMA-Equity 2012 Extension for systematic reviews with a focus on health equity. This tool has been described as a method to improve both the reporting and conduct of equity focused systematic reviews [23] (provided in the additional information – Additional file 1).

### Search strategy

In order to identify all relevant studies, a pre-piloted search strategy was used to search five bibliographic databases (MEDLINE, Psycinfo, SCI, SSCI and SCOPUS). An example of the search strategy used is provided in the additional information (Additional file 2). In addition, we screened titles from the reference sections of systematic reviews in the Campbell library, CENTRAL, DARE and EPPI. Colleagues and experts from key organisations working in public health policy were also contacted for any additional data sources. The reference lists of all included studies (including relevant systematic reviews that were identified) were scrutinised for other potentially eligible studies.

### Study selection and inclusion criteria

We included studies of any design that assessed the effects of interventions to promote healthy eating (reduced intake of salt, sugar, trans-fats, saturated fat, total fat, or total calories, or increased consumption of fruit, vegetables and wholegrain) targeted at healthy populations that reported quantitative outcomes by a measure of SEP. Only studies published since 1980 in the English language were considered. Upon fulfilling these criteria, studies were assessed utilising a PICOS (Participants, Interventions, Comparators, Outcomes and Study design) [23]. This is summarised in Table 1.

**Table 1:** PICOS approach to study eligibility*

**Include**	**Exclude**
**Participants**	
Healthy populations (any age or gender), from any country	Studies including participants that were not representative of the population were excluded (e.g. sub categories such as obese participants in weight loss trials, participants with diabetes, pregnant women).
**Interventions**	
Studies evaluating the effects of intervention to promote healthy eating that were implemented experimentally; or due to local or national policies. These could include a range of actions to improve healthy eating (in terms of the dietary factors of salt, sugar, trans fats, saturated fat, total fat, fruit and vegetables and calories).	Interventions with no change in healthy eating outcomes quantitatively stratified by SEP.
	Actions initiated by industry.
**Comparators**	
Studies were only included in the review provided that the authors made a quantitative comparison of differential effects of policy interventions to improve healthy eating by at least one measure of SEP.	Studies which did not report the effects of actions to improve healthy eating by SEP
**Outcomes**	
The primary outcome of interest was dietary intake. Secondary outcomes included: changes in clinical/physiological indicators related to NCD, behaviours associated with a healthy diet e.g. change in BMI.	Process evaluations reporting on implementation of interventions/policies without any outcome data; data only on costs, or feasibility or acceptability without an assessment of intake; reviews/studies of under-nutrition.
	Studies with no mention of SEP.
**Study design**	
We included studies of any design, including RCTs, cohort studies and modelling studies. We explicitly included modelling studies to better capture analysis of fiscal measures such as taxes, subsidies, or economic incentives	Opinion articles; purely qualitative evaluations with no quantitative assessment; data/statistics from monitoring and surveillance not directly linked to a policy intervention

*PICOS = Participants, Interventions, Comparators, Outcomes and Study design.

One reviewer (RMcG) screened titles, removed duplicates and selected potentially relevant abstracts. Then two reviewers (RMcG & EA) independently examined all the abstracts for eligibility. All articles deemed potentially eligible were retrieved in full text. The full text was also retrieved for any abstracts where a decision could not be made based on the information given. Full text articles were then screened independently by the two reviewers (RMcG & EA). Disagreements on eligibility decisions were resolved by consensus or by recourse to a senior member of the review team (SC).

### Data extraction and management

Data from all included studies were extracted by one reviewer using pre-designed and piloted forms. The extracted data was then checked independently by a second reviewer to ensure all the correct information was recorded. Extracted data included: study design, aims, methodological quality, setting, participants, and outcomes related to the review objectives. Extracted data were compared for accuracy and completeness. Where more information was required from an identified article, the authors were contacted where possible.

The measurement of SEP within the intervention was carefully noted and included: education level, level of household income, occupational status and ethnicity, as determined by the authors [24,25]. Ethnicity was only included as a measure of SEP if the authors explicitly stated this was their SEP measurement proxy within the text. If not, we assumed that these were measures of cultural differences rather than socioeconomic inequalities and these were excluded from the main analysis [26]. Interventions targeting only deprived groups were not included as these did not include a comparison of the effects of an intervention with higher SEP. All data extraction tables are included in the additional information (Additional file 3).

### Assessment of methodological quality of included studies

The methodological quality of each included study was assessed independently by two reviewers using the criteria for the Community Guide of the US Task Force on Community Preventive Services and a six-item checklist of quality of execution adapted from the criteria developed for the Effective Public Health Practice Project [27,28]. Several of the included studies were modelling studies. Since these studies could not be assessed using the same quality assessment tool as the empirical studies, two modelling experts assessed the quality of these independently. Disagreements in methodological quality assessment for all the included studies were resolved by consensus or by recourse to a senior member of the review team.

### Data synthesis

We examined the evidence about the differential effects of interventions in terms of their underlying theories of change [29]. Different frameworks have been proposed to categorise healthy eating interventions [30]. However no one framework has been used consistently. The “4 Ps” framework is a well-established framework used within the marketing field and translates well to a policy context [31]. This framework includes interventions examining “Price”, “Place”, “Product” and “Promotion”. We have adapted and strengthened this framework in order to categorise policy interventions relating to healthy eating by their mechanisms of underlying change.

The six intervention categories used in the analysis are thus:

- **Price** – fiscal measures such as taxes, subsidies, or economic incentives
- **Place** – environmental measures in specific settings such as schools, work places (e.g. vending machines) or planning (e.g. location of supermarkets and fast food outlets) or community-based health education
- **Product** – modification of food products to make them healthier/less harmful e.g. reformulation, additives, or elimination of a specific nutrient
- **Prescriptive** – restrictions on advertising/marketing through controls or bans, labelling, recommendations or guidelines
- **Promotion** – mass media public information campaigns
- **Person** –Individual-based information and education (e.g. cooking lessons, tailored nutritional education/counselling, or nutrition education in the school curriculum).

### Socioeconomic inequalities in impact

For each of the included interventions, if the outcome was split by more than one socioeconomic proxy measure, we took the quantitative effect on inequalities from the stratified results that best represented SEP [24,25].

When calculating the effect on inequalities, we examined the primary outcome of interest for each intervention as identified by the study author. If a change in dietary intake was given this was the primary measure that was used. If not, some other secondary outcomes were acceptable (see Table 1). We compared the lowest group with the highest group in the SEP classification, and used the measures of significance reported by the authors (e.g. p values, confidence intervals, standard deviations, standard error of measurement) to assess the significance of any differential effects of interventions by SEP. When the results were stratified by age, gender or intervention site, the results referring to the largest subsample were used. Where information was given at different time points, the longest follow up period was examined.

The effect on inequalities was classified as follows:

- Intervention likely to reduce inequalities: the intervention preferentially improved healthy eating outcomes in people of lower SEP
- Intervention likely to widen inequalities: the intervention preferentially improved healthy eating outcomes in people of higher SEP
- Intervention which had no preferential impact by SEP (this also includes interventions where there was an overall benefit but where there was no effect on healthy eating outcomes for any SEP sub-group).

**Figure 1:**
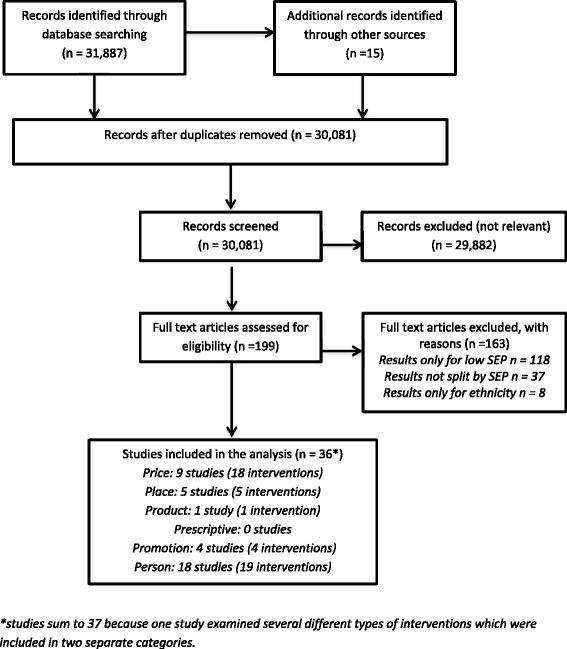
Harvest Plot summarising the effects of healthy eating interventions on inequalities*. *Each matrix within the Harvest plot ‘supermatrix’ illustrates our findings for each “P”. Each matrix consists of three columns indicating whether inequalities were reduced, widened or showed no gradient. Each bar represents one intervention. The height of the bar indicates the quality score of the study graded out of 6 [28]. Grey bars indicate interventions with no significance values given concerning the difference in effect of the intervention on SEP. Modelling studies are indicated by patterned bars

We aspired to undertake a meta-analysis of the results. However the studies identified were heterogeneous, addressing different research questions, with diverse theoretical underpinnings study designs and study outcomes. Given the considerable heterogeneity of the studies, undertaking a meta-analysis was not deemed appropriate. The results were therefore synthesised using a combination of graphical and narrative methods, including the use of the Harvest plot, which is a useful graphical method for synthesising and displaying evidence about the differential effects of population-level interventions [32]. Within the Harvest Plot, each intervention was represented as a single bar in one of three categories: those that were more effective in more disadvantaged groups (reduce), had the same effect in all groups (no preferential impact by SEP), or were less effective in disadvantaged groups (widen) (Figure 1).

### Sensitivity analyses

We conducted a sensitivity analysis to determine if the key results would change if we had been more or less selective in our study screening process.

First, we included only the studies which gave indicators of statistical significance concerning the quantitative data split by SEP. Secondly, we also included those studies which split their findings quantitatively by ethnicity alone (with no mention of SEP), as this represents a crude proxy measure of SEP [33] (see additional information - Additional file 4).

## Results

**Figure 2:**
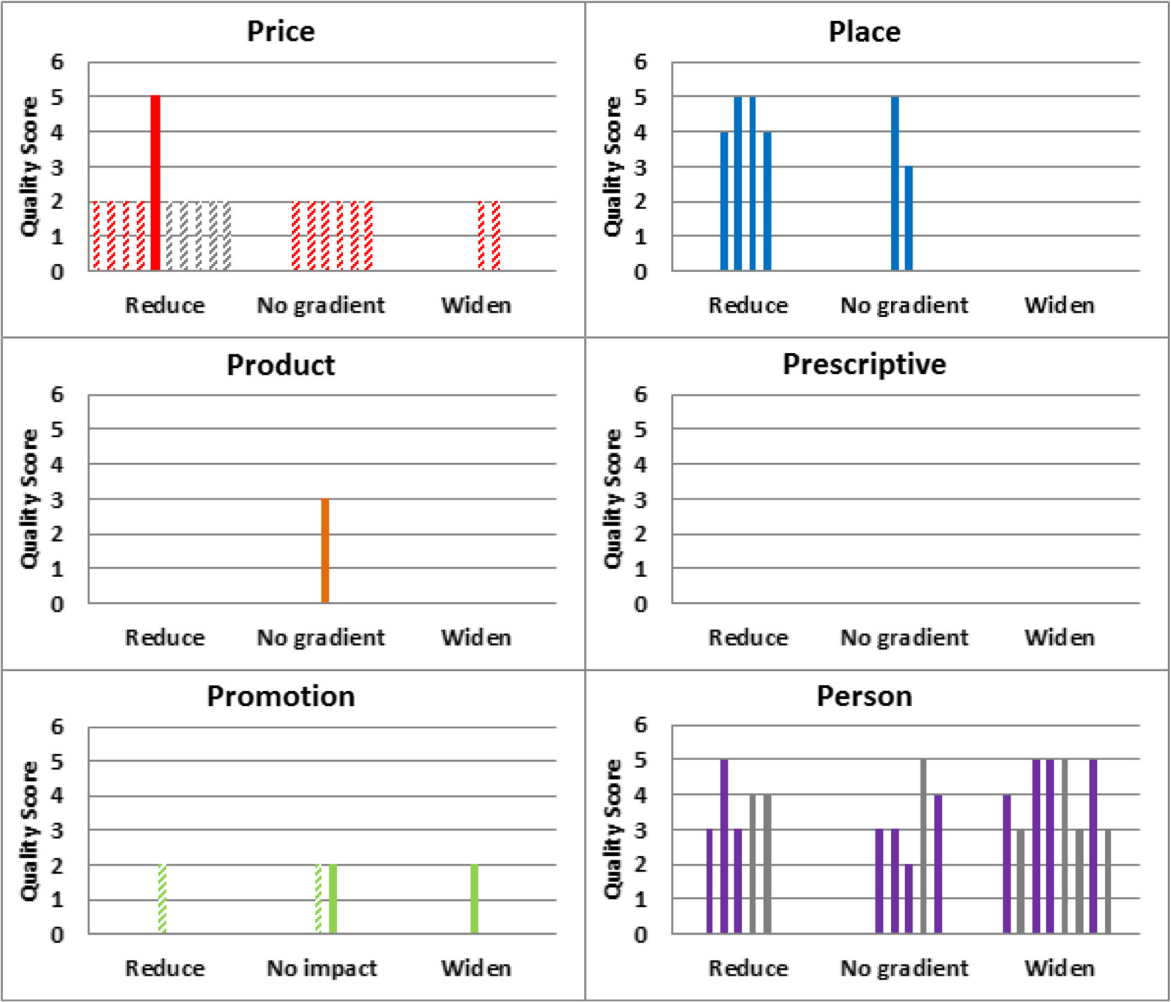
Flow chart showing the progress of the review. *studies sum to 37 because one study examined several different types of interventions which were included in two separate categories.

We identified 31,887 articles in our search. Following abstract and full text screening, 36 studies met the inclusion criteria (Figure 2). These included quantitative results presented by a measure of SEP for 47 interventions. A summary of all included studies is listed in the additional information (Additional file 5). Data extraction tables for all included studies and studies included in the sensitivity analysis are provided in the additional information (Additional file 3).

### Impact on socioeconomic inequalities by “P” category

The impact of interventions categorised by “P” is displayed in the Harvest plot in Figure 1 (adapted from Thomas et al. [17]). The Harvest plot shows each intervention illustrated as an individual bar. The height of the bar depicts the quality of the study. Modelling studies were distinguished by using patterned bars.

The studies are then grouped by outcome regarding socioeconomic differential effects (reduced, no preferential impact by SEP and widened). Interventions in the “Price” category appeared most likely to reduce inequalities while “Person” interventions were the most likely to widen inequalities (Figure 1).

### Price interventions (taxes, subsidies, or economic incentives)

Eighteen “Price” interventions were identified. These are summarised in Table 2. The majority were conducted in Europe [34-39], with five in North America [40,41] and one in Australia [42]. Of these, nine were taxes on high energy density foods [34,36,37,41,42], three were subsidies on fruit and vegetables [35,40] and six were combinations of taxes and subsidies [37-39]. Eight studies used modelling methodologies [34,35,37-42].

**Table 2:** **Summary of “Price” interventions**

**Author**	**Study**	**Setting**	**Intervention**	**Quality** ^**∆**^	**Outcome measured**	**SEP measurement**	**Effect on SEP inequalities** ^**†**^
**Allais [** 34 **]**	Modelling study	France	10% Tax on high energy density food:	2	Change in fat consumed (%)	Household income	**↓**
**Cash [** 40 **]**	Modelling study	USA	1% Subsidy on fruit and vegetables	2	CHD incidence	Household income	↑*
**Dallongeville** **[** 35 **]**	Modelling study	France	5.5% to 2.1% Subsidy on fruit and vegetables	2	Change in mean fruit and vegetable consumption (g/d)	Household income	↔*
			Food stamp program for fruit and vegetables				↓*
**Finkelstein [** 41]	Modelling study	Canada	20% Tax on high energy density food	2	Mean change in energy intake from all beverages	Household income	↔*
			40% tax on carbonated sugar sweetened beverages				↔*
			20% tax on all sugar sweetened beverages				↔*
			40% tax on all sugar sweetened beverages				↔*
**Nederkoorn** **[** 36 **]**	RCT	Holland	50% Tax on high energy density food	5	% change in calories purchased in lean individuals	Food budget	↓*
**Nnoaham [** 37 **]**	Modelling study	UK	17.5% tax on high energy density foods	2	% change in calorie intake	Household income	↔*
			17.5% tax on food classified as ‘less healthy’ by nutrient profiling				↓*
			Combined the taxation on ‘less healthy’ foods with a 17.5% subsidy on fruit and vegetables				↓*
			As above with a 32.5% subsidy on fruit and vegetables				↓*
**Sharma** **[** 42 **]**	Modelling study	Australia	20% tax on sugar sweetened beverages	2	Mean net change in body weight in kg	Household income	↑*
**Smed** **[** 38 **]**	Modelling study	Denmark	5% tax on fatty meat and dairy products with subsidies on fruit and vegetables, potatoes and grain products	2	Change in nutrient demand of saturated fat (%)	Social class	**↓**
			7.89 DKK/kg tax on saturated fats with subsidies on fibre				**↓**
			7.89 DKK/kg tax on saturated fats with subsidies on fibre with an additional 10.3 DKK/kg tax on sugar				**↓**
**Tiffin [** 39 **]**	Modelling study	UK	1% Tax on fatty food for every % saturated fat content with a matching subsidy on fruit and vegetables	2	% change in energy intake	Occupation	**↓**

^∆^Quality of empirical studies were assessed using a validated tool [27]. Studies were scored against six criteria and this number was summed to give an overall quality score (maximum of six). The modelling studies were assessed for quality by two independent experts and their scores were converted into a score out of six to allow comparison.

^†^the effect on inequalities is displayed symbolically in the table as: ↓ for an Intervention likely to reduce inequalities: the intervention preferentially improved healthy eating outcomes in people of lower SEP, ↑ for an intervention likely to widen inequalities: the intervention preferentially improved healthy eating outcomes in people of higher SEP, and ↔ for an intervention which had no preferential impact by SEP.

*indicates interventions where statistical significance values were given to the quantitative evidence relevant to our review.

In total, ten of the eighteen “Price” interventions were likely to reduce inequalities by preferentially improving healthy eating outcomes in lower SEPs [34-39]. All six studies reporting interventions which consisted of a combination of taxes and subsidies consistently had a greater impact on lower SEP [37-39]. Two interventions (one subsidy on fruit and vegetables [40] and one tax on high energy density foods [42]) had a greater impact on higher SEP, and there was no differential effect demonstrated in the remaining six studies in the “Price” category [35,37,41].

### Place interventions (environmental measures in specific settings)

Six “Place” interventions were identified. These are summarised in Table 3. Three were carried out in North America [43-45], two in Europe [46,47] and one in New Zealand [48]. Of these, two were school based interventions [46,48], two were work based interventions [44,45], one church based intervention [43] and one area based intervention [47].

**Table 3:** **Summary of “Place”, “Product”, “Prescriptive” and “Promotion” interventions**

**Author**	**Study**	**Setting**	**Intervention**	**Quality** ^**∆**^	**Outcome measured**	**SEP measurement**	**Effect on SEP inequalities** ^**†**^
***Place***							
**Campbell [** 43]	RCT	USA	Church based intervention	5	Mean change in portions of fruit and vegetables consumed	Household income	↔*
**Hughes [** 46 **]**	Cross sectional survey	England	School based intervention	4	Change in portions of fruit and vegetables consumed	Index of Multiple Deprivation	↓*
**Rush** **[** 48 **]**	RCT	New Zealand	School based intervention	3	Change in BMI standard deviation score in 5–7 year olds	Household income	↔*
**Sorenson [** 44 **]**	RCT	USA	Work based intervention	5	% change in those achieving 5 a day	Occupation	↓*
**Sorenson** **[** 45 **]**	RCT	USA	Work based intervention	5	Change in geometric mean grams of fibre per 1000 kcals	Occupation	↓*
**Wendel-Vos** **[** 47 **]**	Cohort study	Holland	Area based intervention	4	Difference in mean energy intake between intervention and control (MJ/d)	Education level	↓*
***Product***							
**Millet** **[** 49 **]**	Observational study	England	Salt reformulation	3	Salt intake (g/d)	Social class	↔*
***Prescriptive***							
*No studies were identified examining the potential SEP differentials effects of restrictions on advertising/marketing through controls or bans; labelling, recommendations or guidelines*							
***Promotion***							
**Cappacci** **[** 50 **]**	Modelling study	UK	Health information campaign (5 a day)	2	Change in fruit and vegetable intake (portions)	Household income	↓*
**Dallongeville** **[** 35 **]**	Modelling study	France	Health information campaign (fruit and vegetable promotion)	2	Change in fruit and vegetable consumption (g/d)	Household income	↔*
**Estaquio** **[** 51 **]**	Cohort study	France	Health information campaign (5 a day)	2	% of males consuming ≥ five portions of fruit and vegetable per day	Education level	↑*
**Stables** **[** 52 **]**	Cross sectional survey	USA	Health information campaign (5 a day)	2	Change in portions of fruit and vegetables consumed	Poverty Index Ratio	↔*

^∆^Quality of empirical studies were assessed using a validated tool [27]. Studies were scored against six criteria and this number was summed to give an overall quality score (maximum of six). The modelling studies were assessed for quality by two independent experts and their scores were converted into a score out of six to allow comparison.

^†^the effect on inequalities is displayed symbolically in the table as: ↓ for an Intervention likely to reduce inequalities: the intervention preferentially improved healthy eating outcomes in people of lower SEP, ↑ for an intervention likely to widen inequalities: the intervention preferentially improved healthy eating outcomes in people of higher SEP, and ↔ for an intervention which had no preferential impact by SEP.

*indicates interventions where statistical significance values were given to the quantitative evidence relevant to our review.

None of the six identified “Place” interventions were judged as likely to widen inequalities, with four likely to reduce inequalities (both work place interventions [44,45], one schools based intervention [46] and one area based intervention [47]).

### Product interventions (modification of food products to make them healthier/less harmful)

Only one “Product” intervention was identified [49]. This intervention is summarised in Table 3. This was a product reformulation intervention conducted in the UK (salt) in which the authors identified no impact by socioeconomic gradient.

### Prescriptive interventions (restrictions on advertising/marketing)

No “Prescriptive” interventions were identified.

### Promotion interventions (mass media public information campaigns)

Four “Promotion” interventions were identified. These are summarised in Table 3. Three of these were conducted in Europe [35,50,51] and one in the USA [52]. All four examined the effectiveness of national “Five a day” health information campaigns. Two studies used modelling methodologies [35,50].

“Promotion” interventions showed mixed results. Two interventions had no preferential impact by SEP [35,52] while one intervention was judged as likely to reduce inequalities [50] and the other intervention judged as likely to widen inequalities [51].

### Person interventions (Individual-based information and education)

Eighteen “Person” interventions were identified. These are summarised in Table 4. The majority of these were conducted in Europe [53-61], eight in the USA [62-68] and one in Australia [69]. Of these, fourteen were health education interventions [53-56,58-60,62,63,65,67-69] and four were dietary counselling interventions [57,61,64,66].

**Table 4:** **Summary of “Person” interventions**

**Author**	**Study**	**Setting**	**Intervention**	**Quality** ^**∆**^	**Outcome measured**	**SEP measurement**	**Effect on SEP inequalities** ^**†**^
**Brownson** **[** 62 **]**	Cross sectional survey	USA	Health education: Community based education	3	% change of the % of people who consume five portions of fruit and vegetables per day	Education level	**↓**
**Burgi** **[** 53 **]**	RCT	Switzerland	Health education: Healthy nutrition program aimed at children	5	Mean BMI (kg/m^2^)	Parental education level	↔*
**Carcaise-Edinboro** **[** 63 **]**	RCT	USA	Health education: Tailored feedback and self-help dietary intervention.	5	Mean fruit and veg intake score (Score out of 3, 3 = less F/V intake, 1 = more F/V intake)	Education level	↓*
**Connett** **[** 64 **]**	RCT	USA	Dietary counselling intervention	3	Change in serum cholesterol (mg/dl)	Household income	**↑**
**Curtis** **[** 54 **]**	Randomised parallel groups comparison study	UK	Health education: Cooking fair with cooking lessons accompanying personalised dietary goal settings	3	% change in mean food energy from fat	Quintile of Deprivation Index	↓*
**Friel** **[** 55 **]**	RCT	Republic of Ireland	Health education: Healthy nutrition program aimed at children (“Hearty heart”)	2	Change in % of children consuming >4 portions of fruit and veg per day	Area level deprivation	↔*
**Haerens** **[** 56 **]**	RCT	Belgium	Health education: adapted computer tailored dietary intervention for children.	4	Change in mean dietary fat intake (g/d)	Education level	↔*
**Havas** **[** 65 **]**	RCT	USA	Health education: Healthy nutrition program aimed at adult women	5	Change in mean daily servings consumed of fruit and vegetables	Education level	↑*
**Havas** **[** 66 **]**	RCT	USA	Dietary counselling intervention	5	% change in fruit and vegetables consumed	Education level	↑*
**Holme** **[** 57 **]**	RCT	Norway	Dietary counselling intervention	5	% change in cholesterol	Social class	**↑**
**Jeffery** **[** 67 **]**	RCT	USA	Health education: Community based education	3	Mean weight change in women (lb)	Household income	↔*
			Health education: Community based education with an additional prize lottery				↔*
**Jouret** **[** 58 **]**	RCT	France	Health education: Healthy nutrition program aimed at children	4	Change in % of children overweight	Area level deprivation	↓*
**Lowe** **[** 59 **]**	Cohort study	UK	Health education: Healthy nutrition program aimed at children	3	% change in vegetables observed consumed	Free school meal entitlement	**↑**
**Plachta-Danielzik** **[** 60 **]**	RCT	Germany	Health education: Healthy nutrition program aimed at children	5	Change in % prevalence of overweight	Parental education level	↑*
**Reynolds** **[** 68 **]**	RCT	USA	Health education: Healthy nutrition program aimed at children	3	Portions of fruit and vegetables consumed	Household income	↑*
**Smith** **[** 69 **]**	RCT	Australia	Health education: Healthy nutrition program aimed at adults	4	Change in fat density consumed (g/4200 kcal)	The Daniel Scale of Occupational Prestige	**↓**
**Toft** **[** 61 **]**	RCT	Denmark	Dietary counselling intervention	4	Change in amount of fruit eaten by men (g/week)	Education level	↑*

^∆^Quality of empirical studies were assessed using a validated tool [27]. Studies were scored against six criteria and this number was summed to give an overall quality score (maximum of six). The modelling studies were assessed for quality by two independent experts and their scores were converted into a score out of six to allow comparison.

^†^the effect on inequalities is displayed symbolically in the table as: ↓ for an Intervention likely to reduce inequalities: the intervention preferentially improved healthy eating outcomes in people of lower SEP, ↑ for an intervention likely to widen inequalities: the intervention preferentially improved healthy eating outcomes in people of higher SEP, and ↔ for an intervention which had no preferential impact by SEP.

*indicates interventions where statistical significance values were given to the quantitative evidence relevant to our review.

“Person” interventions were judged as most likely to widen inequalities, with eight of the eighteen interventions having greater impact in higher SEPs [57,59-61,64-66,68]. All four of the dietary counselling interventions appear likely to widen inequalities.

### Sensitivity analysis

When the screening process was made more selective, the general trends seen in the main Harvest plot were essentially unchanged. “Price” interventions remained the most likely to reduce inequalities, however “Person” interventions now showed mixed results with a more even distribution of effects by SEP when being more selective by only including interventions where statistical significance values were given. There were no differences observed related to the other “P” categories. The addition of studies that split their findings by ethnicity alone [70-77] (making the selection process less selective) had no implications on the main findings (see additional information – Additional file 4). Six of these studies were from the USA, with one from New Zealand and one from the Netherlands.

## Discussion

### Main findings

Interventions categorised by the “6Ps” modified version of the “marketing mix” framework demonstrated differential effects on healthy eating outcomes by socioeconomic position (SEP). “Upstream” interventions categorised as “Price” appeared most likely to decrease health inequalities, while “downstream” “Person” interventions appeared most likely to increase inequalities (this association weakened when only studies which reported significance values pertaining to SEP differential effectiveness were included). No “Prescriptive” interventions were found and only one intervention categorised as “Product” was included. “Place” interventions showed mixed results, although none appeared likely to widen inequalities. However, the vast majority of full text articles which were assessed for eligibility did not explore differential effects by SEP.

### Comparison with other research

This research builds on an earlier systematic review by Oldroyd and colleagues who examined effectiveness of nutrition interventions on dietary outcomes by relative social disadvantage [22]. They concluded that nutrition interventions have differential effects, but could not develop this further due to the small number of studies identified. Our review included 36 studies allowing expansion upon these conclusions. Magnée et al. has recently used a systematic approach exploring the socioeconomic differential impact of lifestyle interventions (including diet) related to obesity prevention in a Dutch setting [78]. They too reported that “downstream” interventions targeting individuals might increase inequalities but their findings were limited by a lack of studies examining socioeconomic differential effects.

Why might “Price” and “Person” interventions affect inequalities differently? White et al. suggest that *how* an intervention is delivered is crucial. Hence structural, universally delivered “upstream” interventions which create a healthier environment therefore tend to circumvent voluntary behaviour change may well reduce inequalities [18]. Frieden depicts this difference as a “Health Impact Pyramid” [79]. The base of the pyramid consists of interventions addressing socio-economic determinants of health which has the greatest potential population impact. Conversely, the top of the pyramid depicts health education and counselling which depend on higher levels of individual effort; hence resulting in the lowest potential population impact. Cappuccio and colleagues likewise found that more “upstream” population-wide regulation and marketing controls had the most potential to reduce dietary salt when compared with more “downstream” approaches like food labelling [80].

Our review supports both White and Frieden [18,79]. Interventions in the “Price” category predominantly included taxes on unhealthy foods and subsidies for healthier foods; both are population level, structural interventions which require no individual agency. This category was the most likely to reduce inequalities. Similar observations have also been demonstrated for tobacco control. Thomas and colleagues found that population level tobacco control interventions, such as increasing the price of tobacco products had a greater potential to benefit more disadvantaged groups and thereby reduce health inequalities [17].

“Person” interventions appeared most likely to widen inequalities. This category included health education and dietary counselling. This may reflect the dependence on an individual choosing to behave differently, and sustain that change [78]. Other studies support this in highlighting that downstream interventions rarely reduce inequalities and may widen them. Whitlock and colleagues reviewed the effectiveness of counselling interventions on public health [81]. This highlighted the lack of effectiveness of these types of interventions on people from across the socioeconomic spectrum. Furthermore, Lorenc et al. explicitly concluded that “downstream” interventions actually worsen health inequalities [82].

It is striking that we did not find any studies investigating the effects of “Prescriptive” interventions by SEP and only one “Product” intervention that presented differential results which had no preferential impact by SEP. Although more “Place” interventions were identified (n = 6), they were conducted in a variety of different settings (2 workplace, 2 school based, 1 in a church and 1 area based intervention). None of these interventions were judged as likely to widen inequalities, however more evidence of a differential impact is required before conclusions can be reached concerning this category.

The potential differential effectiveness of mass media (‘five a day’) campaigns within the “Promotion” category was unclear, as only four studies were found and these showed mixed results.

### Strengths

The systematic approach taken is a considerable strength of this research. And the use of two independent reviewers throughout further strengthened our methodology.

The use of the adapted marketing 4 “Ps” approach provides a simple conceptual framework to categorise and evaluate policy interventions, which may have otherwise been difficult to group.

The adaptation of the Harvest plot using the “6Ps” adaptation of the “4 Ps” marketing mix is a novel approach. Ogilvie and colleagues suggest adapting the Harvest Plot to display differential effectiveness of policy interventions [32]. Our “6P” adaptation highlights the effectiveness of the Harvest plot in displaying heterogeneous results.

Conducting a sensitivity analysis confirmed the general trends seen in the main Harvest plot (Figure 1), with “Price” interventions appearing likely to reduce inequalities. “Person” interventions showed more mixed results, however there remained a predominance of these interventions falling within the widen category.

### Limitations

The evidence base revealed a striking lack of studies quantifying the differential effectiveness of dietary interventions by SEP [83]. We only included interventions where quantitative results by SEP were presented by the author. Differential effects in other studies may have gone unreported. We restricted our search to studies published only in English. This may have meant we failed to identify potentially relevant articles published in another language.

Where possible, we used statistical significance to identify differential effects of interventions. In a number of studies, significance levels were not presented by the study authors (and could not be calculated) and therefore the magnitude of the results was used to determine differential effects. It cannot be inferred that these effects were or were not statistically significant. We therefore conducted a sensitivity analysis which was generally reassuring, while highlighting the lack of available significance levels in the “Person” intervention studies and therefore the need for caution when interpreting these results.

Although the use of the adapted marketing 4 “Ps” approach provides a simple conceptual framework, it should be recognised that a number of the interventions were multicomponent in nature. We categorised interventions based on the underlying theories about how the interventions might have worked to bring about change in healthy eating outcomes. This involved a subjective element, even when using the extended “6Ps” study categorisation. This study categorisation framework could mask the potential differential effectiveness of multicomponent interventions which have substantial elements of two or more “P” categories. Indeed, evidence from tobacco control suggests that comprehensive strategies involving multiple interventions at multiple levels may be more powerful than narrower approaches [84,85].

We did not look at age and sex differences in detail as this was not the focus of this particular paper. However, it represents a potentially important topic for future analyses. Furthermore, the settings in which these interventions are introduced may affect their impact. Low SEP in one setting will differ from low SEP in another setting; likewise with high SEP.

The majority of modelling studies fall in the price category and had weak quality scores reflecting the independent assessment of two modelling experts. This is far from ideal and clearly was very dependent on the assumptions made. While policies to implement price interventions (taxation/subsidies) are difficult to study on a population level, the methods involved with modelling are quite different from an intervention study, and caution should be used when synthesising these different study types. There is an urgent need for the development of a quality assessment tool comparable to those used in empirical studies [27,28].

### Future research

The majority of interventions identified did not present differential results by SEP.

In order to increase knowledge in this area the evaluation of interventions to promote healthy eating should routinely include an assessment of differential effects by SEP. This would enrich the data available to allow for future systematic reviews of this nature to be conducted and to add to the findings presented here [14,15]. Future research should focus in particular upon investigating the differential impact of modification of food products and restrictions on advertising/marketing through controls or bans (“Prescriptive” and “Product” interventions).

Smoking and healthy eating interventions have been assessed for differential effects by SEP. There is a need for comparable studies in other areas such as alcohol and physical activity in order to examine differential impact. In addition, we excluded studies aimed solely at lower SEPs. The examination of these studies is warranted as this will add to our understanding of interventions that may be effective within this sub-group.

In order to further investigate the potential impact of these differential effects, the findings of this review could be tested in epidemiological models for different populations. This would allow quantitative estimations of the socioeconomic effects on disease and mortality burdens in different policy intervention scenarios.

Preventative interventions are more cost effective when compared to treatment [86]. However little is known about the relative cost effectiveness between types of preventative interventions. If an intervention affects different groups differentially, then it is sub-optimally effective in some groups and cannot be achieving its full potential. Its cost-effectiveness will also be sub-optimal. This review suggests interventions aimed at the individual may be less cost-effective, especially among poorer groups, since greater effort and resources may be needed to achieve effectiveness similar to more affluent groups. However, further research in this area is required.

Since the majority of our included “Price” interventions were modelling studies, there is an urgent need to investigate the feasibility and impact of such taxes and subsidies using additional research methods, e.g. RCTs.

Finally, none of the current studies address the more fundamental issue of the inequitable social and economic environments which create health inequalities in the first place [87].

### Policy messages

Policy makers should be aware that some healthy eating interventions targeted at healthy populations may have greater benefits for individuals of higher SEP (and subsequently increase inequalities) notably personalised nutritional education and dietary counselling interventions. On the other hand a combination of taxes and subsidies may preferentially improve healthy eating outcomes for people of lower SEP (potentially reducing inequalities). As noted, the majority of identified studies did not explore differential effects by SEP. When considering implementing a food policy at any level, those involved should consider the potential differential impact of these on health inequalities.

## Additional files

Additional file 1:
**PRISMA – Equity extension.**
Additional file 2:
**Search terms used in MEDLINE search.**
Additional file 3:
**Data extraction tables.**
Additional file 4:
**Sensitivity analysis.**
Additional file 5:
**Summary of included studies (categorised using the 6Ps framework).**
