# Erratum to: Are interventions to promote healthy eating equally effective for all? Systematic review of socioeconomic inequalities in impact

**DOI:** 10.1186/s12889-015-2162-y

**Published:** 2015-09-15

**Authors:** Rory McGill, Elspeth Anwar, Lois Orton, Helen Bromley, Ffion Lloyd-Williams, Martin O’Flaherty, David Taylor-Robinson, Maria Guzman-Castillo, Duncan Gillespie, Patricia Moreira, Kirk Allen, Lirije Hyseni, Nicola Calder, Mark Petticrew, Martin White, Margaret Whitehead, Simon Capewell

**Affiliations:** Department of Public Health and Policy, University of Liverpool, Liverpool, UK; Public and Environmental Health Research Unit, London School of Hygiene and Tropical Medicine, Liverpool, UK; UKCRC Centre for Diet and Activity Research (CEDAR), MRC Epidemiology Unit, University of Cambridge School of Clinical Medicine, Institute of Metabolic Science, Cambridge, UK; Institute of Health and Society, Newcastle University, Newcastle upon Tyne, UK

Unfortunately, the original version of this article [1] contained an error. The description for Figure 1 and Figure 2 have been interchanged. The description attached for Figure 1 has the description meant for Figure 2 and the description attached for Figure 2 has the description meant for Figure 1.
