# Supplementary material for: Are interventions to promote healthy eating equally effective for all? Systematic review of socioeconomic inequalities in impact
Source: BMC Public Health. 2015 May 2;15:457. doi: 10.1186/s12889-015-1781-7 (PMC4423493; doi:10.1186/s12889-015-1781-7)
Supplement: Additional file 2: — Search terms used in MEDLINE search. [file 12889_2015_1781_MOESM2_ESM.docx]

**Additional file 2 – Search terms used in MEDLINE search**

1. (fat adj3 (tax or price or reformulation or prod$ or subsid$ or promot$ or education or label$ or availab$ or reduc$))
2. (salt adj3 (tax or price or reformulation or prod$ or subsid$ or promot$ or education or label$ or availab$ or reduc$))
3. (sugar adj3 (tax or price or reformulation or prod$ or subsid$ or promot$ or education or label$ or availab$ or reduc$))
4. (fibre adj3 (tax or price or reformulation or prod$ or subsid$ or promot$ or education or label$ or availab$ or reduc$))
5. (fruit adj3 (tax or price or reformulation or prod$ or subsid$ or promot$ or education or label$ or availab$ or reduc$))
6. (vegetable adj3 (tax or price or reformulation or prod$ or subsid$ or promot$ or education or label$ or availab$ or reduc$))
7. HEALTH POLICY/
8. DIET/
9. 1 or 2 or 3 or 4 or 5 or 6 or 7 or 8
10. (social adj3 (disparit$ or inequal$ or inequit$ or equit$ or exclude$ or exclusion or include$ or inclusion or status or equality or gradient or hierarchy or class or determinant$))
11. (health adj3 (disparit$ or inequal$ or inequit$ or equit$ or equality or gradient or hierarchy or determinant$))
12. ((economic or material or structural) adj3 (disparit$ or inequal$ or inequit$ or equit$ or exclude$ or exclusion or include$ or inclusion or equality or gradient or hierarchy or class or determinant$))
13. ((socio-economic or socioeconomic) adj3 (disparit$ or inequal$ or inequit$ or equit$ or exclude$ or exclusion or include$ or inclusion or status or equality or gradient or hierarchy or determinant$))
14. (disadvantag$ or vulnerab$ or marginalis$ or depriv$) = 172812 articles
15. (health adj1 gap)
16. VULNERABLE POPULATIONS/
17. SOCIOECONOMIC FACTORS/ OR POVERTY/ OR SOCIAL CLASS/
18. 10 or 11 or 12 or 13 or 14 or 15 or 16 or 17
19. 9 and 18

Items in block capitals indicate MeSH terms; items in lower case indicate free text terms

Limits: English language, publication date 1980 - current
