# Supplementary material for: Are interventions to promote healthy eating equally effective for all? Systematic review of socioeconomic inequalities in impact
Source: BMC Public Health. 2015 May 2;15:457. doi: 10.1186/s12889-015-1781-7 (PMC4423493; doi:10.1186/s12889-015-1781-7)
Supplement: Additional file 3: — Data extraction tables. [file 12889_2015_1781_MOESM3_ESM.docx]

**Additional file 3 - Data extraction tables**

***Price***

| Allais O, Bertail P, Nichèle V: **The Effects of a Fat Tax on French Households' Purchases: A Nutritional Approach.** *American Journal of Agricultural Economics* 2010, **92:**228-245. | |
| --- | --- |
| **Funder(s)** | National Institute of Health and Medical Research (INSRA-INSERM). |
| **“P”** | Price |
| **Aim(s)** | To quantify the relevance and efficacy of a fat tax in France. |
| **Setting** | France |
| **Recruitment and sample size** | Used data from the TNS world panel, which is the principle source of food purchases in France. Each annual survey contains weekly food-acquisition data for approximately 5000 households, with an annual rotation of one third of the participant. Households are selected by stratification by socioeconomic variables and remain in the survey for a mean period of 4 years. |
| **Study design** | Modelling study |
| **Intervention(s) analysed** | 10% tax on food categories high in calories |
| **Nutrient** | Fat |
| **Methods/**  **Intervention details** | Estimates price elasticities using a complete demand system on household scanner data, and by calculating nutrient elasticities using estimated price elasticities. Food stuffs were categorised into broad groups and also translated to their nutrient content. This enabled the proportional effect on nutrient purchase of a proportional change in price (vat) to be computed for different SEGs. |
| **Length of follow up** | NA |
| **SEP measurement** | Household monthly income with respect to the number of members. Four categories – well-off (households with the highest levels of income), average upper (households whose income is above the national average), average lower (households whose income is below the national average) and modest (households with the lowest income). |
| **Primary Outcomes SEP**  % of quantity change in total nutrients purchased for modest and well-off households if particular food groups increase in price by 10%, over a four week period   \|  \| Cheese/butter/cream \| \| Prepared meals \| \| Sugar-fat products \| \| Total of all three \| \| \| --- \| --- \| --- \| --- \| --- \| --- \| --- \| --- \| --- \| \|  \| Well-off \| Modest \| Well-off \| Modest \| Well-off \| Modest \| Well-off \| Modest \| \| Energy \| -1.229 \| -1.167 \| -1.424 \| -1.278 \| -0.789 \| -1.196 \| -3.443 \| -3.641 \| \| Sugar \| -1.831 \| -1.622 \| -0.623 \| -0.564 \| -0.741 \| -1.561 \| -3.195 \| -3.747 \| \| Fat \| -0.913 \| -0.932 \| -1.146 \| -1.224 \| -0.730 \| -1.051 \| -3.058 \| -3.207 \| \| Sat fat \| -1.719 \| -1.723 \| -1.137 \| -0.996 \| -1.461 \| -1.791 \| -4.317 \| -4.510 \| \| Mono fat \| -0.901 \| -0.896 \| -1.642 \| -1.420 \| -0.691 \| -1.029 \| -3.234 \| -3.345 \| \| Poly fat \| 1.127 \| 0.892 \| -1.723 \| -1.419 \| 1.064 \| 0.710 \| 0.467 \| 0.183 \| \| Fibres \| -1.031 \| -1.040 \| -1.085 \| -1.144 \| -1.045 \| -1.502 \| -3.161 \| -3.686 \| \| Sodium \| -1.562 \| -1.499 \| -2.351 \| -2.167 \| -1.502 \| -1.651 \| -5.415 \| -5.317 \| | |
| **Secondary Outcomes SEP** | NA |
| **Primary Outcomes age/sex** | NA |
| **Secondary Outcomes age/sex** | NA |
| **Effect on inequalities** | **The % change in fat is the primary outcome of interest. No indication of significance given for nutrient elasticity by SEP. For the low SEP there was a 3.207% decrease in fat purchased, while for the higher SEP there was a decrease of 3.058%. We conclude that this reduces inequalities.** |
| **Study authors conclusions** | The twin goals of a fat-tax policy are to decrease the sale and consumption of unhealthy foods, and to raise revenue aimed at supporting programs to improve diet and prevent obesity. Based on our findings, we believe that, for the first goal, the impact of a fat tax is small for French consumers; and for the second goal, although such a tax does indeed raise substantial revenues, it is unacceptably regressive. |
| **Limitations** | Estimates maximum potential of fat tax assuming the tax is passed on to consumers in prices and that the food industry and retailers make no other responses |
| **Notes** | Also measured level of education, urbanisation, children’s age and household attributes as descriptive but do not split nutrients purchased by these. They also calculate price and nutrient elasticities by SEG. |

| Cash SB, Sunding DL, Zilberman D: **Fat taxes and thin subsidies: Prices, diet, and health outcomes.** *Food Economics - Acta Agriculturae Scandinavica, Section C* 2005, **2:**167-174. | |
| --- | --- |
| **Funder(s)** | Do not disclose |
| **“P”** | Price |
| **Aim(s)** | To investigate the possible effects of “thin subsidies,” consumption subsidies for healthier foods |
| **Setting** | US |
| **Recruitment and sample size** | The sample population used in this study is the 18,081 individuals over the age of two included in the U.S. Department of Agriculture’s Continuing Study of Food Intakes by Individuals (CSFII) for 1994-1996 and 1998. |
| **Study design** | Modelling study |
| **Intervention(s) analysed** | 1% price change in fruit and vegetables |
| **Nutrient** | Fruit and vegetables |
| **Methods/**  **Intervention details** | Estimated price elasticities from a previous study, which used data from the 1987-88 Nationwide Food Consumption Survey (NFCS). Used 100,000 Monte Carlo trials to calculate induced rates of disease. Assumed fifty % variation in individual dose response functions. The CSFII provides a set of sampling weights that allows for extrapolation of this analysis to the entire U.S. population, i.e., 253.9 million people over two years of age. |
| **Length of follow up** | NA |
| **SEP measurement** | Annual household income: Low = 130% below the poverty level (which was $16,680 for a family of four), High = 300% above the poverty level (included a medium income level but have not indicated what amount of income this equated to). |
| **Primary Outcomes SEP**  Mean (SE) cases of CHD and ischemic stroke (IS) induced in the US population by a 1% price increase in fruit and vegetables:   \| Disease \| All incomes \| Low income \| Medium income \| High income \| \| --- \| --- \| --- \| --- \| --- \| \| All fruits \| \| \| \| \| \| CHD \| 1442 (61.72) \| 231 (28.62) \| 422 (31.69) \| 789 (44.48) \| \| IS \| 744 (33.86) \| 132 (16.17) \| 225 (18.57) \| 386 (23.18) \| \| Total \| 2186 (81.54) \| 363 (38.24) \| 647 (42.91) \| 1175 (57.68) \| \| All vegetables \| \| \| \| \| \| CHD \| 2951 (67.77) \| 528 (28.71) \| 1009 (37.55) \| 1414 (48.61) \| \| IS \| 1482 (37.16) \| 285 (15.68) \| 507 (20.94) \| 690 (26.46) \| \| Total \| 4433 (94.47) \| 813 (40) \| 1516 (52.63) \| 2104 (67.48) \| \| All fruit and vegetables \| \| \| \| \| \| CHD \| 6903 (145.36) \| 1152 (64.03) \| 2260 (78.26) \| 3492 (104.58) \| \| IS \| 3022 (68.25) \| 568 (30.36) \| 997 (37.97) \| 1457 (47.96) \| \| Total \| 9925 (183.52) \| 1720 (81.36) \| 3257 (99.9) \| 4948 (130.92) \|   The authors make the inference that “Because of the relatively linear shape of the dose-response curve over modest consumption changes, the number of reduced cases of disease across each category resulting from a 1 % price subsidy would be almost identical to the results shown here” | |
| **Secondary Outcomes SEP**  Present value of cost per life (numbers are in millions of 2002 US dollars) saved by avoiding heart disease and stroke through 1% subsidy on fruit and vegetables:   \| Food \| All incomes \| Low income \| Middle income \| High income \| \| --- \| --- \| --- \| --- \| --- \| \| Fruit and vegetables \| 1.29 \| 1.02 \| 1.19 \| 1.45 \| \| Fruit \| 2.19 \| 1.82 \| 2.17 \| 2.31 \| \| Vegetables \| 1.8 \| 1.33 \| 1.62 \| 2.12 \| | |
| **Primary Outcomes age/sex** | NA |
| **Secondary Outcomes age/sex** | NA |
| **Effect on inequalities** | **CHD incidence is the primary outcome of interest. Taking the assumption that a tax of the same magnitude would have the same effect in the opposite direction (as the authors have), the high SEP would have a reduction of 3,492 new CHD cases, while the low SEP would have a comparatively lower 1,152 reduction in new CHD cases. Using the SEs to calculate 95% CIs, this gap is significantly different. Therefore we conclude that this increases inequalities.** |
| **Study authors conclusions** | The distributional impacts of such a policy are also worth noting. The CSFII surveys indicate that on average, lower income consumers eat fewer fruits and vegetables. They are therefore more responsive to slight changes in their diets than individuals who consume more fruits and vegetables, again because of the diminishing marginal health benefits of produce consumption. As a result, the cost of saving the life of a low income consumer is almost 30% less than that of a high income consumer. This is both because the intervention is more effective for low income individuals and because they are purchasing less expensive fruits and vegetables. In contrast to the possible regressive effects of a price-increasing regulation, a subsidy would provide the greatest benefits to the most disadvantaged consumers. |
| **Limitations** | Modelling study limitations. The calculations here assume that the entire cost of a price reduction would be covered by government spending. This assumption does not take into account any pre-existing market distortions. For example, it may be the case that trade restrictions or agricultural support programs may already be raising fruit and vegetable prices. If so, some of the reductions in price may be achieved without direct government outlays by reducing the level of the existing distortions. In this case, the actual cost to the government could actually be much lower, although some costs would be borne by other parties currently benefiting from any such distortions. |
| **Notes** |  |

| Dallongeville J, Dauchet L, Mouzon Od, Réquillart V, Soler L-G: **Increasing fruit and vegetable consumption: a cost-effectiveness analysis of public policies.** *The European Journal of Public Health* 2011, **21:**69-73. | |
| --- | --- |
| **Funder(s)** | Does not specify |
| **“P”** | Price |
| **Setting** | France |
| **Aim(s)** | To quantify cost-effectiveness of policies aimed at increasing F&V consumption. Examined two policies: (i) reduction of the consumer price through a decrease in VAT on all F&V and (ii) consumption subsidies through F&V stamps |
| **Recruitment and sample size** | Used fruit and vegetable consumption data from the Individual and National Study on Food Consumption (national population survey in France - INCA). 2,624adults and 1,455 children completed INCA. |
| **Study design** | Modelling study |
| **Intervention(s) analysed** | Policy 1 (P1). Tax reduction on F & V; Policy 2 (P2). Food stamps |
| **Nutrient** | Fruit and vegetables |
| **Methods/**  **intervention details** | Based their economic model on previous French. To estimate subsequent health benefits of consumption data was used from the world cancer research fund and some published meta-analyses. P1. Reduction of tax on F & V from 5.5% to 2.1% (this is the minimum value allowed by the European tax policy). P2. €100/year/person F & V stamp (for comparison with P1 they assumed €465M were used to subsidise F & V consumption of lower income consumers (LIC). Used Monte Carlo simulations drawing 10 million times a 19-uplet of parameters. |
| **Length of follow up** | NA |
| **SEP measurement** | Decile of income (first decile of income is the lowest SEP) |
| **Primary Outcomes SEP** | \| Decile \| P1 \| P2 \| \| --- \| --- \| --- \| \| Consumption variation (g/day) \| \| \| \| All deciles \| 4.8 (3.1-7.1) \| 0.4 (0.2-0.6) \| \| First decile \| 3.4 (1.2-7.5) \| 7 (6-9.2) \| \| Other deciles \| 5 (3.1-7.6) \| -0.3 (-0.5- -0.2) \|   Estimated mean (CI) change in consumption levels of F & V |
| **Secondary Outcomes SEP** | Estimated mean (CI) change in Deaths avoided (DA) and Life years gained (LYS)   \| Decile \| P1 \| P2 \| \| --- \| --- \| --- \| \| Number of Deaths avoided (DA) \| \| \| \| All deciles \| 363 (200-582) \| 77 (48-116) \| \| First decile \| 48 (15-111) \| 99 (62-146) \| \| Other deciles \| 315 (164-526) \| -21 (-37- -10) \| \| Number of Life Years Gained (LYS) \| \| \| \| All deciles \| 5024 (2711-8132) \| 1032 (634-1554) \| \| First decile \| 643 (205-1497) \| 1330 (827-1972) \| \| Other deciles \| 4381 (2226-7368) \| -297 (-519- -140) \| |
| **Primary Outcomes age/sex** | NA |
| **Secondary Outcomes age/sex** | NA |
| **Effect on inequalities** | **Change in mean fruit and vegetable consumption is the primary outcome of interest. For policy 1, both the lowest and other SEPs consume more fruit and veg. However from examining the confidence intervals, these overlap indicating that this is not a significant change in consumption pattern between the two groups. This therefore has no impact on inequalities. For policy 2 however; the confidence intervals do not overlap. In this case the lowest SEP are increasing their consumption of fruit and vegetables by 7g/d while the other SEPs are decreasing their consumption by 0.3g/d. This therefore reduces inequalities.** |
| **Study authors conclusions** | (i) Targeted and non-targeted policies to promote F&V intake have a modest impact on consumption and as a result on health gains, (ii) non-targeted interventions through price modifications appear to be more cost-effective than targeted actions through subsidizing the consumption of the most disfavoured subpopulations. |
| **Limitations** | Limitations associated with modelling studies. Inconsistent data sets used – seem to pluck relevant information from different places and incorporate it. The meta-analyses that the researchers relied upon were subject to criticisms concerning the accuracy of food intake assessment, the quality of event ascertainment, measurement of confounders and publication bias. |
| **Notes** |  |

| Finkelstein Ea ZC: **IMpact of targeted beverage taxes on higher- and lower-income households.** *Archives of Internal Medicine* 2010, **170:**2028-2034. | |
| --- | --- |
| **Funder(s)** | Healthy Eating Research – a national program of the Robert Wood Johnson Foundation. |
| **“P”** | Price |
| **Aim(s)** | To simulate the effects on caloric intake and weight resulting from a 20% or 40% tax on (i) carbonated Sugar Sweetened Beverages (SSBs) only or (ii) carbonated SSBs, fruit drinks and sports/energy drinks simultaneously. |
| **Setting** | Canada |
| **Recruitment and sample size** | Used data from the 2006 Nielsen Homescan Panel – national sample of households that agree to scan and transmit their store bought food and beverage purchases weekly for a 12 month period. Sample used 384252 household months of data. |
| **Study design** | Modelling study |
| **Intervention(s) analysed** | 20% and 40% taxation on carbonated SSBs; 20% and 40% taxation on carbonated SSBs and fruit drinks and sports/energy drinks simultaneously. By checking tax on just carbonated SSBs they can check the cross price elasticity for similar products. By taxing all SSBs they can compare to check if this was the cause for minimal change when taxing only SSBs as other SSBs were purchased to compensate the change in price of the carbonated SSBs. |
| **Nutrient** | Sugar/kilocalories |
| **Methods/**  **intervention details** | To predict the effects of price increases, they used multivariate regression models. The models had two parts: 1^st^ part is a logistic regression that estimates the probability of positive purchases in a given month as a function of average monthly prices in the market for each beverage and other covariates. The 2^nd^ part estimates a regression of the same prices and covariates on log-kcals (per person per day) for those positive purchases. Results were combined to predict daily average beverage kcals purchased and estimate how these changed in response to taxation (20% and 40%). Used model coefficients to estimate reduction in kcal purchased as a result of tax. Each regression controlled for household income. In order to get price effects by income strata, both parts of the model included interaction terms between income quartile and price variables. |
| **Length of follow up** | NA |
| **SEP measurement** | Household income by quartile (0%-25% (lowest income), 26%-50%, 51%-75% and 76%-100%) |
| **Primary Outcomes SEP**  Predicted change in mean energy intake per capita in kcals purchased per day (standard error)*:   \| Effect \| 0%-25% \| 26%-50% \| 51%-75% \| 76%-100% \| All groups \| \| --- \| --- \| --- \| --- \| --- \| --- \| \| Effect of a 20% carbonated SSB tax ↑ on carbonated SSB calories \| -5 (-2.5 to -7.5) \| -5.8 (-4 to -7.8) \| -8 (-6 to -9) \| -5.8 (-4 to -7) \| -6 (0.7) \| \| Effect of a 40% carbonated SSB tax ↑ on carbonated SSB calories \| -9 (-6.5 to -12.5) \| -10 (-7.5 to -13) \| -13.7 (-12 to -15) \| -9 (-7 to -11.8) \| -10.8 (-9.2 to -12) \| \| Effect of a 20% carbonated SSB tax ↑ on all beverage calories \| +0.2 (+4 to -4) \| -9 (-6 to -11.8) \| -8 (-5 to -11.8) \| +1 (+4 to -2) \| -4.2 (-2.5 to -6) \| \| Effect of a 40% carbonated SSB tax ↑ on all beverage calories \| -0.2 (+5.2 to -6) \| -14.5 (-8 to -19) \| -14 (-7.8 to -18) \| +1 (+6 to -5) \| -7.8 (-6.5 to -12) \| \| Effect of a 20% SSB tax ↑ on SSB calories \| -12 (-8 to -14) \| -11.8 (-9 to -13) \| -12.5 (-11 to -14) \| -7.5 (-5.5 to -9) \| -11 (-10 to -12) \| \| Effect of a 40% SSB tax ↑ on SSB calories \| -19 (-15.5 to -24) \| -18.5 (-16 to -22) \| -20 (-17.5 to -23) \| -12.5 (-10 to -15) \| -17.5 (-16 to -19) \| \| Effect of a 20% SSB tax ↑on all beverage calories \| -2.5 (+2.5 to -8) \| -11 (-7 to -14) \| -15 (-11 to -17.5) \| +2 (-5 to +2) \| -7 (-5 to-9) \| \| Effect of a 40% SSB tax ↑on all beverage calories \| -5 (+4 to -14) \| -18.5 (-12.5 to -25) \| -27 (-22 to -37.5) \| +2.5 (+8 to -4) \| -12.7 (-10 to -16) \|   *this information was taken off bar graphs from the paper using a ruler therefore there may be some measurement error. Raw data not given. | |
| **Secondary Outcomes SEP**  Predicted per-person mean weight changes in kg (standard error) (assuming a 3500kcal reduction in energy purchases wquates to 0.45kg of weight lost. **Numbers in bold were significantly different from 0 (p = <0.05)**   \| Tax strategy \| 0-25% \| 26%-50% \| 51%-75% \| 76%-100% \| All groups \| \| --- \| --- \| --- \| --- \| --- \| --- \| \| 20% tax on carbonated SSBs \| 0.01 (0.2) \| **0.37 (0.14)** \| **0.36 (0.14)** \| 0.03 (0.13) \| **0.2 (0.07)** \| \| 40% tax on carbonated SSBs \| 0.004 (0.36) \| **0.68 (0.26)** \| **0.65 (0.25)** \| 0.04 (0.24) \| **0.37(0.13)** \| \| 20% tax on all SSBs \| 0.12 (0.23) \| **0.46 (0.17)** \| **0.68 (0.15)** \| 0.07 (0.15) \| **0.32 (0.09)** \| \| 40% tax on all SSBs \| 0.23 (0.43) \| **0.83 (0.3)** \| **1.2 (0.26)** \| 0.13 (0.28) \| **0.59 (0.16)** \| | |
| **Primary Outcomes age/sex** | NA |
| **Secondary Outcomes age/sex** | NA |
| **Effect on inequalities** | **The change in mean energy intake is the primary outcome of interest. For all four of the simulated tax scenarios, the standard errors indicate that the differences between the values of change in the lowest and highest income quartiles are not significant. Therefore none of the four policies examined has an impact on inequalities.** |
| **Study authors conclusions** | Large taxes on SSBs are likely to be effective at positively influencing weight outcomes, especially among middle-income households. These taxes would also generate substantial revenue that could be used to fund obesity prevention efforts or for other causes. |
| **Limitations** | Data used was self-report – may have underreported. Data was limited to store bought beverage purchases only. If a tax were extended to restaurants and other venues, the effect on SSBs would be greater than those reported here. If this was the case though, consumers could compensate by eating higher caloric foods and the results would therefore be biased. The assumed linear relationship between kcals and weight that was used here to estimate mean weight loss (3500 kcal leads to 0.45 kg lost ) has been described elsewhere as overly optimistic as it does not take into account the body’s compensatory mechanisms that limit long term effects of caloric reductions on body weight. |
| **Notes** |  |

| Nederkoorn C, Havermans RC, Giesen JCAH, Jansen A: **High tax on high energy dense foods and its effects on the purchase of calories in a supermarket. An experiment.** *Appetite*, **56:**760-765. | |
| --- | --- |
| **Funder(s)** | Not stated |
| **“P”** | Price |
| **Aim(s)** | To examine whether a high tax on high calorie dense foods effectively reduces the purchased calories of high energy dense foods in a web based supermarket, and whether this effect is moderated by budget and weight status. |
| **Setting** | Hypothetical supermarket |
| **Recruitment and sample size** | Participants were recruited by advertisements on the internet, using GoogleAds. The advertisements were placed on Dutch websites, using Dutch language. 306 participants fully completed the task online. |
| **Study design** | RCT |
| **Intervention(s) analysed** | Taxation of high energy density (HED) foods |
| **Nutrient** | Energy |
| **Methods/**  **Intervention details** | Participants completed an internet supermarket task which was used to measure food purchasing behaviour. They received the following instructions (translated from Dutch): “Imagine that you have to buy all the food for your entire family for one whole day. You have no food at home and must buy anything your family wants to eat. To this end, you now receive an imaginary budget of €xx that you may spend in the web shop. You do not have to spend your entire budget”. The participants received an idiosyncratic budget to spend in the supermarket; that is the budget they reported to spend on a daily basis. The experiment has a between-subject design and participants were randomly assigned to a condition. In the control condition, normal prices were used, based on prices from the nationally major supermarkets. In the energy density tax condition, all products with a caloric value of more than 300 kcal/100 g were indexed by 50%. This limit was chosen, so that all notoriously fattening foods such as crisps, cookies, chocolate, cheeses, sweets, margarine and butter were indexed, while staple foods (bread, rice, and pasta), fruit and vegetables, and most meats and fish were priced normally. In total, 235 products were taxed, 33% of all available products. The participants were not informed about adjustments of prices and the instructions in the two conditions were completely the same |
| **Length of follow up** | NA |
| **SEP measurement** | Reported daily budget for food - <10€ (lowest SEP); >20€ (highest SEP) |
| **Primary Outcomes SEP** | The effect of budget, being overweight and tax on mean (SEM) purchased calories (kcal) from HED products*:   \|  \| <10€ daily budget \| \| 10-20€ daily budget \| \| >20€ daily budget \| \| \| --- \| --- \| --- \| --- \| --- \| --- \| --- \| \|  \| lean \| overweight \| lean \| overweight \| lean \| overweight \| \| Tax \| 2280 (570) \| 2280 (760) \| 3990 (475) \| 4750 (570) \| 6650 (760) \| 5890 (760) \| \| No tax \| 3705 (570) \| 2470 (570) \| 5320 (380) \| 5510 (380) \| 6840 (760) \| 7600 (950) \| \| Diff due to tax \| 1425 (38.5% change) \| 190 (7.8 % change) \| 1330 \| 760 \| 190 (2.8% change) \| 1710 (22.5% change) \|   *values are interpreted from a graph therefore there is some slight measurement error |
| **Secondary Outcomes SEP** | NA |
| **Primary Outcomes age/sex** | NA |
| **Secondary Outcomes age/sex** | NA |
| **Effect on inequalities** | **After calculating the difference in kcal purchased between tax and no tax for each SEP (see bottom row of table) – it would appear to minimise inequalities, with the lowest SEP purchasing less kcal due to the tax.** |
| **Study authors conclusions** | HED tax appears effective in reducing the purchase of calories, especially from HED foods. In terms of health benefits, this reduction is substantial and equally successful in people with high and low BMI and in people with high and low budgets. The tax appeared effective in reducing calories from carbohydrates, but did not influence the purchased calories from fat. In addition, participants did show a slight tendency to substitute more expensive HED foods for cheaper HED products. |
| **Limitations** | Participants were tested in a ‘closed economy’: they could only choose between different types of food products. In real life, there are far more options in and outside the supermarket, which can alter the effect of a food tax. Disadvantage of an energy density tax is that it cannot be used to tax beverages, since beverages are low in energy density relative to most solid foods. Therefore, other type of taxes might need to be considered |
| **Notes** | Hypothetical situation – very different from an actual supermarket experience. This tax is regressive – i.e. it is affecting those with less daily budget more. |

| Nnoaham KE, Sacks G, Rayner M, Mytton O, Gray A: **Modelling income group differences in the health and economic impacts of targeted food taxes and subsidies.** *International Journal of Epidemiology*, **38:**1324-1333. | |
| --- | --- |
| **Funder(s)** | Do not disclose. |
| **“P”** | Price |
| **Aim(s)** | To examine the effects, by income group, of targeted food taxes and subsidies on nutrition, health and expenditure in the UK. |
| **Setting** | UK |
| **Recruitment and sample size** | Based model on data from the Expenditure and food survey (EFS) |
| **Study design** | Modelling study |
| **Intervention(s) analysed** | Food taxation-subsidy regimens |
| **Nutrient** | Fat, salt, fruit and vegetables |
| **Methods/**  **Intervention details** | Developed a spread sheet-based model to analyse the effects of 4 different taxation-subsidy regimens in the UK: i) 17.5% VAT on foods that are a major source of saturated fats; ii) 17.5% VAT on foods classified as ‘less healthy’ (by the nutrient profiling model “WXYfm“) – these were: yoghurt, cheese, bacon and ham, butter and margarine, sugar and preservatives, processed potatoes, pastries/cakes/biscuits and breakfast cereals. Soft drinks and confectionary are classified as “less healthy” and already attract VAT in the UK, so no further tax was imposed; iii) VAT on foods classified as less healthy combined with a 17.5% subsidy on fruit and vegetables and iv) taxing foods defined as “less healthy” and subsidising fruit and vegetables by 32.5% to neutralise tax revenue. |
| **Length of follow up** | NA |
| **SEP measurement** | Income quintiles in the EFS were based on gross weekly household income. Average weekly income: quintile 1 = £134; quintile 2 = £281; quintile 3 = £475; quintile 4 = £736 and quintile 5 = £1468. |
| **Primary Outcomes SEP**  Income group specific mean (CI) changes in food and nutrient consumption   \| Quintile \| % change in calorie intake \| % change in sat fat intake \| % change in salt intake \| % change in fruit/veg intake \| \| --- \| --- \| --- \| --- \| --- \| \| Scenario i) – sat fat scenario \| \| \| \| \| \| 1 \| -0.53 (-0.65, -0.41) \| -2.54 (-4.69, -0.4) \| 0.28 (-2.37, 2.93) \| -2.81 (-3.65, -1.98) \| \| 2 \| -0.57 (-0.7, -0.44) \| -2.53 (-4.68, -0.38) \| 0.23 (-2.18, 2.64) \| -2.79 (-3.6, -1.2) \| \| 3 \| -0.49 (-0.61, -0.37) \| -2.37 (-4.52, -0.23) \| 0.26 (-2.35, 2.87) \| -2.69 (-3.5, -1.88) \| \| 4 \| -0.5 (-0.63, -0.38) \| -2.14 (-4.23, -0.04) \| 0.25 (-2.35, 2.86) \| -2.63 (-3.44, -1.81) \| \| 5 \| -0.59 (-0.73, -0.46) \| -2.41 (-4.65, -0.17) \| 0.17 (-1.99, 2.33) \| -2.71 (-3.48, -1.94) \| \| Average \| -0.54 (-0.66, -0.41) \| -2.4 (-4.55, -0.24) \| 0.24 (-2.25, 2.73) \| -2.73 (-3.53, -1.76) \| \| Scenario ii) – less healthy food scenario \| \| \| \| \| \| 1 \| -3.08 (-3.38, -2.78) \| -3.66 (-6.25, -1.07) \| -2.29 (-9.94, 5.36) \| -1.54 (-2.16, -0.91) \| \| 2 \| -2.51 (-2.78, -2.24) \| -3.06 (-5.43, -0.68) \| -1.76 (-8.51, 4.98) \| -1.53 (-2.12, -0.93) \| \| 3 \| -2.28 (-2.55, -2.02) \| -2.96 (-5.36, -0.56) \| -1.75 (-8.58, 5.07) \| -1.43 (-2.03, -0.84) \| \| 4 \| -2.11 (-2.37, -1.85) \| -2.89 (-5.33, -0.45) \| -1.77 (-8.76, 5.22) \| -1.37 (-1.97, -0.78) \| \| 5 \| -2.02 (-2.28, -1.77) \| -3 (-5.51, -0.49) \| -1.71 (-8.66, 5.24) \| -1.43 (-1.99, -0.87) \| \| Average \| -2.4 (-2.67, -2.13) \| -3.11 (-5.58, -0.65) \| -1.86 (-8.89, 517) \| -1.46 (-2.05, -0.87) \| \| Scenario iii) – tax plus 17.5% subsidy scenario \| \| \| \| \| \| 1 \| -1.53 (-1.74, -1.32) \| -1.36 (-1.36, -2.92) \| -1.5 (-7.67, 4.67) \| 4.75 (3.67, 5.83) \| \| 2 \| -0.98 (-1.15, -0.82) \| -0.87 (-2.13, 0.38) \| -0.99 (-6.01, 4.04) \| 4.79 (3.76, 5.83) \| \| 3 \| -0.82 (-0.98, -0.66) \| -0.85 (-2.12, 0.42) \| -1 (-6.14, 4.14) \| 4.85 (3.78, 5.93) \| \| 4 \| -0.69 (-0.84, -0.54) \| -0.985 (-2.16, 0.46) \| -1.04 (-6.39, 4.3) \| 4.86 (3.76, 5.95) \| \| 5 \| -0.58 (-0.72, -0.45) \| -0.98 (-2.41, 0.44) \| -0.97 (-6.2, 4.26) \| 4.75 (3.75, 5.76) \| \| Average \| -0.92 (-1.09, -0.76) \| -0.98 (-2.35, 0.38) \| -1.1 (-6.48, 4.28) \| 4.8 (3.74, 5.86) \| \| Scenario iv) – tax plus 32.5% subsidy scenario \| \| \| \| \| \| 1 \| -0.21 (-0.28, -0.13) \| 0.61 (-0.42, 1.65) \| -0.83 (-5.39, 3.74) \| 11.43 (9.816, 13.04) \| \| 2 \| 0.32 (0.23, 0.42) \| 1, -0.33, 2.33 \| -0.32 (-3.18, 2.54) \| 10.97 (9.449, 12.48) \| \| 3 \| 0.44 (0.32, 0.55) \| 0.96 (-0.38, 2.31) \| -0.35 (-3.4, 2.69) \| 11.11 (9.533, 12.68) \| \| 4 \| 0.53 (0.4, 0.66) \| 0.9 (-0.44, 2.24) \| -0.42 (-3.8, 2.96) \| 11.09 (9.491, 12.69) \| \| 5 \| 0.65 (0.51, 0.79) \| 0.74 (-0.48, 1.97) \| -0.34 (-3.44, 2.75) \| 10.16 (8.735, 11.59) \| \| Average \| 0.35 (0.24, 0.46) \| 0.84 (-0.41, 2.1) \| -0.45 (-3.84, 2.94) \| 10.95 (9.4, 12.5) \| | |
| **Secondary Outcomes SEP**  Income group specific changes in health outcomes   \| Quintile \| Change in annual CHD deaths \| \| Change in annual stroke deaths \| \| Change in annual cancer deaths \| \| Change in annual obesity-related CVD deaths \| \| Total deaths (excluding obesity-related CVD deaths) \| \| \| --- \| --- \| --- \| --- \| --- \| --- \| --- \| --- \| --- \| --- \| --- \| \|  \| Worst case \| Best case \| Worst case \| Best case \| Worst case \| Best case \| Worst case \| Best case \| Worst case \| Best case \| \| Scenario i) – sat fat scenario \| \| \| \| \| \| \| \| \| \| \| \| 1 \| -124 \| -158 \| 271 \| 267 \| 307 \| 102 \| -1 \| -16 \| 454 \| 211 \| \| 2 \| -92 \| 120 \| 246 \| 243 \| 334 \| 109 \| -1 \| -15 \| 488 \| 232 \| \| 3 \| -90 \| -114 \| 224 \| 220 \| 307 \| 101 \| 0 \| -11 \| 440 \| 208 \| \| 4 \| -67 \| -87 \| 199 \| 196 \| 295 \| 98 \| 0 \| -11 \| 427 \| 207 \| \| 5 \| -66 \| -87 \| 215 \| 213 \| 354 \| 115 \| 0 \| -11 \| 503 \| 241 \| \| Total deaths \|  \|  \|  \|  \|  \|  \|  \|  \| 2312 \| 1098 \| \| Scenario ii) – less healthy food scenario \| \| \| \| \| \| \| \| \| \| \| \| 1 \| -52 \| -192 \| 146 \| 110 \| 190 \| 42 \| -5 \| -96 \| 284 \| -41 \| \| 2 \| -46 \| -131 \| 141 \| 117 \| 202 \| 52 \| -2 \| -65 \| 296 \| 37 \| \| 3 \| -61 \| -142 \| 127 \| 106 \| 184 \| 47 \| -2 \| -54 \| 250 \| 10 \| \| 4 \| -68 \| -137 \| 111 \| 89 \| 177 \| 45 \| -1 \| -46 \| 219 \| -3 \| \| 5 \| -72 \| -133 \| 125 \| 106 \| 215 \| 58 \| 0 \| -39 \| 268 \| 32 \| \| Total deaths \|  \|  \|  \|  \|  \|  \|  \|  \| 1318 \| 35 \| \| Scenario iii) – tax plus 17.5% subsidy scenario \| \| \| \| \| \| \| \| \| \| \| \| 1 \| -183 \| -288 \| -105 \| -129 \| -39 \| -210 \| -2 \| -48 \| -327 \| -627 \| \| 2 \| -185 \| -243 \| -91 \| -104 \| -37 \| -231 \| -1 \| -26 \| -313 \| -579 \| \| 3 \| -179 \| -236 \| -85 \| -97 \| -35 \| -219 \| -1 \| -19 \| -299 \| -552 \| \| 4 \| -190 \| -241 \| -77 \| -90 \| -34 \| -210 \| 0 \| -15 \| -302 \| -541 \| \| 5 \| -207 \| -251 \| -79 \| -89 \| -37 \| -231 \| 0 \| -11 \| -323 \| -571 \| \| Total deaths \|  \|  \|  \|  \|  \|  \|  \|  \| -1563 \| -2870 \| \| Scenario iv) – tax plus 32/5% subsidy scenario \| \| \| \| \| \| \| \| \| \| \| \| 1 \| -324 \| -392 \| -372 \| -383 \| -124 \| -635 \| 3 \| 0 \| -821 \| -1410 \| \| 2 \| -322 \| -353 \| -318 \| -321 \| -123 \| -654 \| 13 \| 0 \| -763 \| -1329 \| \| 3 \| -298 \| -330 \| -296 \| -299 \| -117 \| -620 \| 15 \| 1 \| -710 \| -1249 \| \| 4 \| -314 \| -346 \| -265 \| -269 \| -114 \| -597 \| 16 \| 0 \| -694 \| -1212 \| \| 5 \| -326 \| -355 \| -256 \| -260 \| -119 \| -621 \| 13 \| 0 \| -702 \| -1236 \| \| Total deaths \|  \|  \|  \|  \|  \|  \|  \|  \| -3689 \| -6435 \| | |
| **Primary Outcomes age/sex** | NA |
| **Secondary Outcomes age/sex** | NA |
| **Effect on inequalities** | **% change in calorie intake is the primary outcome of interest for all four interventions. Scenario i) The change in calorie intake in the low SEG overlaps with the change in calorie intake in the high SEG, therefore this has no impact on inequalities. Scenario ii) The low SEG has a decrease of 3.08% calorie intake while the higher SEG has a decrease of a comparatively lower 2.02%% calorie intake. This is a significant different change between the groups and reduces inequalities. Scenario iii) The low SEG has a decrease of 1.53% calorie intake while the higher SEG has a decrease of a comparatively lower 0.58% calorie intake. This is a significant different change between the groups and reduces inequalities. Scenario iv) The low SEG has a decrease of 0.21% calorie intake while the higher SEG increase their calorie intake by 0.65%. This is a significant different change between the groups and reduces inequalities**. |
| **Study authors conclusions** | Targeted food-related taxes could be optimized by combining them with a subsidy on fruits and vegetables. Adopting a revenue-neutral tax regimen where all revenue from taxing ‘less-healthy foods’ is used to subsidize fruits and vegetables presents the greatest opportunity in terms of health benefits. In considering such tax as a policy option, however, Governments may need to draw on additional research that examines the impact of an increased financial burden on lower-income groups. No clear income group gradients exist in the health gains that will be produced by the combined tax-subsidy regimens. |
| **Limitations** | Assumed a uniform distribution of cancer burden across income groups, but cancer mortality is income related. May have underestimated deaths averted due to a lack of consideration of cross price elasticity – e.g. increasing the price of whole milk will increase the sale of skimmed milk and therefore decrease fat intake and ultimately lead in a greater amount of deaths averted than reported. Food taxes do not influence health behaviour in a linear fashion – other factors e.g., convenience, access, taste preference. The elasticity values in this study were estimated for the period 1988–2000, and may now have changed. Analysis relies on aggregate consumption and expenditure data that may mask important variations within different food categories and income groups. The use of aggregate data also limited the specificity with which some analyses could be performed. Only considers health outcomes with respect to numbers of deaths, and does not calculate disability-adjusted or quality-adjusted life-years lost or gained: in so far as these interventions alter morbidity as well as mortality, the impact of the proposed taxes on overall health outcomes may therefore have been under-estimated. |
| **Notes** |  |

| Sharma A, Hauck K, Hollingsworth B, Siciliani L: **The Effect of Taxing Sugar-Sweetened Beverages across different Income Groups: A Censored Demand Approach.** *Journal of Economic Literature* in press. | |
| --- | --- |
| **Funder(s)** | Not stated |
| **“P”** | Price |
| **Setting** | Victoria, Australia |
| **Aim(s)** | To estimate the changes in consumption of ten non-alcoholic beverages resulting from two taxes that increases the price of sugar-sweetened beverages (SSBs) – the first a 20% tax; the second a 20 cent per litre volumetric tax. |
| **Recruitment and sample size** | Used data from 2011 Homescan panel data 1,390 households were surveyed over one year. The author’s aggregate purchases for each of the ten beverage categories giving four observations for each beverage category taking the total sample size to 5,560 observations. |
| **Study design** | Modelling study |
| **Intervention(s) analysed** | A 20% tax on price and a 20 cents per litre volumetric tax on consumption of ten categories of non-alcoholic beverages and body weight. |
| **Nutrient** | Sugar |
| **Methods/**  **intervention details** | Authors estimated the effect of a SSB tax on SSB consumption – used a demand system for quarterly consumption and compensated for proportion of household consumption by censoring using Heckman-type two-step method. This censored demand approach was estimated using the functional form of the Almost Ideal Demand System (AIDS). The own-price and cross-price elasticities from the censored demand system are then used to derive the impact of 20% SSB tax (assumed to be imposed on regular soft-drinks, cordial and fruit drinks) and a 20 cents per litre volumetric tax on consumption of all ten categories of beverages. This change in consumption was then converted into quantity for each of the ten beverages. These quantities of beverages were then converted into kilo Joules (kJ) equivalent using calorie levels for 8 oz of each non-alcoholic beverages. The change in kJ for each beverage is summed to get total net change in kJ for a person. Using the conversion ratio of 36,500 kJ per kg of body weight, the induced change in kJ per capita was then converted into weight change. |
| **Length of follow up** | NA |
| **SEC measurement** | Household income |
| **Primary Outcomes SEP** | Mean impact of 20% SSB tax on body weight (kg) by income group   \| Beverage \| Low income \| Medium income \| High income \| \| --- \| --- \| --- \| --- \| \| Regular soft drinks \| -0.048 \| -0.045 \| -0.06 \| \| Cordial \| -0.074 \| -0.088 \| -0.075 \| \| Fruit drink \| -0.015 \| -0.009 \| -0.017 \| \| Diet soft drinks \| 0 \| 0 \| 0 \| \| Bottled water \| 0 \| 0 \| 0 \| \| Fruit juice \| 0.006 \| 0.001 \| 0.006 \| \| High-fat milk \| 0 \| 0.001 \| 0 \| \| Low-fat milk \| 0.011 \| 0.004 \| 0.016 \| \| Tea \| 0 \| 0 \| 0 \| \| Coffee \| -0.002 \| -0.001 \| -0.005 \| \| Net change/quarter \| -0.12 \| -0.136 \| -0.135 \| \| Net change/year \| -0.482* \| -0.544* \| -0.541* \|   *=significant at 1, 5 or 10% level |
| **Secondary Outcomes SEP** | Mean impact of 20% SSB tax on consumption (%) by income group   \| Beverage \| Low income \| Medium income \| High income \| \| --- \| --- \| --- \| --- \| \| Regular soft drinks \| -16.68* \| -17.97* \| -18.58* \| \| Cordial \| -53.37* \| -56.02* \| -32.75* \| \| Fruit drink \| -43.36* \| -22.17* \| -19.66* \| \| Diet soft drinks \| 1.35 \| 1.81 \| 0 \| \| Bottled water \| 0 \| 10.71 \| 0 \| \| Fruit juice \| 2.82 \| 0.45 \| 1.75 \| \| High-fat milk \| 0 \| 0.37 \| 0 \| \| Low-fat milk \| 3.23 \| 1.39 \| 4.83 \| \| Tea \| 0.58 \| -0.56 \| -0.07 \| \| Coffee \| -5.07 \| -3.59 \| -16.62 \|   The direct effects are the effect of the SSB tax on SSB consumption; the indirect effect is the substitution effect which arises from substituting consumption to non-SSB beverages or substituting consumption between the three SSB beverages and the total effect is the sum of both the direct and indirect effects.  *=significant at 1, 5 or 10% level |
| **Primary Outcomes age/sex** | NA |
| **Secondary Outcomes age/sex** | NA |
| **Effect on inequalities** | **Change in body weight is the primary outcome of interest. There are significant effects at the year level for all income groups. The high SEG group has a mean decrease of 0.541 kg while the lowest SEG has a decrease of 0.482 kg. This intervention widens inequalities.** |
| **Authors conclusions** | That there is evidence of positive behavioural responses by households to SSB taxes leading to reductions in consumption of SSBs and weight loss. Although heavy purchasers of SSBs belonging to low income groups will have higher health gains (via greater weight reductions), both the 20% SSB tax and volumetric tax are mildly regressive. Note however that, despite its regressivity, SSB taxes count for a very small proportion of per capita income in absolute terms. |
| **Limitations** | Modelling study – simulation of effects rather than an intervention. |
| **Notes** | Also examined the tax burden arising from a SSB tax |

| Smed S, Jensen JD, Denver S: **Socio-economic characteristics and the effect of taxation as a health policy instrument.** *Food Policy* 2007, **32:**624-639. | |
| --- | --- |
| **Funder(s)** | Not stated |
| **“P”** | Price |
| **Aim(s)** | To analyse the quantitative effects of using economic instruments in health policy on the basis of price elasticities calculated from estimated demand systems. |
| **Setting** | Denmark |
| **Recruitment and sample size** | The modelling is based on data collected from weekly household panel data from a representative panel of Danish food consumers (approximately 2000 households). |
| **Study design** | Modelling study |
| **Intervention(s) analysed** | Food taxes and subsidies |
| **Nutrient** | Fat, fibre, sugar, fruit and vegetables |
| **Methods/**  **Intervention details** | The analysis of the impact of food taxes on diet quality is carried out by combining price elasticities, calculated from parameters estimated in econometric models of food demand for five social classes and seven age groups with food/nutrient conversion tables. The authors examine a combination of different policies together in terms of SEG: i) A 5% increase in the price of fatty meats (beef and pork), butter and cheese alongside a VAT reduction on fresh fruit and vegetables, potatoes and grain-based products that was scaled to give a revenue-neutral scenario. ii) Tax on saturated fats (7.89 DKK/kg) alongside subsidies on fibres which were scaled in order to give a revenue-neutral scenario. iii) The same as ii) but with an additional 10.3 DKK/kg tax on sugar. |
| **Length of follow up** | NA |
| **SEP measurement** | Social class - determined from consumers’ self-classification in terms of job position, education, etc. Social classes 1 and 2 represent owners of larger companies, people in leading positions in society and people with higher education; social class 3 represents owners of small firms and white-collar workers with a small number of subordinates or with specialist skills; social class 4 represents white-collar workers without subordinates and skilled blue-collar workers; and social class 5 consists of unskilled blue-collar workers, the unemployed, pensioners, etc. |
| **Primary Outcomes SEP**  Change in nutrient demand (%) for various SEP   \|  \| i) \| \| \| ii) \| \| \| iii) \| \| \| \| --- \| --- \| --- \| --- \| --- \| --- \| --- \| --- \| --- \| --- \| \| Social class \| Sat fat \| Fibre \| Sugar \| Sat fat \| Fibre \| Sugar \| Sat fat \| Fibre \| Sugar \| \| 1 \| -0.3 \| -1.1 \| 19.6 \| 39 \| 8.5 \| -2.7 \| 52.2 \| 12.4 \| 0.6 \| \| 2 \| -5.2 \| 5.6 \| 12.9 \| -13.4 \| 14.1 \| 16.1 \| -10.6 \| 30.3 \| 2.1 \| \| 3 \| -5.2 \| 5.6 \| 12.9 \| -13.3 \| 14.1 \| 16.1 \| -10.8 \| 26.2 \| -4.4 \| \| 4 \| -14.7 \| 3.9 \| 8.2 \| -23.9 \| 8.3 \| 12.9 \| -19.8 \| 22.5 \| -3.2 \| \| 5 \| -10.4 \| 3.3 \| 8.8 \| -22.5 \| 9.1 \| 12.2 \| -13.4 \| 24.1 \| -2.5 \| | |
| **Secondary Outcomes SEP** | NA |
| **Primary Outcomes age/sex**  Change in nutrient demand (%) for various age groups   \|  \| i) \| \| \| ii) \| \| \| iii) \| \| \| \| --- \| --- \| --- \| --- \| --- \| --- \| --- \| --- \| --- \| --- \| \| Age yrs \| Sat fat \| Fibre \| Sugar \| Sat fat \| Fibre \| Sugar \| Sat fat \| Fibre \| Sugar \| \| <26 \| -6 \| 10 \| 6.5 \| -16.4 \| 21.9 \| 8.1 \| -20 \| 36.9 \| -10.4 \| \| 26-29 \| -5.2 \| 4.4 \| 4.2 \| -14 \| 13.7 \| 5.4 \| -12.8 \| 24/3 \| -3 \| \| 30-39 \| -13.8 \| 2 \| 0 \| -40.1 \| 11.9 \| 0 \| -23.1 \| 17.4 \| -21.3 \| \| 40-49 \| -4/9 \| 2.7 \| 2.3 \| -13.3 \| 11.9 \| 5.3 \| -8.3 \| 22.7 \| -15.2 \| \| 50-59 \| 6.8 \| 1 \| -0.1 \| -0.6 \| 7.3 \| -14.3 \| 9.7 \| 15.1 \| -26.1 \| \| 60+ \| -1.9 \| -0.9 \| 4.3 \| -1.3 \| 11.9 \| 18.6 \| 7.1 \| 19.6 \| -7.7 \| | |
| **Secondary Outcomes age/sex** | NA |
| **Effect on inequalities** | **The change in nutrient demand for saturated fat is the primary outcome of interest. Since there are no measurement errors or confidence intervals concerning this information, only a comparison of the magnitude of change between the lowest and highest SEG can be made for each intervention. Intervention i) The lowest SEG decreased their demand for sat fat by 10.4% while the highest SEG decreased their demand for sat fat by 0.3%. Therefore this appears to reduce inequalities, although we cannot infer if the difference is significant. Intervention ii) The lowest SEG decreased their demand for sat fat by 22.5% while the highest SEG increased their demand for sat fat by 39%. Therefore this appears to reduce inequalities, although we cannot infer if the difference is significant. Intervention iii) The lowest SEG decreased their demand for sat fat by 13.4% while the highest SEG increased their demand for sat fat by 52.2%. Therefore this appears to reduce inequalities, although we cannot infer if the difference is significant.** |
| **Study authors conclusions** | Across socio-demographic groups, social classes 4 and 5 generally have the greatest improvements in diet composition in the scenarios considered. Taxation scenarios have an effect on food-choice behaviour, and that it is even possible to design scenarios which are revenue-neutral for the authorities. |
| **Limitations** | The data that the study is based on does not cover purchases from other members of the household who were not the diary keeper, and does not take “food away from home” purchases into consideration; therefore there may be some bias in the results. In this analysis, it has been assumed that the effect of a price change is fully transmitted to the consumers, but there may be a number of barriers to consumer reactions. |
| **Notes** |  |

| Tiffin R, Salois M: **Inequalities in diet and nutrition.** *Proceedings of the Nutrition Society*, **71:**105-111. | |
| --- | --- |
| **Funder(s)** | The Economic and Social Council under the Rural Economy and Land Use Program and by the Medical Research Council under the National Prevention Research Initiative. |
| **“P”** | Price |
| **Aim(s)** | To determine the effectiveness of a policy that uses fat taxes (on unhealthy foods) and thin subsidies (on healthy food) regarding the desirability of tackling health inequalities. |
| **Setting** | UK |
| **Recruitment and sample size** | 6760 households data used from the 2005-2006 UK Expenditure and Food Survey (EFS) |
| **Study design** | Modelling study |
| **Intervention(s) analysed** | Taxation of saturated fat in foods and subsidising fruit and vegetables |
| **Nutrient** | Saturated fat; fruit and vegetables |
| **Methods,**  **Intervention details** | Model used Almost Ideal Demand System. Simulated a tax that increases the price of fatty foods by 1% for every per cent of saturated fats they contain, for instance milk that contains 1.72% of saturated fats will see its price increasing by 1.72%. They put a ceiling on the price increase at 15%. To offset this tax burden and to encourage the consumption of fruit and vegetables, a subsidy of fruit and vegetables is introduced so as to exactly cancel the cost of the fat tax as paid by consumers. |
| **Length of follow up** | NA |
| **SEP measurement** | Occupation (Higher managerial – HM; lower managerial – LM; Workers and technicians – WT; Unemployed – U; Student – S) |
| **Primary Outcomes SEP** | Impacts of a fiscal food policy on selected nutrient intakes (%) across SEP   \| Nutrient \| HM \| LM \| WT \| U \| S \| \| --- \| --- \| --- \| --- \| --- \| --- \| \| Total fats \| -1.71 \| -1.7 \| -1.67 \| -1.56 \| -.155 \| \| SFA \| -2.2 \| -2.13 \| -1.85 \| -1.81 \| -2.06 \| \| MUFA \| -1.81 \| -1.83 \| -1.85 \| -1.83 \| -1.97 \| \| PUFA \| -0.49 \| -0.71 \| -1.06 \| -1.19 \| -0.79 \| \| Energy \| -1.34 \| -1.59 \| -2.08 \| -2 \| -0.68 \| \| Cholesterol \| -2.7 \| -2.8 \| -2.78 \| -2.77 \| -1.64 \| \| Fruit and vegetables \| 10.38 \| 9.81 \| 8.32 \| 9.27 \| 10.42 \| |
| **Secondary Outcomes SEP** | NA |
| **Primary Outcomes age/sex** | NA |
| **Secondary Outcomes age/sex** | NA |
| **Effect on inequalities** | **The change in fruit and vegetable consumption is the primary outcome of interest. Since there are no measurement errors or confidence intervals concerning this information, only a comparison of the magnitude of change between the lowest and highest SEG can be made. The higher/managerial group decrease their energy intake by 1.34% while the unemployed decreased their energy intake by 2%. This seems to decrease inequalities, however we cannot infer if the difference is significant.** |
| **Study authors conclusions** | A tax on food will also have a proportionately larger impact on the poorer segments of society which will worsen as the tax becomes increasingly targeted on the specific elements of the diet which are unhealthy. The subsidy on fruits and vegetables worsens further the redistributive impact rather than helping it. Thus, a fiscally based intervention to address poor diets will contribute to a worsening of economic inequality and does little to address health inequality and may even worsen it if there is a link between the two. |
| **Limitations** | Limitations associated with a modelling study. Did not consider cross-price elasticities Do not quantify the actual amount that the fruit and vegetables are being subsidised by, only that it will cancel out expenditure on taxing of high fat foods – even though within the category of high fat foods there is a gradient of taxation, with foods that are higher in saturated fat content having heavier tax. |
| **Notes** |  |

***Place***

| Campbell MK, Demark-Wahnefried W, Symons M, Kalsbeek WD, Dodds J, Cowan A, Jackson B, Motsinger B, Hoben K, Lashley J, et al: **Fruit and vegetable consumption and prevention of cancer: the Black Churches United for Better Health project.** *American Journal of Public Health* 1999, **89:**1390-1396. | |
| --- | --- |
| **Funder(s)** | National Cancer Institute |
| **“P”** | Place |
| **Aim(s)** | To increase fruit and vegetable consumption by at least 0.5 daily servings in African Americans. |
| **Setting** | 49 black churches in 10 rural counties in eastern North Carolina. |
| **Recruitment and sample size** | All churches with a primarily African American membership (regardless of denomination) were inventories and stratified by size. Each church received $3500. Sample primarily female (73%) and 98% African American. Intervention – n = 1198; delayed intervention – n = 1321. |
| **Study design** | RCT |
| **Intervention(s) analysed** | Church based intervention |
| **Nutrient** | Fruit and vegetables |
| **Methods/**  **Intervention details** | 5 intervention counties, 5 delayed intervention counties. Churches in intervention counties got the intervention immediately whereas those in the delayed condition did not receive any program activities until completion of the 2 year follow up survey. Intervention = multicomponent, ecological framework. Lasted 20 months and used stage of change trans-theoretical model. Intervention included: Individual tailored nutritional advice based on baseline survey, monthly church information packs, help with growing fruit and veg, educational sessions, cookbook and recipe tasting, increasing the presence of fruit and veg offered at church functions. |
| **Length of follow up** | 2 years |
| **SEP measurement** | Education (<HS = less than high school, HS = high school or equivalent, >HS = more than high school) and household annual income (<$20,000 and $20,000+) |
| **Primary Outcomes SEP** | Mean fruit and vegetable consumption (SE) at baseline and 2 year follow up by education and income   \|  \| Intervention \| \| Delayed intervention \| \| Difference at follow up \| P difference \| \| --- \| --- \| --- \| --- \| --- \| --- \| --- \| \| Variable \| Baseline \| Follow up \| Baseline \| Follow up \| \| <HS \| 3.7 (0.12) \| 4.26 (0.17) \| 3.64 (0.13) \| 3.59 (0.18) \| 0.67 (0.19) \| 0.004 \| \| HS \| 3.82 (0.13) \| 4.23 (0.15) \| 3.6 (0.13) \| 3.37 (0.16) \| 0.86 (0.21) \| 0.004 \| \| >HS \| 3.8 (0.13) \| 4.77 (0.20) \| 3.99 (0.12) \| 3.85 (0.19) \| 0.92 (0.22) \| 0.001 \| \| <$20,000 \| 3.82 (0.1) \| 4.48 (0.14) \| 3.59 (0.1) \| 3.63 (0.15) \| 0.84 (0.17) \| 0.001 \| \| $20,000+ \| 3.64 (0.14) \| 4.23 (0.13) \| 3.79 (0.14) \| 3.64 (0.12) \| 0.6 (0.18) \| 0.014 \|   All comparisons at follow up were statistically significant (largest p = 0.014) |
| **Secondary Outcomes SEP** | NA |
| **Primary Outcomes age/sex** | Mean fruit and vegetable consumption (SE) at baseline and 2 year follow up by gender and age   \|  \| Intervention \| \| Delayed intervention \| \| Difference at follow up \| \| --- \| --- \| --- \| --- \| --- \| --- \| \| Variable \| Baseline \| Follow up \| Baseline \| Follow up \| \| Male \| 3.31 (0.11) \| 3.66 (0.13) \| 3.22 (0.11) \| 3.16 (0.14) \| 0.5 (0.19) \| \| Female \| 3.96 (0.11) \| 4.7 (0.16) \| 3.87 (0.11) \| 3.84 (0.16) \| 0.86 (0.17) \| \| 18-37 \| 3.45 (0.16) \| 3.55 (0.16) \| 3.21 (0.16) \| 3.08 (0.2) \| 0.47 (0.23) \| \| 38-51 \| 3.66 (0.15) \| 4.49 (0.21) \| 3.51 (0.16) \| 3.51 (0.22) \| 0.98 (0.29) \| \| 52-65 \| 3.87 (0.12) \| 4.44 (0.16) \| 3.92 (0.12) \| 3.85 (0.16) \| 0.58 (0.22) \| \| 66+ \| 4.06 (0.11) \| 4.88 (0.12) \| 3.95 (0.11) \| 3.85 (0.13) \| 1.04 (0.17) \|   All comparisons at follow up were statistically significant (largest p = 0.013) except for age 18-37 (p = 0.041). |
| **Secondary Outcomes age/sex** | NA |
| **Effect on inequalities** | **The mean change in fruit and vegetable intake is the primary outcome of interest. The intervention had a similar effect on the low and high SEGs with the amount of change in consumption overlapping between the groups. This intervention had no impact on inequalities**. |
| **Study authors conclusions** | Showed positive results in achieving dietary behaviour change I(observed effect size = 0.85 servings is greater than the projected 0.5 servings). Those aged 18-35 and those who are single should be the focus of future research as they failed to make a dietary behaviour change in these two sub groups of the population. |
| **Limitations** | Self-report. The outcome measure used a brief FFQ rather than a more comprehensive dietary instrument that might have assess additional fruit and vegetables consumed and the contribution of mixed dishes. Study was too shirt to assess the changes in cancer development therefore they didn’t ask about cancer and other diseases. |
| **Notes** |  |

| Hughes RJ, Edwards KL, Clarke GP, Evans CEL, Cade JE, Ransley JK: **Childhood consumption of fruit and vegetables across England: a study of 2306 6–7-year-olds in 2007.** *British Journal of Nutrition* 2012, **108:**733-742. | |
| --- | --- |
| **Funder(s)** | National Prevention Research Initiative. |
| **“P”** | Place |
| **Aim(s)** | To explore the geographic and demographic variations in the uptake of the School Fruit and Vegetable Scheme (SFVS) and the amount of fruit and vegetables that children consume on a daily basis. |
| **Setting** | England |
| **Recruitment and sample size** | The dietary data were collected from children in their third year of school from a randomly selected sample of government-run schools in England containing pupils in the third to fifth years of school with a minimum year group size of fifteen pupils. Independent schools, special schools, schools without all three year groups, and small schools with fewer than fifteen pupils per year group were excluded. In order to reduce bias, schools connected with previous SFVS studies were excluded from the selection process. 2306 children in total (128 schools). The number of boys and girls sampled were approximately equal. |
| **Study design** | Cross sectional survey |
| **Intervention(s) analysed** | School based intervention |
| **Nutrient** | Fruit and vegetables |
| **Methods/**  **Intervention details** | Dietary data were collected using a 24 h FFQ, known as the Child and Diet Evaluation Tool (CADET). |
| **Length of follow up** | NA |
| **SEP measurement** | The UK Index of Multiple Deprivation was used as the measure of deprivation at Lower Layer Super Output Area level as calculated from the child’s postcode. The deprivation score data were divided into ten groups, ranging from least deprived (group 1) to most deprived (group 10). |
| **Primary Outcomes SEP**   \| Deprivation category group (n) \| % of children participated in SFVS \| % of children who consumed at least 5 f & v p/d* \| % of children who consumed at least 5 f & v p/d† \| Daily frequency of f & v consumed* \| \| Daily frequency of f & v consumed† \| \| \| --- \| --- \| --- \| --- \| --- \| --- \| --- \| --- \| \| Mean \| 95% CI \| Mean \| 95% CI \| \| 1 (231) \| 58.9 \| 67.1 \| 59.7 \| 5.9 \| 5.6 to 6.2 \| 5.2 \| 4.9 to 5.5 \| \| 2 (225) \| 47.1 \| 59.1 \| 51.1 \| 5.4 \| 5 to 5.7 \| 4.8 \| 4.5 to 5.1 \| \| 3 (236) \| 60.2 \| 69.9 \| 61 \| 6 \| 5.7 to 6.3 \| 5.4 \| 5 to 5.7 \| \| 4 (228) \| 59.2 \| 68.4 \| 58.8 \| 5.8 \| 5.5 to 6.1 \| 5.1 \| 4.8 to 5.5 \| \| 5 (233) \| 66.1 \| 58.8 \| 49.4 \| 5.4 \| 5.1 to 5.7 \| 4.7 \| 4.4 to 5 \| \| 6 (228) \| 60.5 \| 60.1 \| 50 \| 5.5 \| 5.2 to 5.9 \| 4.9 \| 4.5 to 5.2 \| \| 7 (233) \| 62.2 \| 57.1 \| 44.2 \| 5.3 \| 4.9 to 5.6 \| 4.5 \| 4.2 to 4.9 \| \| 8 (230) \| 69.1 \| 54.8 \| 43 \| 5.3 \| 4.9 to 5.6 \| 4.5 \| 4.1 to 4.8 \| \| 9 (228) \| 61.8 \| 50.9 \| 39.5 \| 5 \| 4.6 to 5.3 \| 4.3 \| 3.9 to 4.6 \| \| 10 (234) \| 70.1 \| 45.3 \| 31.2 \| 4.5 \| 4.2 to 4.8 \| 3.7 \| 3.3 to 4 \|   *Including fruit and vegetables from the SFVS; †Excluding fruit and vegetables from the SFVS | |
| **Secondary Outcomes SEP**  The authors also conducted a geodemographic analysis using output area analysis (OAC). This was used at group level to identify groups of children. Children from the following groups were more likely to participate in the scheme: Younger blue collar; Settled in the city; Prospering semis; Thriving suburbs; Senior communities; Public housing; Settled households; Young families in terraced homes; Asian communities; Afro-Caribbean communities (these are described as areas of less advantage by the authors). Children were more likely to consume fruit or vegetables five times or more per d if they were from the following groups: Settled in the city; Village life; Agricultural; Accessible countryside; Prospering older families; Thriving suburbs; Settled households; Least divergent; Young families in terraced homes; Aspiring households; Asian communities. The area type with the greatest segmentation index was Thriving suburbs, which is characterised by high rates of car ownership and owner-occupied detached housing. | |
| **Primary Outcomes age/sex** | NA |
| **Secondary Outcomes age/sex** | NA |
| **Effect on inequalities** | **The mean daily frequency of fruit and vegetables consumed is the primary outcome of interest. The most deprived children had significantly higher daily intake of fruit and vegetables than the least deprived children (when including and excluding the additional fruit given in the intervention). This intervention reduces inequalities.** |
| **Study authors conclusions** | The SFVS is positively associated with dietary fruit and vegetable intake in young children, particularly those who live in deprived areas. Although the SVFS does not eliminate the socio-economic gradient in fruit and vegetable consumption, it does help to increase fruit and vegetable consumption in deprived (and affluent) areas. |
| **Limitations** | The use of CADET – self report and this only covers a child’s intake for one day therefore preventing longitudinal comparisons from being made. Only government run schools were included in the study – results may not be applicable to other types of school, in England. The use of postcode to categorise deprivation has come under criticism as it may give misleading impressions of areas and individuals. Since the data is cross-sectional, no causal relationships can be established.. |
| **Notes** |  |

| Rush E, Reed P, McLennan S, Coppinger T, Simmons D, Graham D: **A school-based obesity control programme: Project Energize. Two-year outcomes.** *British Journal of Nutrition*, **107:**581-587. | |
| --- | --- |
| **Funder(s)** | The Waikato District Health Board. |
| **“P”** | Place |
| **Aim(s)** | To compare changes in blood pressure (BP) and body composition in children who attended Energize schools with children in control schools. The trial also aimed to identify predictors of increase in body fat and BP over 2 years in relation to age, sex, ethnicity, rurality and social deprivation. |
| **Setting** | Primary schools in Waikato, New Zealand |
| **Recruitment and sample size** | 124 schools were randomised with stratification by rurality and SEG as either a control (n = 660) or to receive the intervention (n = 692). The evaluation sampled children who were 5 or 10 years of age, before programme commencement |
| **Study design** | RCT |
| **Intervention(s) analysed** | School based intervention |
| **Nutrient** | Multiple/not specified |
| **Methods/**  **Intervention details** | The intervention was delivered by ‘Energizers’ who each supported between eight and ten schools. Energizers promoted active transport, lunchtime games, bike days and leadership training for students to be leaders of physical activities before and after school. Weekly newsletters were used to promote healthy alternate ‘food swaps’. There was also a home–school link programme that provided opportunities for parents to attend three information-based sessions, which included a 45 min practical nutrition class. During the same weeks, all classes in the school received integrated nutrition lessons. Fridge magnets and laminated cards that reinforced the nutrition goals were provided to all children, and they were encouraged to put these on their fridges at home. There was also professional development and evenings with a dietitian to raise parents’ awareness of healthier choices of food. Activities also targeted the local community through events such as gala open days and edible gardens. Control schools were given no additional resourcing or information; however, no restrictions were placed on initiatives they may have pursued for themselves. Each school involved in the project worked with their Energizer to develop an individualised action plan based on the individual needs of the school. |
| **Length of follow up** | The intervention lasted 2 years. |
| **SEP measurement** | Household income |
| **Primary Outcomes SEP**  Differences in outcome variables in the 2 year intervention (intervention relative to the control, adjusted for confounding variables) – I = intervention   \|  \| 5-7 year old sample \| \| \| \| 10-12 year old sample \| \| \| \| \| --- \| --- \| --- \| --- \| --- \| --- \| --- \| --- \| --- \| \|  \| Effect of I \| 95% CI \| P* \| P† \| Effect of I \| 95% CI \| P* \| P† \| \| BMI standard deviation score \| \| \| \| \| \| \| \| \| \| European \| -0.03 \| -0.1, 0.04 \| 0.46 \| 0.64 \| 0.01 \| -0.1, 0.12 \| 0.82 \| 0.83 \| \| Maori \| 0.07 \| -0.08, 0.21 \| 0.35 \| 0.39 \| 0.1 \| -0.05, 0.25 \| 0.18 \| 0.25 \| \| Other \| -0.03 \| -0.31, 0.24 \| 0.81 \| 0.84 \| -0.05 \| -0.46, 0.35 \| 0.79 \| 0.74 \|   *adjusted for baseline measures, rurality and school SEG decile (including interaction); †P value corrected for the clustering of children by school | |
| **Secondary Outcomes SEP**  Differences in outcome variables in the 2 year intervention (intervention relative to the control, adjusted for confounding variables) – I = intervention   \|  \| 5-7 year old sample \| \| \| \| 10-12 year old sample \| \| \| \| \| --- \| --- \| --- \| --- \| --- \| --- \| --- \| --- \| --- \| \|  \| Effect of I \| 95% CI \| P* \| P† \| Effect of I \| 95% CI \| P* \| P† \| \| Systolic blood pressure standard deviation score \| \| \| \| \| \| \| \| \| \| European \| -0.07 \| -0.23, 0.1 \| 0.41 \| 0.52 \| -0.34 \| -0.59, -0.09 \| 0.008 \| 0.05 \| \| Maori \| 0.1 \| -0.2, 0.39 \| 0.53 \| 0.56 \| 0.1 \| -0.32, 0.53 \| 0.63 \| 0.68 \| \| Other \| 0.52 \| 0.02, 1.02 \| 0.04 \| 0.04 \| -0.73 \| -1.56, 0.1 \| 0.08 \| 0.11 \| \| Diastolic blood pressure standard deviation score \| \| \| \| \| \| \| \| \| \| European \| 0 \| -0.14, 0.13 \| 0.98 \| 0.98 \| -0.16 \| -0.35, 0.03 \| 0.09 \| 0.15 \| \| Maori \| 0.15 \| -0.13, 0.42 \| 0.28 \| 0.27 \| -0.08 \| -0.46, 0.03 \| 0.66 \| 0.69 \| \| Other \| 0 \| -0.44, 0.43 \| 0.99 \| 0.98 \| -0.25 \| -0.96, 0.47 \| 0.48 \| 0.46 \|   *adjusted for baseline measures, rurality and school SEG decile (including interaction); †P value corrected for the clustering of children by school | |
| **Primary Outcomes age/sex**  Differences in outcome variables in the 2 year intervention (intervention relative to the control, adjusted for confounding variables) – I = intervention   \|  \| 5-7 year old sample \| \| \| \| 10-12 year old sample \| \| \| \| \| --- \| --- \| --- \| --- \| --- \| --- \| --- \| --- \| --- \| \|  \| Effect of I \| 95% CI \| P* \| P† \| Effect of I \| 95% CI \| P* \| P† \| \| BMI standard deviation score \| \| \| \| \| \| \| \| \| \| Boys \| -0.01 \| -0.1, 0.09 \| 0.9 \| 0.92 \| 0.02 \| -0.1, 0.13 \| 0.78 \| 0.81 \| \| Girls \| 0 \| -0.08, 0.08 \| 0.98 \| 0.98 \| 0.08 \| -0.05, 0.21 \| 0.2 \| 0.24 \|   *adjusted for baseline measures, rurality and school SEG decile (including interaction); †P value corrected for the clustering of children by school | |
| **Secondary Outcomes age/sex**  Differences in outcome variables in the 2 year intervention (intervention relative to the control, adjusted for confounding variables) – I = intervention   \|  \| 5-7 year old sample \| \| \| \| 10-12 year old sample \| \| \| \| \| --- \| --- \| --- \| --- \| --- \| --- \| --- \| --- \| --- \| \|  \| Effect of I \| 95% CI \| P* \| P† \| Effect of I \| 95% CI \| P* \| P† \| \| Systolic blood pressure standard deviation score \| \| \| \| \| \| \| \| \| \| Boys \| 0.04 \| -0.15, 0.23 \| 0.65 \| 0.69 \| -0.43 \| -0.75, -0.11 \| 0.009 \| 0.06 \| \| Girls \| 0 \| -0.2, 0.2 \| 0.99 \| 0.99 \| -0.01 \| -0.26, 0.25 \| 0.97 \| 0.97 \| \| Diastolic blood pressure standard deviation score \| \| \| \| \| \| \| \| \| \| Boys \| 0.04 \| -0.11, 0.2 \| 0.58 \| 0.62 \| -0.31 \| -0.56, -0.06 \| 0.01 \| 0.06 \| \| Girls \| 0.02 \| -0.16, 0.2 \| 0.84 \| 0.85 \| 0.05 \| -0.19, o,28 \| 0.7 \| 0.66 \|   *adjusted for baseline measures, rurality and school SEG decile (including interaction); †P value corrected for the clustering of children by school | |
| **Effect on inequalities** | **The change in 5-7 year old change in BMI is the primary outcome of interest. The intervention did not have a significant effect on either SEG. This intervention had no impact on inequalities.** |
| **Study authors conclusions** | Project Energize supports studies elsewhere that schools are important arenas to implement health promotion strategies to prevent overweight and obesity. Although this intervention programme, at best, has shown minor improvements in the health outcomes over 2 years, its potential long-term benefits cannot be ignored. |
| **Limitations** | Attrition over time meant absolute numbers, particularly for Maori, fell below that required for significance. While the evaluation measurements were undertaken 2 years from the commencement of the intervention, the nature of the intervention process meant that it was able to be implemented only in a graduated way, reflecting the characteristics and capacities of individual schools. This led to a shorter duration of intervention implementation before endpoint measurements for lower-decile schools, where a higher proportion of Maori children attend. This, along with the relatively low absolute numbers of Maori children enrolled in the evaluation and subsequent lack of power, may account for the lack of demonstrated effect for Maori in subgroup analysis. Direct measures of changes in activity levels and dietary habits were not made due to budget and time restraints. Children’s behaviours outside of the school environment could also not be accounted for and the present study may not be truly representative of all children in all schools in the Waikato region of New Zealand. Control schools were not limited in any other initiatives that they may have wanted to undertake and at that time, the nationwide Healthy Eating Healthy Action Strategy with the ‘Mission On’ initiative targeted at young New Zealanders was introduced. This initiative was supported by social marketing, monitored food regulations and supplied fruit to low-SES schools. |
| **Notes** |  |

| Sorensen G, Stoddard A, Hunt MK, Hebert JR, Ockene JK, Avrunin JS, Himmelstein J, Hammond SK: **The effects of a health promotion-health protection intervention on behavior change: the WellWorks Study.** *American Journal of Public Health* 1998, **88:**1685-1690. | |
| --- | --- |
| **Funder(s)** | National Cancer Institute and the Liberty Mutual Group |
| **“P”** | Place |
| **Aim(s)** | To test the effect of an integrated program on changes in dietary habits (consumption of fat, fibre, fruit and veg) and smoking. Also to assess whether the intervention effect differed by job category. |
| **Setting** | Businesses throughout the USA that use known or suspected carcinogens in work processes. |
| **Recruitment and sample size** | Recruited worksites from a Dun and Bradstreet listing. They needed to have had 250-2500 employees, a turnover rate of <20% and have <20% non-English speaking employees. 24 worksites – 12 pairs chosen for comparability (e.g. distribution of blue and white collar workers, smoking policy etc). 2368 workers in the analysis. |
| **Study design** | RCT |
| **Intervention(s) analysed** | Work based intervention |
| **Nutrient** | Fat, fibre, fruit and vegetables |
| **Methods/**  **Intervention details** | Based on a social ecological model. Had 3 elements targeting behaviour change: i) joint worker-management participation in program planning and implementation, operationalised through an employee advisory board and a designated work-site liaison. ii) Consultation by project staff with management on work-site environmental changes including tobacco control policies, increased availability of healthy foods, and reduction in the potential exposure to occupational hazards. iii) Health education programs targeting individual behaviours in each of the risk factor areas. Diet was assessed using FFQs. Random sample of workers from each worksite were selected for analysis. |
| **Length of follow up** | 2 years |
| **SEP measurement** | Job category |
| **Primary Outcomes SEP** | Adjusted geometric mean grams of fibre per 1000 kcals by job category controlling for significant covariates.   \| Job category \| Survey \| Intervention \| Control \| \| --- \| --- \| --- \| --- \| \| Skilled/unskilled labour \| Baseline \| 7.42 \| 7.67 \| \|  \| Final \| 8.31 \| 8.03 \| \|  \| Change (%)* \| +12 \| +5 \| \| Office work \| Baseline \| 7.77 \| 7.55 \| \|  \| Final \| 7.88 \| 7.84 \| \|  \| Change (%) \| +1 \| +4 \| \| Professional/managerial \| Baseline \| 8.09 \| 8.01 \| \|  \| Final \| 8.56 \| 8.58 \| \|  \| Change (%) \| +6 \| +7 \|   *p = <0.05 for intervention v control |
| **Secondary Outcomes SEP** | NA |
| **Primary Outcomes age/sex** | NA |
| **Secondary Outcomes age/sex** | NA |
| **Effect on inequalities** | **The adjusted geometric mean grams of fibre is the primary outcome of interest. The lower SEP achieved a significantly higher level of fibre consumption than the control group, whereas there was no significant difference between the higher SEPs and their control counterparts. This intervention reduces inequalities.** |
| **Study authors conclusions** | This provides preliminary evidence from a RCT that such a program may effective in producing meaningful population changes in behavioural risk factors particularly among=g blue collar workers. |
| **Limitations** | Worksites were only randomised by intervention v control – they were not randomly selected for the study. These results can only be generalised to other worksites of similar strict criteria 3.g. the use of carcinogenic materials. Non responders significantly differed from those who did not drop out in smoking status. |
| **Notes** |  |

| Sorensen G, Linnan L, Hunt MK: **Worksite-based research and initiatives to increase fruit and vegetable consumption.** *Preventive Medicine: An International Journal Devoted to Practice and Theory* 2004, **39:**S94-S100. | |
| --- | --- |
| **Funder(s)** | National Cancer Institute |
| **“P”** | Place |
| **Aim(s)** | To examine the efficacy of a cancer prevention intervention designed to improve health behaviours among working-class, multiethnic populations employed in small manufacturing businesses. |
| **Setting** | Worksites in the greater Boston area of Massachusetts, USA. |
| **Recruitment and sample size** | Used Dan and Bradstreet database to identify worksites coded with standard industrial codes and with 50 – 150 employees. Additional criteria were that they had to be multi-ethnic, had a turnover rate <20% in the last year. 26 worksites agreed to take part, n = 1740 at baseline. 931 control, 806 intervention. 2 worksites dropped out (1 intervention, 1 control) leaving 974 participants for analysis. |
| **Study design** | RCT |
| **Intervention(s) analysed** | Work based intervention |
| **Nutrient** | Fruit and vegetables |
| **Methods/**  **Intervention details** | This was part of the Harvard Cancer Prevention Program Project. 13 sites randomised to intervention, 13 to control. Intervention was based on principles of employee participation and a social-context framework targeting multiple influences of behaviour. Special attention to low literacy and focusing on the shared and unique features of different cultures. Intervention was at the worksite level rather than the individual level. Over the 18 months, there was monthly intervention activities that focused on individual change and management were contacted once a month regarding environmental support. Ran health fairs and gave educational materials to employees and their families. Changed food offered at meetings and events to healthy food and changed smoking and hazardous work conditions policies. |
| **Length of follow up** | 18 months |
| **SEP measurement** | Occupation – Managers and workers |
| **Primary Outcomes SEP**  Adjusted* % (n) of participants reporting 5 or more servings of fruits and vegetables per day   \| Occupational class \| Control \| \| \| Intervention \| \| \| \| --- \| --- \| --- \| --- \| --- \| --- \| --- \| \|  \| Baseline \| Follow up \| % change \| Baseline \| Follow up \| % change \| \| Managers \| 8.2 (12) \| 11.8 (11) \| +3.6 \| 20.1 (28) \| 14.6 (14) \| -5.5 \| \| Workers \| 12.7 (91) \| 13.8 (95) \| +1.1 \| 14.3 (92) \| 21.8 (115) \| +7.5 \|   *adjusted for clustering of workers in worksites  **p=0 .048 for difference between intervention and control condition for both managers and workers. | |
| **Secondary Outcomes SEP** | NA |
| **Primary Outcomes age/sex** | NA |
| **Secondary Outcomes age/sex** | NA |
| **Effect on inequalities** | **Change in the number of people achieving five portions of fruit and vegetables per day is the primary outcome of interest. The higher SEP had a significantly lower consumption rate than the control group, whereas the lower SEP achieved a significantly higher reported fruit and vegetable consumption rate than their control counterparts. This intervention reduces inequalities.** |
| **Study authors conclusions** | The social-context model holds promise for reducing disparities in cancer-related health behaviours. |
| **Limitations** | Results are not generalisable to other worksites due to the specific set of criteria each had to have to have taken part. Study has enough power to detect differences between intervention and control conditions, but did not have enough power to detect differences among sub groups. Results of tests for effect modification by indicators of SES must therefore be interpreted with caution. The effectiveness of the intervention may have been limited by features inherent in the worksite setting, e.g. “work-time” for worker to participate was not universally available and in some cases, management were hesitant to commit resources to changing the work environment to support workers health |
| **Notes** |  |

| Wendel-Vos GCW, Dutman AE, Verschuren WMM, Ronckers ET, Ament A, van Assema P, van Ree J, Ruland EC, Schuit AJ: **Lifestyle Factors of a Five-Year Community-Intervention Program: The Hartslag Limburg Intervention.** *American Journal of Preventive Medicine* 2009, **37:**50-56. | |
| --- | --- |
| **Funder(s)** | The Netherlands Organization for Health Research and Development, The ministry of public health, welfare and sports of the Netherlands and the Environment and the National Institute of Public Health. |
| **“P”** | Place |
| **Aim(s)** | To investigate the effect of Hartslag Limburg on lifestyle factors after 5 years of the intervention. |
| **Setting** | Maastricht |
| **Recruitment and sample size** | Cohort used to investigate the net effect of the interventions – taken from intervention and control areas in and around Maastricht. |
| **Study design** | Cohort study |
| **Intervention(s) analysed** | Area based intervention |
| **Nutrient** | Multiple |
| **Methods/**  **Intervention details** | Hartslag Limburg = a large umbrella project comprising a total of 790 interventions of which 590 were major (193 dietary, 361 physical activity and 9 anti-smoking). Examples of these are nutrition parties, debt assistance (people taught to cook healthy meals on a budget), billboards, posters, TV guided aerobics programmes etc. Almost 50% of the interventions took place in deprived areas. Integrated 2 strategies – upstream and downstream. Participants in the cohort completed FFQs at baseline and 5 years later. |
| **Length of follow up** | 5 years |
| **SEP measurement** | Education level (low = intermediate secondary education or less, moderate = intermediate vocational or higher secondary education, high = higher vocational education or university) |
| **Primary Outcomes SEP** | Mean change in diet after 5 years of intervention and adjusted difference* (SE) in change stratified by education level   \| Factor \| Low education level \| \| \| Moderate/high education level \| \| \| \| --- \| --- \| --- \| --- \| --- \| --- \| --- \| \|  \| Interv \| Control \| Adj. diff \| Interv \| Control \| Adj diff \| \| Energy intake (MJ/d) \| -0.6 \| -0.4 \| -0.2 (0.12)** \| -0.6 \| -0.4 \| -0.2 (0.1) \| \| Fat intake (g/d) \| -7 \| -4 \| -3 (1.46)** \| -6 \| -4 \| -2.5 (1.2) \| \| Sat fat (g/d) \| -3 \| -3 \| -0.7 (0.63) \| -3 \| -3 \| -0.3 (0.54) \| \| Polunsat fat (g/d) \| -1 \| -1 \| -0.7 (0.37) \| -1 \| 0 \| -0.7 (0.3) \| \| Monounsat fat (g/d) \| -2 \| -1 \| -1.1** (0.65) \| -2 \| -1 \| -1.4 (0.48) \|   *Adjusted difference = adjusted for age, gender and mean between baseline and follow up  ** = significant difference between intervention and control region |
| **Secondary Outcomes SEP** | NA |
| **Primary Outcomes age/sex** | Mean change in diet after 5 years of intervention and adjusted difference* (SE) in change stratified by gender   \| Factor \| Male \| \| \| Female \| \| \| \| --- \| --- \| --- \| --- \| --- \| --- \| --- \| \|  \| Interv \| Control \| Adj. diff \| Interv \| Control \| Adj diff \| \| Energy intake (MJ/d) \| -0.8 \| -0.5 \| -0.2 (0.13) \| -0.5 \| -0.3 \| -0.2 (0.1) \| \| Fat intake (g/d) \| -7 \| -5 \| -2.3 (1.6) \| -5 \| -3 \| -2.5 (1.2) \| \| Sat fat (g/d) \| -4 \| -3 \| -0.6 (0.68) \| -3 \| -2 \| -0.3 (0.54) \| \| Polunsat fat (g/d) \| -1 \| -1 \| -0.6 (0.42) \| -1 \| 0 \| -0.7 (0.3) \| \| Monounsat fat (g/d) \| -2 \| -1 \| -1.1 (0.65) \| -2 \| 0 \| -1.4 (0.48) \|   *Adjusted difference = adjusted for age, education level and mean between baseline and follow up  Mean change in total fat intake, monounsaturated fat intake and energy intake significantly different between intervention and control in female only |
| **Secondary Outcomes age/sex** | NA |
| **Effect on inequalities** | **The mean change in energy intake is the primary outcome of interest. The lower SEG achieved a significantly lower energy intake than their control counterparts at the end of the intervention whereas the higher SEG did not. This intervention reduces inequalities.** |
| **Study authors conclusions** | Results indicate the intervention to be effective among groups of lower SEP but not as effective among groups of higher SEP – possibly due to the particular focus on lower SEP in some of the downstream interventions. |
| **Limitations** | The intervention was conducted in the whole community but not necessarily offered to the individuals in the intervention group as would be the case in a RCT. Limited budget meant only one control region was included. Cannot be ascertained that the observed net intervention effects are solely due to the intervention. |
| **Notes** |  |

***Product***

| Millett C, Laverty AA, Stylianou N, Bibbins-Domingo K, Pape UJ: **Impacts of a national strategy to reduce population salt intake in England: serial cross sectional study.** *PLoS ONE,* 2012, **7(1)**. | |
| --- | --- |
| **Funder(s)** | Higher education Funding Council for England and the National Institute for Health Research. |
| **“P”** | Product |
| **Aim(s)** | To assess the impact of the national strategy in the UK to reduce salt intake (including salt reformulation and increasing public awareness) on population intake and potential inequalities using national survey data from the health survey for England (HSE). |
| **Setting** | UK |
| **Recruitment and sample size** | Used data from NSE from 2003 – 2007. Respondents per year: 2003 = 1668, 2004 = 2840, 2005 = 4643, 2006 = 8844, 2007 = 4269) |
| **Study design** | Observational study |
| **Intervention(s) analysed** | Salt reformulation, food labelling, increasing public awareness |
| **Nutrient** | Salt |
| **Methods/**  **Intervention details** | NSE = annual survey of people living in private households and is the primary mechanism for monitoring health in England. 2 stage stratified sampling process is employed to obtain an independent and nationally representative sample each year. The core sample is boosted by sampling from groups of interest in some years e.g. ethnic minorities in 2004. Nurse visits respondents over 16 years of age and takes anthropometric measurements and blood and urine samples. % of respondents who provided a urine sample (2003-2007) was 9, 28.1, 34.9, 41.3 and 29.7. |
| **Length of follow up** | NA |
| **SEP measurement** | Social class non manual/manual |
| **Primary Outcomes SEP**  Trends in geometric mean (95% CI) daily salt intake (g/d)*   \| Variable \| 2003 \| \| 2004 \| \| 2005 \| \| 2006 \| \| 2007 \| \| \| --- \| --- \| --- \| --- \| --- \| --- \| --- \| --- \| --- \| --- \| --- \| \|  \| n \| Mean \| n \| Mean \| n \| Mean \| n \| Mean \| n \| Mean \| \| White \| 1573 \| 6.1 (5.9-6.4) \| 638 \| 4.81 (4.6-5.1) \| 4397 \| 4.74 (4.6-4.8) \| 8215 \| 4.63 (4.6-4.8) \| 3902 \| 4.47 (4.4-4.7) \| \| South Asian \| 50 \| 5.46 (4.6-6.5) \| 1245 \| 6.14 (5.9-6.4) \| 121 \| 6.15 (5.5-6.9) \| 320 \| 6.44 (6-6.9) \| 186 \| 5.58 (5.1-6.1) \| \| Black \| 26 \| 8.44 (7-10.1) \| 650 \| 6.89 (6.6-7.2) \| 45 \| 6.3 (5.1-7.7) \| 168 \| 6.52 (5.9-7.2) \| 90 \| 5.52 (4.8-6.4) \| \| Non manual \| 944 \| 5.04 (4.8-5.3) \| 1337 \| 5.91 (5.7-6.1) \| 2783 \| 4.52 (4.4-4.6) \| 5219 \| 4.42 (4.3-4.5) \| 2510 \| 4.32 (4.2-4.4) \| \| Manual \| 661 \| 5.61 (5.3-5.9) \| 1066 \| 6.02 (5.8-6.3) \| 1675 \| 5.24 (5.1-5.4) \| 3260 \| 5.15 (5-5.3) \| 1562 \| 4.87 (4.7-5) \|   *these values are from a spot urine test and do not reflect 24 hour intake  % change 2003-2007   \| White \| -14.2 \| \| --- \| --- \| \| South Asian \| +2.2 \| \| Black \| -34.6 \| \| Non manual \| -14.3 \| \| Manual \| -13.2 \|   Difference in reductions in salt intake between manual and non-manual workers = p=0.272. | |
| **Secondary Outcomes SEP**  Trends in salt intake from linear time regression models   \| variable \| Adjusted geometric mean in 2003 (g/d) \| Regression coefficient for slope (relative to reference category) \| Overall regression coefficient for slope \| 95% lower CI (log scale) \| 95% upper CI (log scale) \| Reduction in g/d/year \| P value for differences between groups* \| \| --- \| --- \| --- \| --- \| --- \| --- \| --- \| --- \| \| ETHNICITY \| \| \| \| \| \| \| \| \| White \| 5.21 \| Ref \| -0.034 \| -0.043 \| -0.025 \| -0.173 \| <0.001 \| \| South Asian \| 5.14 \| 0.016 \| -0.01 \| -0.016 \| 0.047 \| -0.051 \| 0.333 \| \| Black \| 8.09 \| -0.016 \| -0.05 \| -0.057 \| 0.026 \| -0.394 \| 0.465 \| \| SOCIAL CLASS \| \| \| \| \| \| \| \| \| Non manual \| 5.01 \| Ref \| -0.04 \| -0.048 \| -0.026 \| -0.197 \| <0.001 \| \| Manual \| 5.6 \| 0.009 \| -0.03 \| -0.03 \| 0.01 \| -0.165 \| 0.272 \|   *P values for reference categories reflect differences of the trend from zero, while all others reflect differences from the reference group trend | |
| **Primary Outcomes age/sex**  Trends in geometric mean (95% CI) daily salt intake (g/d)*   \| Variable \| 2003 \| \| 2004 \| \| 2005 \| \| 2006 \| \| 2007 \| \| \| --- \| --- \| --- \| --- \| --- \| --- \| --- \| --- \| --- \| --- \| --- \| \|  \| n \| Mean \| n \| Mean \| n \| Mean \| n \| Mean \| n \| Mean \| \| 16-34 \| 388 \| 6.56 (6.1-7) \| 971 \| 7.13 (6.9-7.4) \| 1059 \| 6.19 (5.9-6.4) \| 1856 \| 6.17 (6-6.4) \| 950 \| 5.94 (5.7-6.2) \| \| 35-54 \| 650 \| 5.26 (5-5.5) \| 1224 \| 7.13 (6.9-7.4) \| 1736 \| 5.05 (4.9-5.2) \| 3331 \| 4.91 (4.8-5) \| 1571 \| 4.77 (4.6-4.9) \| \| 54-74 \| 499 \| 4.72 (4.5-5) \| 551 \| 5.94 (5.7-6.2) \| 1445 \| 3.96 (3.8-4.1) \| 2844 \| 4 (3.9-4.1) \| 1357 \| 3.84 (3.7-4) \| \| 75+ \| 131 \| 4.38 (4-4.8) \| 94 \| 4.79 (4.5-5.1) \| 403 \| 3.9 (3.7-4.1) \| 813 \| 3.96 (3.8-4.1) \| 391 \| 3.6 (3.4-3.8) \| \| Men \| 735 \| 6.1 (5.9-6.4) \| 1279 \| 4.05 (3.6-4.6) \| 2085 \| 5.5 (5.4-5.7) \| 3977 \| 5.41 (5.3-5.5) \| 1926 \| 5.16 (5-5.3) \| \| Women \| 933 \| 4.73 (4.5-4.9) \| 1561 \| 5.7 (5.5-5.9) \| 2558 \| 4.36 (4.1-4.4) \| 4667 \| 4.2 (4.2-4.3) \| 2343 \| 4.12 (4-4.2) \|   *these values are from a spot urine test and do not reflect 24 hour intake  % change 2003-2007   \| 16-34 \| -9.5 \| \| --- \| --- \| \| 35-54 \| -9.3 \| \| 54-74 \| -18.6 \| \| 75+ \| -17.8 \| \| Men \| -15.4 \| \| Women \| -12.9 \| | |
| **Secondary Outcomes age/sex**  Trends in salt intake from linear time regression models   \| variable \| Adjusted geometric mean in 2003 (g/d) \| Regression coefficient for slope (relative to reference category) \| Overall regression coefficient for slope \| 95% lower CI (log scale) \| 95% upper CI (log scale) \| Reduction in g/d/year \| P value for differences between groups* \| \| --- \| --- \| --- \| --- \| --- \| --- \| --- \| --- \| \| AGE \| \| \| \| \| \| \| \| \| 16-34 \| 6.77 \| ref \| -0.036 \| -0.055 \| -0.018 \| -0.242 \| <0.001 \| \| 35-54 \| 5.24 \| 0.009 \| -0.03 \| -0.013 \| 0.031 \| -0.155 \| 0.418 \| \| 55-74 \| 4.74 \| -0.003 \| -0.04 \| -0.026 \| 0.02 \| -0.186 \| 0.788 \| \| 75+ \| 4.36 \| 0.002 \| -0.04 \| -0.032 \| 0.036 \| -0.171 \| 0.903 \| \| SEX \| \| \| \| \| \| \| \| \| Men \| 6.05 \| Ref \| -0.038 \| -0.05 \| -0.018 \| -0.226 \| <0.001 \| \| Women \| 4.66 \| 0.009 \| -0.03 \| -0.007 \| 0.025 \| -0.138 \| 0.265 \|   *P values for reference categories reflect differences of the trend from zero, while all others reflect differences from the reference group trend | |
| **Effect on inequalities** | **The change in salt intake from 2003-2007 is the primary outcome of interest. There was no significant difference between the high and low SEG for change in intake. This intervention had no impact on inequalities.** |
| **Study authors conclusions** | Policy makers in other countries should consider following the UK approach of designing and implementing salt reduction strategies in ways to bring disproportionate benefit to disadvantaged communities who experience higher rates of cardiovascular disease. The findings suggest that persons from manual occupational groups and ethnic minorities achieved similar reductions in salt intake as non-manual and white ethnic groups. It is important that monitoring strategies for salt reduction strategies currently being developed by the World Health Organisation consider equity impacts explicitly in their evaluation frameworks |
| **Limitations** | Comparing differences over time using cross sectional surveys may introduce bias as there may be systematic differences between respondents sampled in different years. Salt intake derived from spot tests rather than “gold standard” 24 hour urine samples, urine sample was optional and there may have been some self-selection therefore as only a relative few participants submitted samples. They were unable to isolate the reductions resulting from other primary and secondary interventions. |
| **Notes** |  |

***Promotion***

| Capacci S, Mazzocchi M: **Five-a-day, a price to pay: An evaluation of the UK program impact accounting for market forces.** *Journal of Health Economics* 2011, **30:**87-98 | |
| --- | --- |
| **Funder(s)** | European Union Seventh Framework Programme. |
| **“P”** | Promotion |
| **Aim(s)** | To evaluate the UK 5-a-day campaign that launched at a national level in March 2003 considering economic factors. |
| **Setting** | UK |
| **Recruitment and sample size** | Used micro-level household data from the Expenditure and Food Survey (EFS) between 2002/2003 and 2005/2006. The EFS is nationally representative sample. The number of sample observations collected was: 2002-2003 = 6917, 2003-2004 = 7029, 2004-2005 = 6779 and 2005-2006 = 6776. |
| **Study design** | Modelling study |
| **Intervention(s) analysed** | Health information campaign (5 a day) |
| **Nutrient** | Fruit and vegetables |
| **Methods/**  **Intervention details** | The authors adopt a Quadratic Almost Ideal Demand System (QAIDS) specification with demographic scaling. They used the data set and retail prices of fruit and vegetables over the same period of time to make estimations of prices from unit values. To estimate the impact of the policy, the authors estimated the QUAIDS in each period, and generated expenditure shares over time using the estimated QUAIDS coefficients and explanatory variables over the same period. |
| **Length of follow up** | The study examined 4 years of data |
| **SEC measurement** | Household income quartiles, quartile 1 = lowest SEP, quartile 4 = highest SEP |
| **Primary Outcomes SEP**  Policy impact portions per capita per day (bootstrapped SE) by SEP*   \| Income quartile \| 2003 \| 2004 \| 2005 \| \| --- \| --- \| --- \| --- \| \| Fruit \| \| \| \| \| 1^st^ \| -0.03 (0.08) \| -0.02 (0.07) \| **0.22 (0.08)** \| \| 2^nd^ \| 0.13 (0.12) \| 0.05 (0.12) \| **0.28 (0.11)** \| \| 3^rd^ \| 0.22 (0.15) \| 0.19 (0.12) \| **0.45 (0.15)** \| \| 4^th^ \| -0.17 (0.17) \| -0.12 (0.15) \| 0.08 (0.2) \| \| Total \| -0.01 (0.05) \| -0.01 (0.05) \| **0.18 (0.05)** \| \| Vegetables \| \| \| \| \| 1^st^ \| -0.04 (0.06) \| 0.06 (0.06) \| **0.24 (0.07)** \| \| 2^nd^ \| -0.03 (0.1) \| 0.03 (0.1) \| 0.13 (0.08) \| \| 3^rd^ \| 0.16 (0.1) \| **0.19 (0.09)** \| **0.27 (0.11)** \| \| 4^th^ \| 0.04 (0.11) \| 0.08 (0.1) \| 0.18 (0.12) \| \| Total \| -0.04 (0.04) \| 0.05 (0.03) \| **0.13 (0.03)** \| \| Fruit and vegetables \| \| \| \| \| 1^st^ \| -0.07 (0.12) \| 0.04 (0.11) \| **0.44 (0.12)** \| \| 2^nd^ \| 0.1 (0.18) \| 0.07 (0.18) \| **0.41 (0.15)** \| \| 3^rd^ \| 0.37 (0.21) \| 0.38 (0.17) \| **0.7 (0.23)** \| \| 4^th^ \| -0.12 (0.22) \| -0.04 (0.2) \| 0.25 (0.26) \| \| Total \| -0.05 (0.07) \| 0.03 (0.07) \| **0.31 (0.07)** \|   *values in bold indicate 5% significance level | |
| **Secondary Outcomes SEP** | Fruit and vegetable intakes (portions per capita) by income based on data from the EFS   \| Quartile \| 2002/2003 \| 2003/2004 \| 2004/2005 \| 2005/2006 \| \| --- \| --- \| --- \| --- \| --- \| \| 1^st^ \| 2.88 \| 2.81 \| 2.81 \| 3.25 \| \| 2^nd^ \| 3.43 \| 3.52 \| 3.45 \| 3.66 \| \| 3^rd^ \| 3.98 \| 3.99 \| 3.95 \| 4.3 \| \| 4^th^ \| 4.86 \| 4.7 \| 4.72 \| 4.96 \| \| Total \| 3.75 \| 3.69 \| 3.7 \| 3.98 \| |
| **Primary Outcomes age/sex** | NA |
| **Secondary Outcomes age/sex** | NA |
| **Effect on inequalities** | **The change in consumption of fruit and vegetables in 2005 is the primary outcome of interest (this is the data point that the sample have had the most experience with the intervention). The lowest SEG significantly increased their fruit and vegetable intake by 0.44 portions while the high SEG increased their intake by 0.25 portions but this failed to reach significance. This reduces inequalities.** |
| **Study authors conclusions** | The average impact of the campaign can be estimated at about +0.3 portions of fruit and vegetables per person per day, a figure which is slightly higher than the observed increase of 0.2 portions. While higher prices have moderated the positive outcomes of the policy, their adverse effect has been mitigated by declining price elasticities, a behavioural response which might be in itself a consequence of the 5-a-day program. The estimated consumption response varies by income quartile. No significant effect is registered for the highest income group, against significant increases for all other quartiles. The estimated impact in 2005/2006 is around 0.4 portions for the two lowest quartiles against an observed increase of around 0.3 portions compared to the reference year 2002/2003. The impact is even higher for the third quartile, 0.7 portions versus an observed increase of 0.3 portions. The lack of response for the highest income quartile may be explained by the fact that this group has already met the 5-a-day target (4.96 portions in 2005/2006), while the higher response for the third quartile compared to the lower quartiles is consistent with the relatively higher income elasticity in the demand for health |
| **Limitations** | This is now out of date - The most recent aggregate figures from DEFRA (2010) indicate a new drop in purchases for 2007 and 2008, with an estimate intake down again to an average of less than 3.8 portions per day. Over these 2 years, the real price of vegetables has gone up by 5% compared to other foods and 10% compared to non-foods, while the rise in fruit prices (+9% in nominal terms) has been lower than the overall rise in food prices (+14%). Modelling study – limitations by methodology. |
| **Notes** |  |

| Dallongeville J, Dauchet L, Mouzon Od, Réquillart V, Soler L-G: **Increasing fruit and vegetable consumption: a cost-effectiveness analysis of public policies.** *The European Journal of Public Health* 2011, **21:**69-73. | |
| --- | --- |
| **Funder(s)** | Does not specify |
| **“P”** | Promotion |
| **Setting** | France |
| **Aim(s)** | To quantify cost-effectiveness of policies aimed at increasing F&V consumption by examining both an optimistic and pessimistic consideration of an information campaign. |
| **Recruitment and sample size** | Used fruit and vegetable consumption data from the Individual and National Study on Food Consumption (national population survey in France - INCA). 2,624adults and 1,455 children completed INCA. |
| **Study design** | Modelling study |
| **Intervention(s) analysed** | Health information campaign |
| **Nutrient** | Fruit and vegetables |
| **Methods/ intervention details** | Based the economic model on previous similar French studies. To estimate subsequent health benefits of consumption data was used from the world cancer research fund and some published meta-analyses. A €10M information campaign was examined as this corresponds to the annual amount spent by public authorities and producers’ associations to promote F & V consumption in France. This has a pessimistic and an optimistic scenario as based on previous literature concerning the US and applying this to France. Monte Carlo simulations were used drawing 10 million times a 19-uplet of parameters. |
| **Length of follow up** | NA |
| **SEP measurement** | Decile of income (first decile of income is the lowest SEG) |
| **Primary Outcomes SEP** | \| Variable \| Pessimistic \| Optimistic \| \| --- \| --- \| --- \| \| **Consumption variation (g/day)** \|  \|  \| \| All deciles \| 0.4 (0.2-0.6) \| 4 (2.2-6.4) \| \| First decile \| 0.3 (-0.1-0.5) \| 2.6 (-0.8-5.3) \| \| Other deciles \| 0.4 (0.2-0.7) \| 4.1 (2-6.9) \|   Estimated mean (CI) change in consumption levels of F & V |
| **Secondary Outcomes SEP** | Estimated mean (CI) change in Deaths avoided (DA) and Life years gained (LYS)   \| Variable \| Pessimistic \| Optimistic \| \| --- \| --- \| --- \| \| **Number of Deaths avoided (DA)** \|  \|  \| \| All deciles \| 30 (15-51) \| 298 (149-507) \| \| First decile \| 4 (-1-8) \| 37 (-12-81) \| \| Other deciles \| 26 (11-47) \| 262 (114-427) \| \| **Number of Life Years Gained (LYS)** \|  \|  \| \| All deciles \| 414 (203-710) \| 4126 (2022-7077) \| \| First decile \| 49 (-16-110) \| 492 (-156-1092) \| \| Other deciles \| 364 (156-663) \| 3633 (1557-6611) \| |
| **Primary Outcomes age/sex** | NA |
| **Secondary Outcomes age/sex** | NA |
| **Effect on inequalities** | **The change in fruit and vegetable consumption is the primary outcome of interest. As the confidence intervals overlap for the change in consumption between the highest and lowest SEG, this intervention had no impact on inequalities.** |
| **Study authors conclusions** | Owing to their lower cost, information campaigns are more cost-effective, despite lower DA than VAT reduction. |
| **Limitations** | Limitations associated with modelling studies. Inconsistent data sets used – seem to pluck relevant information from different places and incorporate it. The meta-analyses that the researchers relied upon were subject to criticisms concerning the accuracy of food intake assessment, the quality of event ascertainment, measurement of confounders and publication bias. |
| **Notes** |  |

| Estaquio C, Druesne-Pecollo N, Latino-Martel P, Dauchet L, Hercberg S, Bertrais S: **Socioeconomic differences in fruit and vegetable consumption among middle-aged French adults: adherence to the 5 A Day recommendation.** *Journal of the American Dietetic Association*, **108:**2021-2030 | |
| --- | --- |
| **Funder(s)** | States that the first author was supported in part by the Foundation Louis Bonduelle, France. This acts internationally with the aim of changing eating habits in a sustainable manner, by providing everyone with the means of bringing vegetables into their daily life. |
| **“P”** | Promotion |
| **Aim(s)** | To investigate relationships of socioeconomic, demographic and behaviour factors with both quantity and variety of fruit and vegetable consumption in a sample of middle-aged French subjects. |
| **Setting** | France |
| **Recruitment and sample size** | Used data from the Supplementation en Vitamines Minereaux et AntioXydants (SU.VI.MAX) 8 year cohort = randomised RCT that examined the efficacy of daily supplementation of antioxidant vitamins and minerals that had 13, 017 subjects aged 45-62 (5141 men, 7876 women). This study used 4282 people (2373 men, 1909 women). |
| **Study design** | Retrospective cohort study |
| **Intervention(s) analysed** | Health information campaign (5 A Day) |
| **Nutrient** | Fruit and vegetables |
| **Methods/**  **Intervention details** | For this study, only those who competed at least six 24 hour dietary records during the first 2 years of follow up who had all available socioeconomic data and completed records in both the autumn-winter and spring-summer to control for seasonality. Logistic regression carried out to calculate odds ratio (OR) of meeting 5 a day recommendations. Calculated the variety of fruit and vegetables consumed. Each variety was given a score of 1. Mixed dishes were given a score of 2 when the recipe contained at least two types of fruit/vegetables. |
| **Length of follow up** | The cohort that the data is taken from lasted eight years |
| **SEP measurement** | Education level, occupation (split by gender and not analysed as a whole) |
| **Primary Outcomes SEP** | % achieving >= 5 servings of fruit and vegetables per day SEP split by gender:   \|  \| men \| \| women \| \| \| --- \| --- \| --- \| --- \| --- \| \| characteristic \| % \| OR (CI) \| % \| OR (CI) \| \| **education** \|  \|  \|  \|  \| \| University or equivalent \| 58.1 \| 1 (referent) \| 56.7 \| 1 (referent) \| \| Secondary school \| 52.6 \| 0.74 (0.59-0.92) \| 53.2 \| 0.88 (0.69-1.11) \| \| Elementary school \| 50.4 \| 0.7 (0.54-0.92) \| 46.4 \| 0.65 (0.48-0.88) \| \| Trend (p value) \| 0.007 \| 0.0008 \| 0.005 \| 0.009 \| \| **Occupation** \|  \|  \|  \|  \| \| Managerial \| 55.6 \| 1 (referent) \| 54.9 \| 1 (referent) \| \| Intermediate professions, employees \| 55 \| 1.13 (0.91-1.4) \| 52.2 \| 0.96 (0.73-1.27) \| \| Farmers, self employed \| 46.5 \| 0.77 (0.51-1.18) \| 58.6 \| 1.34 (0.78-2.3) \| \| Manual workers \| 47.3 \| 0.86 (0.58-1.28) \| 53.2 \| 1.36 (0.68-2.72) \| \| Trend \| 0.06 \| 0.6 \| 0.57 \| 0.66 \| \| **Household location** \|  \|  \|  \|  \| \| Urban \| 50.3 \| 1 (referent) \| 51.6 \| 1 (referent) \| \| Rural \| 55.1 \| 1.31 (0.97-1.77) \| 53.3 \| 1.17 (0.89-1.55) \| \| P \| 0.08 \| 0.07 \| 0.59 \| 0.36 \|   *Significant effect of education level – more educated = more f & v consumed |
| **Secondary Outcomes SEC** | Variety score for fruit and vegetables for SEG split by gender:   \|  \| men \| \| women \| \| \| --- \| --- \| --- \| --- \| --- \| \| characteristic \| fruit \| vegetables \| fruit \| vegetables \| \| **education** \|  \|  \|  \|  \| \| University or equivalent \| 2.28 \| 2.02 \| 2.34 \| 2.1 \| \| Secondary school \| 2.13 \| 1.88 \| 2.53 \| 2,07 \| \| Elementary school \| 1.96 \| 1.88 \| 2.37 \| 1.96 \| \| Trend (p value) \| 0.0002 \| 0.002 \| 0.78 \| 0.002 \| \| **Occupation** \|  \|  \|  \|  \| \| Managerial \| 2.16 \| 1.93 \| 2.43 \| 2.1 \| \| Intermediate professions, employees \| 2.19 \| 1.96 \| 2.43 \| 2.07 \| \| Farmers, self employed \| 1.74 \| 1.82 \| 2.43 \| 1.99 \| \| Manual workers \| 1.96 \| 1.88 \| 2.19 \| 1.99 \| \| Trend \| 0.005 \| 0.19 \| 0.71 \| 0.63 \| \| **Household location** \|  \|  \|  \|  \| \| Urban \| 2.16 \| 1.93 \| 2.37 \| 2.07 \| \| Rural \| 2.13 \| 1.93 \| 2.43 \| 2.07 \| \| P \| 0.62 \| 0.66 \| 0.53 \| 0.98 \| |
| **Primary Outcomes age/sex** | For sex, see previous table. >= 5 servings of fruit and vegetables per day by age split by gender   \|  \| men \| \| women \| \| \| --- \| --- \| --- \| --- \| --- \| \| Age \| % \| OR (CI) \| % \| OR (CI) \| \| 45-50 \| 49.2 \| 1 (referent) \| 48.8 \| 1 (referent) \| \| **50-55** \| 53.7 \| 1.26 (1.02-1.56) \| 52.7 \| 1.19 (0.95-1.5) \| \| 55+ \| 60.5 \| 1.75 (1.41-2.16) \| 60.7 \| 1.75 (1.37-2.23) \| \| Trend \| <0.0001 \| <0.0001 \| 0.0001 \| <0.0001 \| |
| **Secondary Outcomes age/sex** | For sex, see previous table. Variety score for fruit and vegetables for age split by gender   \|  \| men \| \| women \| \| \| --- \| --- \| --- \| --- \| --- \| \| Age \| fruit \| vegetables \| fruit \| vegetables \| \| 45-50 \| 2.13 \| 1.88 \| 2.43 \| 1.99 \| \| **50-55** \| 2.1 \| 1.93 \| 2.43 \| 2.1 \| \| 55+ \| 2.16 \| 2.02 \| 2.43 \| 2.13 \| \| Trend \| 0.77 \| <0.0001 \| 0.94 \| 0.001 \| |
| **Effect on inequalities** | **The % of males consuming more than 5 portions of fruit and vegetables per day is the primary outcome of interest. The odds of the least educated men are significantly lower for consuming five a day than for the most educated men. This therefore widens inequalities.** |
| **Study authors conclusions** | Higher socioeconomic groups also spent more to eat fruit and vegetables. This suggests that price intervention may be a valuable strategy for increasing the proportion of subjects who attain the recommended levels, especially in low-income socioeconomic groups. A reduction in the price of fruit and vegetables, which can be achieved by means of public policies, could lead to an increase in fruit and vegetable intake |
| **Limitations** | Making the assumption that the rates of fruit consumed is due to 5 a day. These could be considered baseline characteristics of a population, and could be attributable to anything else. Does not examine SEG as a whole, but split by gender – would have been interesting to have seen as a whole. More than half of the subjects achieved the five servings of fruit and vegetables per day – seems particularly high – indicative of French population? Caution when making comparisons to other populations. The subjects were participants in a nutritional intervention study therefore they might have had a healthier lifestyle and diet pattern. Those with higher levels of education may have over reported their consumption. |
| **Notes** | They also examined how money each of the groups spent on fruit and vegetables per day |

| Stables GJ, Subar AF, Patterson BH, Dodd K, Heimendinger J, Van Duyn MAS, Nebeling L: **Changes in vegetable and fruit consumption and awareness among US adults: Results of the 1991 and 1997 5 A Day for Better Health Program surveys.** *Journal of the American Dietetic Association* 2002, **102:**809-817. | |
| --- | --- |
| **Funder(s)** | Not stated |
| **“P”** | Promotion |
| **Aim(s)** | To characterise population based changes in fruit and vegetables consumption and 5 a day awareness from 1991 to 1997, first by comparing intakes at each point in time (unadjusted) and then by controlling for demographic and other factors over time. |
| **Setting** | USA |
| **Recruitment and sample size** | Baseline survey in 1991 = 2837 people, with African Americans and Latinos over sampled to provide reliable population based estimates (n = 1031). Follow up survey in 1997 had 2602 people – this had 2 strata: a national sample in which all respondents were selected without regard to race and a sample of individuals with telephone exchanges from communities in which 15% or more of the households were African-American or Latino to maintain comparability with the baseline survey. Excluded 142 respondents for not fully completing questionnaire. |
| **Study design** | Random digit dial survey |
| **Intervention(s) analysed** | Health information campaign (5 A Day) |
| **Nutrient** | Fruit and vegetables |
| **Methods/**  **Intervention details** | Respondents were asked identical questions in the surveys – validated questionnaire. 7 item Fruit Frequency Questionnaire (FFQ) included asking them how often they had fruit juices (100% and other fruit juices), green salad, potatoes (including fries and baked) and how many servings of fruit apart from fruit juice. The two samples were weighted to the general population using 1991 census data and 1997 current general population survey to give weighted means. Used regression equations to get adjusted means to control for demographic changes in the population between 1991 and 1997. |
| **Length of follow up** | 6 years |
| **SEP measurement** | Education, ethnicity, poverty index ratio (composite measure of income and size of household) |
| **Primary Outcomes SEP** | Weighted and regression adjusted mean (standard error) servings of fruit and vegetables intake by SEP   \|  \| Weighted means \| \| \| Model-adjusted means \| \| \| \| --- \| --- \| --- \| --- \| --- \| --- \| --- \| \| Characteristic \| 1991 \| 1997 \| P value \| 1991 \| 1997 \| P value \| \| **Race** \|  \|  \|  \|  \|  \|  \| \| White \| 3.74 (0.05) \| 3.97 (0.08) \| 0.012 \| 3.75 (0.05) \| 3.88 (0.07) \| 0.125 \| \| African- American \| 4 (0.14) \| 3.81 (0.15) \| 0.342 \| 4.01 (0.13) \| 3.79 (0.14) \| 0.254 \| \| Hispanic \| 3.42 (0.12) \| 3.93 (0.2) \| 0.032 \| 3.55 (0.12) \| 4.04 (0.22) \| 0.043 \| \| **Education** \|  \|  \|  \|  \|  \|  \| \| <high school \| 3.51 (0.13) \| 3.55 (0.14) \| 0.832 \| 3.53 (0.13) \| 3.55 (0.14) \| 0.892 \| \| High school \| 3.56 (0.08) \| 3.69 (0.11) \| 0.329 \| 3.58 (0.08) \| 3.74 (0.11) \| 0.23 \| \| >high school \| 3.97 (0.06) \| 4.22 (0.08) \| 0.014 \| 3.98 (0.06) \| 4.1 (0.08) \| 0.23 \| \| **Poverty index ratio** \|  \|  \|  \|  \|  \|  \| \| <130% \| 3.5 (0.12) \| 3.85 (0.15) \| 0.018 \| 3.61 (0.13) \| 3.9 (0.16) \| 0.122 \| \| 130%-300% \| 3.57 (0.07) \| 3.86 (0.11) \| 0.031 \| 3.63 (0.07) \| 3.82 (0.12) \| 0.122 \| \| >300% \| 3.98 (0.07) \| 4.07 (0.09) \| 0.472 \| 3.97 (0.07) \| 3.97 (0.08) \| 0.977 \| |
| **Secondary Outcomes SEP** | NA |
| **Primary Outcomes age/sex** | Weighted and regression adjusted mean (standard error) servings of fruit and vegetables intake by age   \|  \| Weighted means \| \| \| Model-adjusted means \| \| \| \| --- \| --- \| --- \| --- \| --- \| --- \| --- \| \| **Age** \| 1991 \| 1997 \| P value \| 1991 \| 1997 \| P value \| \| 18-34 \| 3.47 (0.8) \| 3.86 (0.11) \| 0.006 \| 3.54 (0.8) \| 3.84 (0.11) \| 0.033 \| \| 35-39 \| 3.77 (0.8) \| 3.89 (0.1) \| 0.383 \| 3.73 (0.8) \| 3.8 (0.11) \| 0.586 \| \| 50-64 \| 3.89 (0.12) \| 4.03 (0.16) \| 0.465 \| 3.94 (0.11) \| 3.86 (0.12) \| 0.62 \| \| >=65 \| 4.23 (0.12) \| 4.33 (0.13) \| 0.531 \| 4.29 (0.12) \| 4.3 (0.12) \| 0.934 \| |
| **Secondary Outcomes age/sex** | NA |
| **Effect on inequalities** | **The model adjusted mean change in fruit and vegetable consumption is the primary outcome of interest. Those scoring highest and lowest on the poverty index ratio failed to achieve a significant increase in consumption. This intervention had no impact on inequalities.** |
| **Study authors conclusions** | The public awareness of the 5 a day message was substantially higher in 1997 compared to 1991. This could serve as a model for other nutrition and physical activity messages. Dietary counselling aimed at African Americans, those less educated and the young is warranted |
| **Limitations** | Potatoes are not a vegetable – they are a starch, 3 of the 7 items of FFQ related to potatoes. Potatoes were included in the analysis, just not fried potatoes. Not the same people in the samples and cannot infer that this is a change over time as these are not the same people being sampled. Portion sizes were not asked. |
| **Notes** | They did not carry out an intervention. Basing these inferences on that the change is due to awareness increasing of the 5 a day message – this may not be the case. |

***Person***

| Brownson RC, Smith CA, Pratt M, Mack NE, Jackson-Thompson J, Dean CG, Dabney S, Wilkerson JC: **Preventing cardiovascular disease through community-based risk reduction: the Bootheel Heart Health Project.** *American Journal of Public Health* 1996, **86:**206-213 | |
| --- | --- |
| **Funder(s)** | The Centres for Disease Control and Prevention cooperative agreement. |
| **“P”** | Person |
| **Aim(s)** | The long term goal is to reduce CVD. The shorter term project objectives focused on reducing the major modifiable risk factors for CVD. This report concerns the 5 year evaluation of the prevalence of CVD risk factors addressed by the project. |
| **Setting** | Bootheel is a 6 county area in south eastern Missouri, USA, with high rates of CHD related mortality. |
| **Recruitment and sample size** | Recruited for a survey using random-digit dialling, they selected cross-sectional samples of free living adults in the 6 county region. |
| **Study design** | Cross-sectional surveys |
| **Intervention(s) analysed** | Health education |
| **Nutrient** | Multiple (not specified) |
| **Methods/**  **Intervention details** | Local leaders were identified through established agencies and word of mouth, giving rise to 17 ‘subcoalitions’ within the 6 county region. These allowed ro local tailored interventions. Coalitions were allowed to select their own priorities form a list of CVD related interventions. Examples of these are – annual heart healthy fitness festivals, high BP Sundays – ministers dedicated some of their sermons to heart health with congregation being screened at the church and only heart healthy foods provided there, local school healthy heart poster contests, a weekly newspaper column on heart health and environmental changes such as construction of more walking paths. Most frequently held events were walking clubs (n = 4000) and exercise classes (n = 2050), community events (n = 415), cholesterol screening (n = 70), cooking demonstrations (n = 60) and diabetes screening (n = 30). Conducted 2 surveys to evaluate projects progress – in 1990 at the start of the project, and 1994. |
| **Length of follow up** | 5 years |
| **SEP measurement** | Race (white and black) and educational level (less than high school = <HS, high school graduate = HS, some college/technical school graduate = >HS, college graduate or more = C+) |
| **Primary Outcomes SEP**  Prevalence of risk factors (95% CI) for CVD by SEG: 1990 (n = 1006) and 1994 (n = 1510)   \| Variable \| Consumes 5+ F & V/d \| \| Overweight \| \| Cholesterol checked in past 2 years \| \| \| --- \| --- \| --- \| --- \| --- \| --- \| --- \| \|  \| 1990 \| 1994 \| 1990 \| 1994 \| 1990 \| 1994 \| \| White \| 20.9 (±2.9) \| 20.7 (±2.4) (-1% change) \| 25.9 (±3.2) \| 30.3 (±2.8) (+17% change) \| 51.2 (±4.5) \| 51.8 (±3.4) \| \| Black \| 14.9 (±10.1) \| 19.1 (±6.1) (+28%) \| 42.6 (±14) \| 42.3 (±8.9)  (-1% change) \| 36.9 (±11.8) \| 52.7 (±7.3) \| \| <HS \| 14.4 (±4.2) \| 19 (±3.8) (32% change) \| 31.7 (±5.4) \| 34.7 (±5) (9% change) \| 46.1 (±5.7) \| 53.2 (±5.2) \| \| HS \| 22.2 (4.4) \| 19.2 (±3.4) \| 24.4 (±4.6) \| 30.7 (±4.1) \| 51 (±5.5) \| 50.8 (±4.5) \| \| >HS \| 22 (±8.1) \| 20.5 (±5.5) \| 27.4 (±8.7) \| 29.4 (±6.5) \| 50.8 (±9.6) \| 52.7 (±7.3) \| \| C+ \| 30.4 (±10.2) \| 30.7 (±8) (1% change) \| 20.1 (±8.8) \| 29 (±8.1) (44% change) \| 58.1 (±10.6) \| 61.6 (±8.8) \| | |
| **Secondary Outcomes SEP** | NA |
| **Primary Outcomes age/sex**  Prevalence of risk factors (95% CI) for CVD by age and gender: 1990 (n = 1006) and 1994 (n = 1510)   \| Variable \| Consumes 5+ F & V/d \| \| Overweight \| \| Cholesterol checked in past 2 years \| \| \| --- \| --- \| --- \| --- \| --- \| --- \| --- \| \|  \| 1990 \| 1994 \| 1990 \| 1994 \| 1990 \| 1994 \| \| 18-34 \| 17.7 (±5.2) \| 17.2 (±4) \| 18 (±5) \| 28.1 (±4.9) \| 34.6 (±6.5) \| 37.9 (±5.4) \| \| 35-54 \| 20.5 (±4.5) \| 18.4 (±3.5) \| 36.3 (±5.5) \| 34.1 (±4.5) \| 48.1 (±5.7) \| 53.5 (±4.7) \| \| 55+ \| 24.1 (±4.8) \| 26.3 (±4) \| 26.5 (±5) \| 32.6 (±4.4) \| 69.8 (±4.9) \| 69.1 (±4.7) \| \| Female \| 22.6 (±3.7) \| 25.5 (±3.1) \| 24 (±3.8) \| 30.6 (±3.3) \| 51.5 (±4.5) \| 52.8 (±3.1) \| \| Male \| 17.9 (±4.3) \| 15.3 (±3.2) \| 30 (±4.9) \| 32.4 (±4.3) \| 48.5 (±5.5) \| 53.8 (±8.4) \| | |
| **Secondary Outcomes age/sex** | NA |
| **Effect on inequalities** | **The change in % of people consuming 5 a day is the primary outcome of interest. After calculating this, it would appear to reduce inequalities, with the lowest SEP increasing their intake by 32% while the highest SEP only increased their consumption by 1%. However, we cannot infer that this is statistically significant as we do not have confidence values for the change in consumption as this was calculated.** |
| **Authors conclusions** | The decline in physical inactivity and increase in cholesterol screening shown in the Bootheel project suggest that a community-level reduction in CVD risk may be achievable through relatively low-cost interventions that combine educational efforts with environmental changes. However, because of limitations in the study design, further data are needed, including longer term measurements of CVD factors, morbidity, and mortality, as well as replication of similar projects in other underserved areas |
| **Limitations** | Study lacked true experimental design and comparison groups. Cannot account for the effect of national programs (e.g. the national high BP education program) on changes in CVD risk factors in local populations. Self-report. Used telephone interviews – may have some response bias due to lack of phone coverage among certain SEGs. Did not collect “in-person” clinical data such as blood pressure/cholesterol measurements. |
| **Notes** | Sample slightly underrepresented younger people, males and those with less education. Cholesterol checks have been included in data extraction – are these relevant? |

| Bürgi F, Niederer I, Schindler C, Bodenmann P, Marques-Vidal P, Kriemler S, Puder JJ: **Effect of a lifestyle intervention on adiposity and fitness in socially disadvantaged subgroups of preschoolers: A cluster-randomized trial (Ballabeina).** *Preventive Medicine* 2012, **54:**335-340. | |
| --- | --- |
| **Funder(s)** | The Swizz national science foundation and Health Promotion Switzerland. Additional funding came from a research award for interdisciplinary research from the university of Lausanne, A takeda research award, the Wyeth foundation for the health of children and adolescents, the Freie Akademische Gesellscaft and an unrestricted educational grant from Nestle |
| **“P”** | Person |
| **Aim(s)** | The authors conducted a previous study that did not have an effect on BMI and agility in children. This study had a high proportion of migrant children and children who had parents of low educational level (EL). Therefore they now wanted to carry out an intervention to determine the effectiveness on these two pre defined groups of high-risk children regarding its effects on adiposity and fitness. |
| **Setting** | 40 randomly selected public preschool classes in areas with a high migrant population from two different socio-cultural and linguistic regions in Switzerland. |
| **Recruitment and sample size** | Parents of children in the schools were contacted for consent. Overall 652 preschool children were included in the analysis (mean age = 5.2 (+-0.6) years, 72% migrant children, 38% children of low EL. |
| **Study design** | Cluster RCT |
| **Intervention(s) analysed** | Health education |
| **Nutrient** | Not specified |
| **Methods/**  **Intervention details** | Children did 4x45 mins physical activity lessons per week. In addition, there were 22 lessons in healthy nutrition, media use and sleep. Health foods were promoted for snacks and treats. Participation was mandatory for all children – irrespective of consent. Teachers were trained and supported by experts throughout. Parents took part in 3 interactive discussion evenings and children brought home information leaflets and brochures for the parents throughout. The environment in and around the preschool classes was adapted to promote physical activity, e.g. climbing bars etc. The control group did not receive any intervention. |
| **Length of follow up** | Intervention lasted one school year – pre and post tested. |
| **SEP measurement** | Children of migrant parents and children of parents who had one parent with no education beyond what was mandatory (9 years education) were low SEP. Children of non-migrant families and those with both parents having more than the mandatory amount of education were high SEP. |
| **Primary Outcomes SEC**  Mean BMI, % body fat and waist measurements pre and post intervention by SEG (standard deviations)  Non migrants (n = 173), migrants (n = 472), middle/high EL (n = 351), low EL (n = 213)   \|  \| Pre intervention \| \| Post intervention \| \| Adjusted change (CI) \| p \| \| --- \| --- \| --- \| --- \| --- \| --- \| --- \| \|  \| Intervention \| control \| Intervention \| Control \|  \|  \| \| BMI (kg/m²) \| \| \| \| \|  \|  \| \| Middle/high EL \| 15.6 (1.3) \| 15.8 (1.5) \| 15.5 (1.4) \| 15.8 (1.6) \| -0.11 (-0.29, 0.08) \| 0.253 \| \| Low EL \| 15.8 (1.6) \| 15.8 (1.5) \| 16 (1.6) \| 16 (1.9) \| 0.04 (-0.15, 0.23) \| 0.677 \| \| % BODY FAT \| \| \| \| \|  \|  \| \| Middle/high EL \| 23.3 (5.9) \| 23.2 (6.6) \| 22.4 (5.8) \| 23.6 (6.7) \| -1.29 (-2.33, -0.26) \| 0.015 \| \| Low EL \| 24.3 (6.9) \| 24.3 (6.9) \| 24.2 (6.9) \| 25 (7.3) \| -0.43 (-1.63, 0.77) \| 0.486 \| \| WAIST (cm) \| \| \| \| \|  \|  \| \| Middle/high EL \| 52.7 (3.9) \| 52.8 (4.2) \| 53.2 (3.9) \| 54.3 (4.9) \| -0.87 (-1.46, -0.27) \| 0.004 \| \| Low EL \| 53.1 (4.7) \| 53.2 (4.4) \| 53.5 (4.5) \| 54.7 (5.3) \| -1.1 (-2, -0.2) \| 0.017 \| | |
| **Secondary Outcomes SEP** | NA |
| **Primary Outcomes age/sex** | NA |
| **Secondary Outcomes age/sex** | NA |
| **Effect on inequalities** | **The change in mean BMI is the primary outcome of interest. The confidence intervals overlap for the change in mean BMI in the low SEP (-0.11 kg/m^2^) and the high SEP (+0.04 kg/m^2^). There is no significant impact on inequalities.** |
| **Study authors conclusions** | The intervention was similarly beneficial among pre-schoolers of migrant and non-migrant parents. However, children of low EL parents has a smaller benefit (i.e. had smaller intervention effect sizes) compared to children of high/middle EL parents, however this did not reach a statistical difference. |
| **Limitations** | The study was not powered enough to conduct subgroup analyses and interactions. Small sample size in children with low EL parents which may have given a higher than actual p value. Large difference in sample size of different SEG groups. |
| **Notes** |  |

| Carcaise-Edinboro P, McClish D, Kracen AC, Bowen D, Fries E: **Fruit and vegetable dietary behavior in response to a low-intensity dietary intervention: the rural physician cancer prevention project.** *Journal of Rural Health*, **24:**299-305. | |
| --- | --- |
| **Funder(s)** | National Cancer Institute |
| **“P”** | Person |
| **Aim(s)** | Changes in fruit and vegetable behaviour were evaluated to assess the effects of a low-intensity, physician-endorsed dietary intervention in a rural population |
| **Setting** | 3 physician practices in rural Virginia, USA. |
| **Recruitment and sample size** | 754 people initially, but only 623 people had at least one follow up and were subsequently included in the analysis – 37% African American (Af/Am); 65% women; mean age 48.5 years (19-72 years) |
| **Study design** | RCT |
| **Intervention(s) analysed** | Health information campaign (Five a Day) |
| **Nutrient** | Fruit and vegetables |
| **Methods/**  **intervention details** | Tailored feedback and self-help dietary intervention vs no intervention. Intervention materials were developed at a sixth grade literacy level. Baseline telephone call. Followed immediately with a fat and fibre behaviour-related questionnaire (FFB) (used to assess F & V intake) and dietary recommendations posted to the home in the intervention group. These participants were posted intervention booklets once a week for four weeks detailing advice on healthful eating practices such as: reading labels, eating more F & V and selecting lower fat options. They were then telephoned for follow up interviews at 1, 6 and 12 months. Control participants were followed up concurrently. |
| **Length of follow up** | 12 months |
| **SEP measurement** | Education (<HS = less than high school education, HS = high school education, >HS = more than high school education |
| **Primary Outcomes SEP** | Mean fruit and Vegetable intake score by SEG (F & V intake reverse scored in FFB. Score out of 3, 3 = less F/V intake, 1 = more F/V intake) (standard deviation)   \| F & V intake \| Baseline \| \| 1 month \| \| 6 months \| \| 12 months \| \| \| --- \| --- \| --- \| --- \| --- \| --- \| --- \| --- \| --- \| \|  \| Control \| Interv \| Control \| Interv \| Control \| Interv \| Control \| Interv \| \| <HS \| 2.02 (0.49) \| 2.01 (0.54) \| 2.02 (0.49) \| 1.79 (0.54) \| 2 (0.44) \| 1.74 (0.51) \| 1.82 (0.45) \| 1.74 (0.52) \| \| HS \| 2.06 (0.43) \| 1.99 (0.46) \| 1.99 (0.49) \| 1.79 (0.46) \| 2.01 (0.43) \| 1.85 (0.4) \| 1.96 (0.55) \| 1.88 (0.44) \| \| >HS \| 1.92 (0.47) \| 1.99 (0.5) \| 1.91 (0.47) \| 1.9 (0.47) \| 1.93 (0.46) \| 1.86 (0.43) \| 1.83 (0.44) \| 1.83 (0.45) \|   ANOVA = p values comparing intervention and control groups:   \| Education \| 1 month \| 6 months \| 12 months \| \| --- \| --- \| --- \| --- \| \| <HS \| <0.004 \| <0.002 \| 0.02 \| \| HS \| 0.002 \| 0.001 \| 0.15 \| \| >HS \| 0.02 \| 0.01 \| 0.68 \| |
| **Secondary Outcomes SEP** | NA |
| **Primary Outcomes age/sex**  Mean servings of fruit and vegetables per day by age(standard deviation)   \| F & V servings \| Baseline \| \| 1 month \| \| 6 months \| \| 12 months \| \| \| --- \| --- \| --- \| --- \| --- \| --- \| --- \| --- \| --- \| \|  \| Control \| Interv \| Control \| Interv \| Control \| Interv \| Control \| Interv \| \| <43 years \| 2.26 (1.54) \| 2.47 (1.64) \| 2.59 (1.47) \| 3.16 (1.4) \| 2.73 (2.17) \| 3.43 (2.17) \| 2.84 (1.65) \| 3.14 (1.58) \| \| 43-55 years \| 2.58 (1.7) \| 2.76 (1.8) \| 2.94 (1.51) \| 3.24 (1.78) \| 3.05 (1.56) \| 3.24 (1.72) \| 3.14 (1.55) \| 3 (1.62) \| \| 56+ years \| 2.76 (1.4) \| 2.77 (1.48) \| 2.96 (1.61) \| 3.78 (1.93) \| 3.21 (1.73) \| 3.72 (1.7) \| 3.17 (1.69) \| 3.33 (1.69) \|   ANOVA p value comparing intervention and control groups   \| Age \| 1 month \| 6 months \| 12 months \| \| --- \| --- \| --- \| --- \| \| <43 years \| 0.003 \| 0.023 \| 0.52 \| \| 43-55 years \| 0.2 \| 0.54 \| 0.25 \| \| 56+ years \| <0.001 \| 0.001 \| 0.13 \| | |
| **Secondary Outcomes age/sex** | NA |
| **Effect on inequalities** | **The change in fruit and vegetables intake score is the primary outcome of interest. The lower SEP after 12 months had a significantly lower score than the control group. This translates into a higher reported intake of fruit and vegetables. The higher SEP did not have a significant difference between intervention and control after 12 months. This intervention reduces inequalities.** |
| **Authors conclusions** | That low intensity physician endorsed dietary interventions can be initiated in a hard to reach rural, high minority population, however, moderating effects of age and education necessitate consideration in the interpretation of these results. |
| **Limitations** | Self-report bias/social desirability, Tailoring of intervention materials toward lower SEP |
| **Notes** |  |

| Connett JE, Stamler J: **Responses of black and white males to the special intervention program of the Multiple Risk Factor Intervention Trial.** *American Heart Journal* 1984, **108:**839-849 | |
| --- | --- |
| **Funder(s)** | Not stated |
| **“P”** | Person |
| **Aim(s)** | The aim of MRFIT was to test whether lowering elevated serum cholesterol and diastolic blood pressure and ceasing cigarette smoking would reduce coronary heart disease mortality The aim of this paper is to present findings regarding the similarities and differences between black and white MRFIT participants in their responses to the multi-factorial special intervention (SI) program. |
| **Setting** | US |
| **Recruitment and sample size** | MRFIT was a randomized, primary prevention trial, conducted at 22 US clinical centres from 1973 to 1982. 12866 men took part. |
| **Study design** | RCT |
| **Intervention(s) analysed** | Dietary counselling |
| **Nutrient** | Fat |
| **Methods/**  **Intervention details** | Participants went through 3 screening visits to assess eligibility. Men with one or more of risk factors (cigarette smoking, high blood pressure, and elevated serum cholesterol) were randomly assigned to the SI or usual care (UC) group and followed for 6 years. UC men were given information on risk factors, referred to their usual sources of care, and re-examined annually. SI participants received group and individual counselling on a fat-modified diet, a stepped-care drug treatment program for diastolic hypertension (after an initial attempt at blood pressure control by weight reduction, if indicated), and, for cigarette smokers, counselling aimed at cessation. SI men had risk factor assessments every 4 months and annual examinations that were generally identical to those given to UC men and that always included measurement of blood cholesterol concentration. Standardised 24 hour recall was used to report food intake. |
| **Length of follow up** | 6 years |
| **SEP measurement** | Race – black (low SEP) and white (high SEP). Authors state that the black men had on average, lower education level and income than the white men. |
| **Primary Outcomes SEP**  Mean value at 6^th^ annual visit and % changes in risk factors from baseline by study group and race:   \|  \| SI \| \| \| \| UC \| \| \| \| \| --- \| --- \| --- \| --- \| --- \| --- \| --- \| --- \| --- \| \|  \| White (n = 5338) \| \| Black (n = 416) \| \| White (n = 5227) \| \| Black (n = 411) \| \| \| Risk factor \| 6^th^ visit \| %change \| 6^th^ visit \| %change \| 6^th^ visit \| %change \| 6^th^ visit \| %change \| \| DBP (mmHg) \| 80.4 \| -11.4 \| 81.3 \| -13.6 \| 83.5 \| -7.9 \| 85.6 \| -8.1 \| \| SBP (mmHg) \| 121.4 \| -10.3 \| 122.3 \| -11.7 \| 126.5 \| -6.4 \| 129.4 \| -5.8 \| \| Serum chol (mg/dl) \| 235.8 \| -7.3 \| 231 \| -6 \| 240.5 \| -5.6 \| 237.3 \| -3.7 \| \| Plasma chol (mg/dl) \| 228.5 \| -6.4 \| 224.4 \| -6.5 \| 233.3 \| -4.5 \| 230.2 \| -4.3 \| \| Plasma HDL chol (mg/dl) \| 41.3 \| -2.6 \| 46 \| -6.5 \| 41.4 \| -2.6 \| 47.6 \| -4.8 \| \| Plasma LDL chol (mg/dl) \| 148.7 \| -8.6 \| 148.3 \| -8.5 \| 152.8 \| -6.4 \| 153.8 \| -5.4 \| \| Plasma triglycerides (mg/dl) \| 200.9 \| +1.7 \| 153.9 \| +7.2 \| 203 \| +3.3 \| 147.1 \| +4 \| \| Weight (lb) \| 187.7 \| -0.7 \| 192.3 \| -0.2 \| 190.1 \| +0.7 \| 190.1 \| +0.7 \| | |
| **Secondary Outcomes SEP**  Reported changes in nutrients and weight from baseline to sixth annual visit of black and white MRFIT males by study group:   \|  \| White \| \| \| Black \| \| \| \| --- \| --- \| --- \| --- \| --- \| --- \| --- \| \| Nutrient \| SU \| UC \| SI-UC \| SI \| UC \| SI-UC \| \| Calories \| -623 \| -254 \| -369 \| -569 \| -148 \| -421 \| \| Fat (%) \| -5.1 \| -0.7 \| -4.4 \| -5.4 \| +0.3 \| -5.8 \| \| Sat fat (%) \| -4.1 \| -0.6 \| -3.4 \| -3.8 \| -0.2 \| -3.7 \| \| Mono fat (%) \| -2.9 \| -0.4 \| -2.5 \| -2.8 \| +0.2 \| -3 \| \| Poly fat (%) \| +2 \| +0.3 \| +1.7 \| +1.2 \| +0.2 \| +1.1 \| \| Cholesterol (mg) \| -185 \| -35 \| -150 \| -191 \| -8 \| -184 \| \| Weight (% change) \| -0.57 \| +0.76 \| -1.33 \| -0.07 \| +0.67 \| -0.76 \| | |
| **Primary Outcomes age/sex** | NA |
| **Secondary Outcomes age/sex** | NA |
| **Effect on inequalities** | **The change in mean cholesterol is the primary outcome of interest. Since there are no measurement errors or confidence intervals concerning this information, only a comparison of the magnitude of change between the lowest and highest SEP can be made. White men reduced their serum cholesterol more than black men (-7.3% compared to 6% respectively). Therefore this appears to widen inequalities, but we cannot infer that this is a significant widening effect.** |
| **Study authors conclusions** | The MRFIT experience indicates ability to induce extensive changes in lifestyles (eating and smoking habits) of both black and white American males varying in educational and SEG and also achieve long term effective adherence to programs for the treatment and control of hypertension. |
| **Limitations** | Exclusion of women, used “at risk” man to take part – can we infer that these effects would be achievable in those who were not at risk and there would remain no differential effects? |
| **Notes** |  |

| Curtis PJ, Adamson AJ, Mathers JC: **Effects on nutrient intake of a family-based intervention to promote increased consumption of low-fat starchy foods through education, cooking skills and personalised goal setting: the Family Food and Health Project.** *British Journal of Nutrition*, **107:**1833-1844 | |
| --- | --- |
| **Funder(s)** | The Food Standards Agency |
| **“P”** | Person |
| **Aim(s)** | The Family Food and Health Project (FFHP) was designed to determine which combinations of key facilitators of dietary change (i.e. information based food-related activities, cooking skills or personalised goal setting) would affect the translation of a message to increase the intake of low-fat starchy foods into improvement in dietary behaviours and, in particular, in reductions in fat intake |
| **Setting** | Newcastle upon Tyne |
| **Recruitment and Sample size** | Data presented relates to individuals who took part in the FFHP (Family Food and Health Project) in 1998-2000. This is 169 families (589 individuals). 63% of families from more affluent groups |
| **Study design** | Randomised parallel-group design intervention |
| **Intervention(s) analysed** | Health education |
| **Nutrient** | Fat |
| **Methods/**  **intervention details** | 3 groups randomly assigned to 3 interventions. Approximately equal numbers in each and were deliberately contiguous for enumeration districts to avoid cross contamination. All interventions aimed to broaden range of low fat starchy foods consumed and to increase the portion sizes of these. At the beginning of the study, all participants received a selection of low fat starchy foods to trial at home and a recipe file with low fat starchy meal/snack ideas. Participants used 3 day food diaries to record dietary intake at 4 time points (baseline, 3, 6 and 18 months)  Intervention A = a cooking fayre in a local community centre. 2 hour educational session, included food related games and challenges and food tasting (56 families;189 individuals).  Intervention B = 4 cook and eat sessions each lasting 2 hours each over 6 weeks. Meal planning, food preparation. Families were also encouraged to try less familiar low fat starchy foods during these sessions (58 families; 207 individuals)  Intervention C = Families were offered intervention A+B+ personalised goal setting from dieticians based on stage of change model (55 families; 193 individuals). |
| **Length of follow up** | 18 months |
| **SEP measurement** | Quintile of relative SES assessed using the Townsend Deprivation Index. SES2 = least socially deprived, SES4 = most socially deprived |
| **Primary Outcomes SEP** | Mean dietary intakes of percentage of food energy (%FE) from fat and total carbohydrate at baseline, 3 and 6 months (averaged across all three interventions)   \| SES \| Baseline \| 3 months \| 6 months \| \| --- \| --- \| --- \| --- \| \| %FE from fat \| \| \| \| \| SES2 \| 36.7 \| 35.3 \| 36.3 \| \| SES4 \| 37.1 \| 34.8 \| 34.8* \| \| %FE from carbohydrate \| \| \| \| \| SES2 \| 48.3 \| 50.3 \| 48.9 \| \| SES4 \| 48.6 \| 50.2 \| 50.3** \|   *SES4 fat intake significantly lower that SES2 at 6 months (p = 0.01); **SES4 carbohydrate intake was significantly higher than SES2 (p = 0.02) |
| **Secondary Outcomes SEP** | NA |
| **Primary Outcomes age/sex** | NA |
| **Secondary Outcomes age/sex** | NA |
| **Effect on inequalities** | **The mean change in food energy from fat is the primary outcome of interest. At 6 months SES4 achieved a significantly lower energy consumed from fat than the higher SES2. This intervention reduces inequalities.** |
| **Study authors conclusions** | The most intensive low-fat starchy food intervention, which included education, cooking skills and goal setting components, was the most effective in reducing fat intake and improving diet quality in both the short and longer term. Irrespective of intervention approach, the secondary analysis of the present study suggested that individuals residing in households with greater socioeconomic deprivation responded more favourably to the intervention in the medium term than those from more affluent areas. |
| **Limitations** | Self-report, the dietary data from individuals exhibited some inter-dependency as the intervention was aimed at the family level in households. Fewer dropouts from intervention A than B and C which were more time demanding |
| **Notes** |  |

| Friel S, Kelleher C, Campbell P, Nolan G: **Evaluation of the Nutrition Education at Primary School (NEAPS) programme.** *Public Health Nutrition*, **2:**549-555 | |
| --- | --- |
| **Funder(s)** | The national nutrition surveillance centre which is grant funded, was asked to undertake the project evaluation which the report is based on. |
| **“P”** | Person |
| **Aim(s)** | To design and pilot suitable educational materials for Irish school children aged 8–10 years old in order to build awareness of the benefits of healthy eating and regular exercise, to induce positive behaviour changes towards healthy foods and to increase children’s knowledge about healthy eating, and to determine if such changes were dependent on location or school classification |
| **Setting** | 8 primary schools in the Republic of Ireland. 4 schools from the NW health board, 4 schools from the E health board. 3 control schools (2 in NW health board (1 urban)) and 1 from the E health board (urban) |
| **Recruitment and sample size** | 821 children (453 in intervention schools; 368 in control schools). 64% of the sample responded with pre and post-test diaries. A random selection of these (n = 187) were analysed for the purposes of the evaluation. |
| **Study design** | RCT |
| **Intervention(s) analysed** | Health education |
| **Nutrient** | Fruit and vegetables, fibre, fat |
| **Methods/**  **intervention details** | Adapting an already existing dietary programme which has been used in the US. Programme materials used cartoon extra-terrestrial characters called “Hearty Heart and friends (based on the American hearty heart model). Consisted of lesson plans, activity worksheets for pupils, a home team pack to involve parents and food diaries. A ten week comparative quazi-experimental design was used in intervention schools receiving 20x30 minute sessions using a cross-curriculum approach. Participating teachers were trained how to teach the children about healthy eating. Baseline survey and initial 5 day food diaries were completed in both intervention and control schools. These were repeated post intervention. |
| **Length of follow up** | No follow up |
| **SEP measurement** | Location of the school. 4 schools from the NW health board – 3 were rural, 1 was urban. 2 schools from the E health board – all were urban and sited mainly in economically disadvantaged areas. |
| **Primary Outcomes SEP** | % of children consuming food items at different frequencies pre and post interventions. These are rounded averages and therefore do not add to 100.   \|  \| \| Intervention schools \| \| \| \| \| \| \| \| \| Control schools \| \| \| \| \| \| \| \| \| \| --- \| --- \| --- \| --- \| --- \| --- \| --- \| --- \| --- \| --- \| --- \| --- \| --- \| --- \| --- \| --- \| --- \| --- \| --- \| --- \| \| Number of servings \| \| All (n = 133) \| \| \| Urban (n = 86) \| \| \| Rural (n = 47) \| \| \| \| All (n = 54) \| \| \| Urban (n = 43) \| \| \| Rural (n = 11) \| \| \| DAIRY PRODUCTS \| \| \| \| \| \| \| \| \| \| \| \| \| \| \| \| \| \| \| \| \|  \| \| pre \| post \| \| pre \| post \| \| pre \| Post \| \| \| pre \| post \| \| pre \| post \| \| pre \| post \| \| <1 \| \| 18 \| 16 \| \| 13 \| 19 \| \| 28 \| 11 \| \| \| 39 \| 35 \| \| 39 \| 37 \| \| 26 \| 27 \| \| 1 \| \| 38 \| 36 \| \| 48 \| 39 \| \| 19 \| 30 \| \| \| 41 \| 37 \| \| 39 \| 37 \| \| 45 \| 36 \| \| 2 \| \| 23 \| 30 \| \| 16 \| 29 \| \| 34 \| 32 \| \| \| 15 \| 18 \| \| 14 \| 16 \| \| 18 \| 27 \| \| 3 \| \| 7 \| 5 \| \| 5 \| 3 \| \| 11 \| 8 \| \| \| 2 \| 2 \| \| 2 \| 2 \| \| 0 \| 0 \| \| >=3 \| \| 15 \| 13 \| \| 19 \| 9 \| \| 8 \| 19 \| \| \| 4 \| 7 \| \| 5 \| 7 \| \| 0 \| 9 \| \|  \| Intervention schools \| \| \| \| \| \| \| \| \| Control schools \| \| \| \| \| \| \| \| \| \| \|  \| All (n = 133) \| \| \| Urban (n = 86) \| \| \| Rural (n = 47) \| \| \| All (n = 54) \| \| \| \| Urban (n = 43) \| \| \| Rural (n = 11) \| \| \| \| CEREALS \| \| \| \| \| \| \| \| \| \| \| \| \| \| \| \| \| \| \| \| \|  \| \| pre \| post \| \| pre \| post \| \| pre \| post \| \| \| pre \| post \| \| pre \| post \| \| pre \| post \| \| <3 \| \| 0 \| 4 \| \| 0 \| 2 \| \| 0 \| 4 \| \| \| 6 \| 2 \| \| 7 \| 2 \| \| 0 \| 0 \| \| 3-4 \| \| 15 \| 15 \| \| 22 \| 20 \| \| 2 \| 6 \| \| \| 26 \| 33 \| \| 30 \| 37 \| \| 9 \| 18 \| \| 4-6 \| \| 21 \| 29 \| \| 17 \| 32 \| \| 28 \| 25 \| \| \| 31 \| 30 \| \| 33 \| 33 \| \| 27 \| 18 \| \| 7-8 \| \| 50 \| 39 \| \| 49 \| 36 \| \| 53 \| 45 \| \| \| 31 \| 33 \| \| 28 \| 26 \| \| 45 \| 64 \| \| >8 \| \| 13 \| 13 \| \| 12 \| 9 \| \| 17 \| 19 \| \| \| 6 \| 2 \| \| 2 \| 2 \| \| 18 \| 0 \| \| FRUIT AND VEGETABLES \| \| \| \| \| \| \| \| \| \| \| \| \| \| \| \| \| \| \| \| \|  \| \| pre \| post \| \| pre \| post \| \| pre \| post \| \| \| pre \| post \| \| pre \| post \| \| pre \| post \| \| <1 \| \| 47 \| 48 \| \| 55 \| 56 \| \| 32 \| 34 \| \| \| 65 \| 74 \| \| 65 \| 72 \| \| 64 \| 82 \| \| 1 \| \| 35 \| 34 \| \| 28 \| 29 \| \| 47 \| 43 \| \| \| 26 \| 22 \| \| 26 \| 23 \| \| 27 \| 18 \| \| 2 \| \| 13 \| 12 \| \| 13 \| 9 \| \| 13 \| 17 \| \| \| 9 \| 2 \| \| 9 \| 2 \| \| 9 \| 0 \| \| 3 \| \| 5 \| 4 \| \| 3 \| 2 \| \| 8 \| 6 \| \| \| 0 \| 2 \| \| 0 \| 2 \| \| 0 \| 0 \| \| >4 \| \| 1 \| 2 \| \| 1 \| 2 \| \| 0 \| 0 \| \| \| 0 \| 0 \| \| 0 \| 0 \| \| 0 \| 0 \|   Post-test measures were all significantly different between intervention and control schools (p<0.01). There was a significant difference between intervention and control groups at baseline for: dairy (>=3 servings) and cereals (7-8 servings and >8 servings). |
| **Secondary Outcomes SEP** | NA |
| **Primary Outcomes age/sex** | NA |
| **Secondary Outcomes age/sex** | NA |
| **Effect on inequalities** | **The change in % of children consuming >4 portions of fruit and vegetables per day is the primary outcome of interest. Since both the highest and lowest SEP achieved a significantly higher % of children in this category than the control group, this intervention has no impact on inequalities.** |
| **Study authors conclusions** | That the NEAPS is adaptable to different cultural environments. Knowledge items and preference shifted in the desired direction with a small impact on behaviours. |
| **Limitations** | The food diaries used were deemed not accurate enough for a full nutritional assessment. Mention that a debriefing with a dietician may have increased the accuracy of information. Some significant differences at baseline between intervention and control groups. This analysis was not run to compare rural vs. urban differences. |
| **Notes** |  |

| Haerens L, Deforche B, Maes L, Brug J, Vandelanotte C, De Bourdeaudhuij I: **A computer-tailored dietary fat intake intervention for adolescents: Results of a randomized controlled trial.** *Annals of Behavioral Medicine* 2007, **34:**253-262 | |
| --- | --- |
| **Funder(s)** | The Policy Research Centre for Sport, Physical Activity and Health and the Finnish Government. |
| **“P”** | Person |
| **Aim(s)** | To evaluate the acceptability, feasibility and effectiveness of a computer-tailored dietary fat intake education program for adolescents. |
| **Setting** | Secondary schools in Belgium. |
| **Recruitment and sample size** | 12 schools, 6 with general education (GE) (where the focus is on theoretical expertise) and 6 with technical-vocational education (TV) (where the focus is on practical skills) were randomly selected out of 52 eligible schools. 2 schools declined, leaving a random sample of 10 schools (5 GE and 5 TV). 304 of 399 students completed the study (90 male). |
| **Study design** | RCT |
| **Intervention(s) analysed** | Health education |
| **Nutrient** | Fat |
| **Methods/**  **Intervention details** | 2 classes from each school were randomly assigned to either intervention or control (control = no intervention). Students completed questionnaires at baseline and three months later. The intervention was an adaptation of an adult computer tailored dietary intervention. Students completed a 50 min CD-ROM alone which covered detailing the student’s dietary fat intake and then producing individual feedback based on their responses which could be saved. These messages covered self-efficacy, barriers, social support, perceived benefits and attitudes and were based on several psychological theories including the theory of planned behaviour and the transtheoretical model. This was piloted amongst children who were achieving lower grades. |
| **Length of follow up** | Three months |
| **SEP measurement** | Type of school – GE = high SEP, TV = low SEP |
| **Primary Outcomes SEP**  Pre and post-dietary fat intake levels (g/d) for gender and type of education   \| Total dietary fat intake (g/d) \| n \| Condition* \| Pre (M±SD)** \| Post (M±SD) \| \| --- \| --- \| --- \| --- \| --- \| \| Total GE \| 90 \| I \| 120.9±48.7 \| 108.2±43.9 \| \|  \| 65 \| I-read \| 118.4±50.1 \| 102±43.8 \| \|  \| 84 \| C \| 110±42.2 \| 107.3±41.5 \| \| Boys GE \| 41 \| I \| 130.4±56.1 \| 115.8±45.2 \| \|  \| 26 \| I-read \| 127.7±60 \| 111±49.9 \| \|  \| 30 \| C \| 120.3±47.8 \| 116.3±47.9 \| \| Girls GE \| 49 \| I \| 112.9±40.6 \| 101.8±42.2 \| \|  \| 39 \| I-read \| 112.1±42 \| 96±38.7 \| \|  \| 54 \| C \| 104.3±38 \| 102.3±37.1 \| \| Total TV \| 63 \| I \| 109.7±51.6 \| 99.6±51.3 \| \|  \| 46 \| I-read \| 97.8±38.9 \| 86.2±39.3 \| \|  \| 67 \| C \| 118.8±50.8 \| 110.5±47 \| \| Boys TV \| 9 \| I \| 143.4±56.6 \| 157.1±70.1 \| \|  \| 3 \| I-read \| 146.2±92.6 \| 165.1±110 \| \|  \| 10 \| C \| 119.7±57.6 \| 104.6±60.2 \| \| Girls TV \| 54 \| I \| 104.1±49.1 \| 90.1±41 \| \|  \| 43 \| I-read \| 94.4±32.2 \| 80.7±24.5 \| \|  \| 57 \| C \| 118.6±50.1 \| 111.5±44.9 \|   *Condition – I = intervention, I-read = children who had reported having actually read the intervention messages, C = control.  **M±SD = Mean and standard deviation. | |
| **Secondary Outcomes SEP** | NA |
| **Primary Outcomes age/sex** | NA |
| **Secondary Outcomes age/sex** | NA |
| **Effect on inequalities** | **The change in mean dietary fat intake is the primary outcome of interest. From examining the standard deviations given, there is no significant change in intake for either low or high SEP. Therefore this intervention had no impact on inequalities.** |
| **Study authors conclusions** | If this was to be run in the curriculum, at least one fourth of adolescents would not make an effort to read the intervention messages. However, the results show a positive impact of the tailored intervention on students who made the effort therefore showing that interventions like this have potential for use with adolescents. |
| **Limitations** | As a result of randomisation, boys were underrepresented in the study. This only looked at the short term – 3 months may not have been long enough to allow for substantial changes in the adolescents diet. Self-report. Having intervention and control classes within the same school may have resulted in some contamination. |
| **Notes** |  |

| Havas S, Anliker J, Damron D, Langenberg P, Ballesteros M, Feldman R: **Final results of the Maryland WIC 5-A-Day Promotion Program.** *American Journal of Public Health* 1998, **88:**1161-1167. | |
| --- | --- |
| **Funder(s)** | The National Cancer Institute and the Maryland Department of Health and Mental Hygiene. |
| **“P”** | Person |
| **Aim(s)** | Sought to increase fruit and vegetable consumption of women served by the Special Supplemental Nutrition Program for Woman, Infants and Children (WIC) by at least one half serving. |
| **Setting** | 16 WIC sites located in Baltimore city and 6 Maryland counties (8 intervention, 8 control). |
| **Recruitment and sample size** | Recruited women who were enrolled (or had a child enrolled) in a WIC directly from the 16 sites during voucher pickup. Had to state they had the intention to remain enrolled for a future 6 months. 3122 women took part – 1679 control, 1443 intervention |
| **Study design** | RCT |
| **Intervention(s) analysed** | Health education |
| **Nutrient** | Fruit and vegetables |
| **Methods/**  **Intervention details** | Intervention based on the stages of change model. Four months after completion of phase 1, Intervention sites became control sites and vice versa – same women couldn’t participate therefore each site acted as its own control. Peer educators – 2 types of nutrition education: i) brief messages regarding increasing the consumption of fruit and vegetables given at the time of enrolment into the study. ii) a series of 3 discussion sessions each lasting 45 minutes. Usually took place immediately after the regular bimonthly voucher distribution days. First session = self-assessment and goal setting, second session = identifying and overcoming potential barriers, third session = maintenance strategies. Each session had a food demonstration to build skills/allow them to try new foods. Sent 4 letters to participants over 6 months with tips. Written materials and visual aids were used throughout. |
| **Length of follow up** | 1 year |
| **SEP measurement** | Race (white, black and other), education (<HS = less than high school, HS = high school graduate, >HS = more than high school, and other) |
| **Primary Outcomes SEP** | Change in mean daily servings (SE) of fruit and vegetables consumed by SEG   \| Variable \| Control \| \| \| Intervention \| \| \| * \| \| --- \| --- \| --- \| --- \| --- \| --- \| --- \| --- \| \|  \| Baseline \| 8 months \| Change \| Baseline \| 8 months \| Change \| p \| \| <HS \| 3.68 (0.17) \| 4.04 (0.17) \| 0.36 (0.25 ) \| 3.53 (0.22) \| 3.66 (0.21) \| 0.12 (0.16) \| 0.43 \| \| HS \| 4.14 (0.11) \| 4.17 (0.1) \| 0.03 (0.12) \| 3.83 (0.18) \| 4.41 (0.1) \| 0.59 (0.14) \| 0.005 \| \| >HS \| 4.61 (0.13) \| 4.59 (0.14) \| -0.02 (0.15) \| 4.13 (0.16) \| 4.75 (0.16) \| 0.62 (0.14) \| 0.003 \| \| Other \| 3.57 (0.28) \| 3.88 (0.39) \| 0.31 (0.34) \| 4.33 (0.6) \| 4.57 (0.53) \| 0.24 (0.4) \| 0.89 \|   *Paired t test comparing change in intervention v change in control |
| **Secondary Outcomes SEP** | Change in mean daily servings (SE) of fruit and vegetables consumed by SEG   \| Variable \| Control \| \| \| Intervention \| \| \| * \| \| --- \| --- \| --- \| --- \| --- \| --- \| --- \| --- \| \|  \| Baseline \| 8 months \| Change \| Baseline \| 8 months \| Change \| p \| \| White \| 3.78 (0.24) \| 3.87 (0.22) \| 0.09 (0.09) \| 3.51 (0.19) \| 4.29 (0.16) \| 0.79 (0.11) \| <0.001 \| \| Black \| 4.43 (0.17) \| 4.61 (0.13) \| 0.17 (0.09) \| 4.22 (0.17) \| 4.58 (0.12) \| 0.36 (0.18) \| 0.37 \| \| Other \| 5.26 (0.64) \| 4.65 (0.52) \| -0.62 (0.77) \| 4.23 (0.48) \| 5.33 (0.6) \| 1.1 (0.6) \| 0.09 \|   *Paired t test comparing change in intervention v change in control |
| **Primary Outcomes age/sex** | Change in mean daily servings (SE) of fruits and vegetables consumed by age   \| Age \| Control \| \| \| Intervention \| \| \| * \| \| --- \| --- \| --- \| --- \| --- \| --- \| --- \| --- \| \|  \| Baseline \| 8 months \| Change \| Baseline \| 8 months \| Change \| p \| \| 18-24 \| 4.31 (0.15) \| 4.3 (0.13) \| -0.01 (0.1) \| 3.92 (0.17) \| 4.44 (0.11) \| 0.51 (0.17) \| 0.015 \| \| 25-29 \| 4.25 (0.18) \| 4.31 (0.18) \| 0.06 (0.14) \| 3.81 (0.15) \| 4.62 (0.17) \| 0.81 (0.13) \| 0.001 \| \| 30+ \| 3.98 (0.11) \| 4.31 (0.11) \| 0.33 (0.11) \| 3.9 (0.15) \| 4.39 (0.1) \| 0.5 (0.13) \| 0.36 \|   *paired t test comparing change in intervention v change in control |
| **Secondary Outcomes age/sex** | NA |
| **Effect on inequalities** | **The change in mean servings of fruit and vegetables consumed is the primary outcome of interest. The higher SEP achieved a significantly higher amount of fruit and vegetable consumption than the control group, while the lowest SEP failed to achieve this. This intervention widens inequalities.** |
| **Study authors conclusions** | Despite the programs overall success, this demonstrates again that programs seeking to prevent disease among low-income populations are exceedingly difficult to implement. Numerous challenges remain. |
| **Limitations** | Nonattendance at the nutrition sessions – barriers such as lack of transportation, lack of interest and work schedules – used an intention to teat analysis. They emphasised merely consuming more fruit and vegetables (to allow participants to set their own goals) rather than consuming 5 or more servings daily (more concerned with skewing the results relating to change in knowledge scores). Lower baseline consumption rates in the intervention group (controlled for this and changes remained significant). Does not detail what ‘other’ includes for race and education level. |
| **Notes** |  |

| Havas S, Anliker J, Greenberg D, Block G, Block T, Blik C, Langenberg P, DiClemente C: **Final results of the Maryland WIC food for life program.** *Preventive Medicine* 2003, **37:**406-416 | |
| --- | --- |
| **Funder(s)** | National Cancer Institute |
| **“P”** | Person |
| **Aim(s)** | Follows on from 1998 study. Goals this time were for intervention participants to show a net decrease of 2% in the % of calories from fat, a net increase of 0.5 servings of fruit and a net increase of 2g of fibre consumption compared to control participants. |
| **Setting** | 10 WIC sites (Special Supplemental Nutrition Program for Women, Infants and Children) in Baltimore and 5 Maryland counties, USA. |
| **Recruitment and sample size** | The peer educators dealt with recruitment directly with the women who were eligible and enrolled in the WIC. 1011 women in control, 1055 in intervention condition. |
| **Study design** | RCT |
| **Intervention(s) analysed** | Dietary counselling |
| **Nutrient** | Fruit and vegetables, Fibre, Fat |
| **Methods/**  **Intervention details** | Piloted intervention in 2 intervention and 1 control site. Then full trial = 10 sites randomised to 5 intervention and 5 control, this was then reversed after the intervention so the sites acted as their own controls. Peer educators were blinded as to control/intervention. Controls experienced the normal WIC program which devoted less than 10 minutes to these behaviours over 6 months. The multidimensional intervention program lasted 6 months and was targeted to participants’ stages of change and included: i) a 5 minute video featuring enthusiastic participants from the pilot; ii) brochures, newsletter, mail packets and individualised invitations; iii) individualised feedback on their baseline FFQ; iv) a kick off fair (including taste tests); v) four 45 minute workshops (including group discussions, practical advice on snacking, breakfast, dining out and product comparisons); vi) behaviour-reinforcing incentives and vii) phone calls. Participants completed FFQ before and after the intervention. Also measured stages of change with a 5 item classification matrix. |
| **Length of follow up** | 1 year follow up survey (not the results shown here) |
| **SEP measurement** | Race (black and white); education (<HS = less than high school, HS = high school graduate, and SC = some college) |
| **Primary Outcomes SEP** | Change in mean (SE) daily nutrient intake by SEG of participants - % calories from fat   \|  \| Control \| \| \| Intervention \| \| \| P* \| \| --- \| --- \| --- \| --- \| --- \| --- \| --- \| --- \| \|  \| Baseline \| 8 months \| Change \| Baseline \| 8 months \| Change \|  \| \| <HS \| 36 (0.57) \| 35.4 (0.56) \| -0.65 (0.54) \| 36 (0.48) \| 35 (0.53) \| -0.91 (0.5) \| 0.79 \| \| HS \| 35.3 (0.36) \| 36.3 (0.37) \| 1.01 (0.35) \| 35.4 (0.33) \| 34.2 (0.4) \| -1.23 (0.37) \| <0.001 \| \| SC \| 35 (0.33) \| 34.7 (0.39) \| -0.3 (0.36) \| 34.2 (0.34) \| 32.4 (0.4) \| -1.78 (0.38) \| 0.005 \|   Change in mean (SE) daily nutrient intake by SEG of participants – servings of fruit and vegetables   \|  \| Control \| \| \| Intervention \| \| \| P* \| \| --- \| --- \| --- \| --- \| --- \| --- \| --- \| --- \| \|  \| Baseline \| 8 months \| Change \| Baseline \| 8 months \| Change \|  \| \| <HS \| 3 (0.18) \| 2.8 (0.17) \| -0.22 (0.19) \| 3.3 (0.17) \| 3.5 (0.19) \| 0.13 (0.21) \| 0.22 \| \| HS \| 3.5 (0.12) \| 3.2 (0.12) \| -0.32 (0.12) \| 3.4 (0.12) \| 3.5 (0.13) \| 0.14 (0.12) \| 0.006 \| \| SC \| 3.7 (0.12) \| 3.5 (0.12) \| -0.17 (0.12) \| 3.6 (0.11) \| 3.8 (0.12) \| 0.2 (0.12) \| 0.04 \|   Change in mean (SE) daily nutrient intake by SEG of participants – g of fibre   \|  \| Control \| \| \| Intervention \| \| \| P* \| \| --- \| --- \| --- \| --- \| --- \| --- \| --- \| --- \| \|  \| Baseline \| 8 months \| Change \| Baseline \| 8 months \| Change \|  \| \| <HS \| 12.4 (0.6) \| 11.6 (0.51) \| -0.81 (0.62) \| 14.1 (0.59) \| 14 (0.62) \| -0.08 (0.61) \| 0.51 \| \| HS \| 13.7 (0.33) \| 12.9 (0.33) \| -0.74 (0.32) \| 13.1 (0.32) \| 13.2 (0.36) \| 0.17 (0.31) \| 0.04 \| \| SC \| 13.5 (0.32) \| 13.2 (0.35) \| -0.33 (0.32) \| 13.7 (0.37) \| 14.6 (0.41) \| 0.87 (0.38) \| 0.01 \|   *intervention v control |
| **Secondary Outcomes SEP** | Change in mean (SE) daily nutrient intake by SEG of participants - % calories from fat   \|  \| Control \| \| \| Intervention \| \| \| P* \| \| --- \| --- \| --- \| --- \| --- \| --- \| --- \| --- \| \|  \| Baseline \| 8 months \| Change \| Baseline \| 8 months \| Change \|  \| \| Black \| 36.5 (0.3) \| 36 (0.32) \| -0.5 (0.29) \| 36.9 (0.28) \| 35 (0.33) \| -0.95 (0.33) \| 0.29 \| \| White \| 33.8 (0.34) \| 34.9 (0.39) \| 1.11 (0.37) \| 33.9 (0.33) \| 32.1 (0.39) \| -1.82 (0.35) \| <0.001 \|   Change in mean (SE) daily nutrient intake by SEG of participants – servings of fruit and vegetables   \|  \| Control \| \| \| Intervention \| \| \| P* \| \| --- \| --- \| --- \| --- \| --- \| --- \| --- \| --- \| \|  \| Baseline \| 8 months \| Change \| Baseline \| 8 months \| Change \|  \| \| Black \| 3.7 (0.11) \| 3.3 (0.11) \| -0.39 (0.11) \| 3.5 (0.1) \| 3.6 (0.11) \| 0.09 (0.11) \| 0.004 \| \| White \| 3.2 (0.11) \| 3.2 (0.11) \| -0.02 (0.11) \| 3.4 (0.1) \| 3.7 (0.12) \| 0.27 (0.11) \| 0.05 \|   Change in mean (SE) daily nutrient intake by SEG of participants – g of fibre   \|  \| Control \| \| \| Intervention \| \| \| P* \| \| --- \| --- \| --- \| --- \| --- \| --- \| --- \| --- \| \|  \| Baseline \| 8 months \| Change \| Baseline \| 8 months \| Change \|  \| \| Black \| 13.7 (0.29) \| 13 (0.31) \| -0.69 (0.3) \| 13.5 (0.31) \| 13.9 (0.36) \| 0.33 (0.32) \| 0.02 \| \| White \| 12.8 (0.32) \| 12.5 (0.3) \| -0.35 (0.31) \| 13.3 (0.34) \| 13.8 (0.35) \| 0.54 (0.34) \| 0.05 \|   *intervention v control |
| **Primary Outcomes age/sex** | Change in mean (SE) daily nutrient intake by age of participants - % calories from fat   \|  \| Control \| \| \| Intervention \| \| \| P* \| \| --- \| --- \| --- \| --- \| --- \| --- \| --- \| --- \| \|  \| Baseline \| 8 months \| Change \| Baseline \| 8 months \| Change \|  \| \| 18-24 \| 34.3 (0.35) \| 34.7 (0.37) \| 0.37 (0.35) \| 34 (0.32) \| 33.1 (0.4) \| -0.87 (0.36) \| 0.01 \| \| 25-29 \| 34.9 (0.45) \| 36 (0.5) \| 1.1 (0.46) \| 35.7 (0.43) \| 33.6 (0.53) \| -2.17 (0.51) \| <0.0001 \| \| 30+ \| 36.8 (0.38) \| 36.2 (0.44) \| -0.66 (0.4) \| 35.7 (0.36) \| 34.3 (0.41) \| -1.49 (0.4) \| 0.14 \|   Change in mean (SE) daily nutrient intake by age of participants – servings of fruit and vegetables   \|  \| Control \| \| \| Intervention \| \| \| P* \| \| --- \| --- \| --- \| --- \| --- \| --- \| --- \| --- \| \|  \| Baseline \| 8 months \| Change \| Baseline \| 8 months \| Change \|  \| \| 18-24 \| 3.5 (0.12) \| 3.2 (0.12) \| -0.36 (0.12) \| 3.6 (0.12) \| 3.5 (0.13) \| -0.04 (0.12) \| 0.07 \| \| 25-29 \| 3.7 (0.17) \| 3.4 (0.16) \| -0.28 (0.17) \| 3.2 (0.13) \| 3.6 (0.16) \| 0.4 (0.16) \| 0.003 \| \| 30+ \| 3.4 (0.12) \| 3.3 (0.12) \| -0.07 (0.13) \| 3.6 (0.12) \| 3.8 (0.14) \| 0.24 (0.14) \| 0.1 \|   Change in mean (SE) daily nutrient intake by SEG of participants – g of fibre   \|  \| Control \| \| \| Intervention \| \| \| P* \| \| --- \| --- \| --- \| --- \| --- \| --- \| --- \| --- \| \|  \| Baseline \| 8 months \| Change \| Baseline \| 8 months \| Change \|  \| \| 18-24 \| 13.3 (0.34) \| 12.4 (0.32) \| -0.9 (0.33) \| 13.2 (0.35) \| 13.1 (0.37) \| -0.08 (0.31) \| 0.06 \| \| 25-29 \| 13.5 (0.44) \| 13.1 (0.44) \| -0.36 (0.42) \| 13 (0.41) \| 14.1 (0.51) \| 1.11 (0.46) \| 0.03 \| \| 30+ \| 13.5 (0.36) \| 13.2 (0.4) \| -0.32 (0.38) \| 14.2 (0.42) \| 14.7 (0.43) \| 0.53 (0.43) \| 0.13 \|   *intervention v control |
| **Secondary Outcomes age/sex** | NA |
| **Effect on inequalities** | **The change in mean servings of fruit and vegetables consumed is the primary outcome of interest. The higher SEP achieved a significantly higher amount of fruit and vegetable consumption than the control group, while the lowest SEP failed to achieve this. This intervention widens inequalities.** |
| **Study authors conclusions** | The significant differences in nutrient variables that the Maryland WIC FFL program achieved between intervention and control participants were substantially larger than those attained in similar intervention previously. The results indicate that this program may merit replication within the WIC program, as well as in other settings with frequent contact with target low-income populations. |
| **Limitations** | Baseline differences in control and intervention were due to seasonality. Nonattendance at the sessions was a problem – there was a change in the frequency of voucher distribution during the study, meaning these people attending the sessions would have had to have made a specific effort to attend. Used intention to treat. Self-report and social desirability in the FFQ |
| **Notes** | This again is described as being aimed at only low SEG (women who qualify to be enrolled in a WIC have to be 180% below the poverty level) but they do split results by proxies of SEG? Authors openly say that sub group analyses were not pre-planned. |

| Holme I, Hjermann I, Helgeland A, Leren P: **The Oslo Study: diet and antismoking advice. Additional results from a 5-year primary preventive trial in middle-aged men.** *Preventive Medicine*, **14:**279-292. | |
| --- | --- |
| **Funder(s)** | Not stated |
| **“P”** | Person |
| **Aim(s)** | To show whether the lowering of serum lipids and cessation of smoking could reduce the incidence of CHD |
| **Setting** | Oslo, Norway |
| **Recruitment and sample size** | All men between the ages of 40 and 49 in Oslo, Norway, were invited for a coronary risk factor screening. 65% (16,202) responded and came for the screening, from which 1,232 were chosen. These were healthy coronary high risk candidates. 604 men in intervention group, 628 men in control group. |
| **Study design** | RCT |
| **Intervention(s) analysed** | Dietary counselling |
| **Nutrient** | Fat |
| **Methods/**  **Intervention details** | Those in the intervention group were given dietary counselling to lower serum cholesterol levels and smokers were advised to quit smoking. The dietary advice consisted mainly of instructions to reduce the intake of saturated fat and cholesterol and to increase the intake of polyunsaturated fat. Those who were overweight were advised to reduce their caloric intake. Dietary and antismoking counselling was repeated every six months, along with a clinical and electrocardiographic examination and recording of risk-factor levels. The control group met once a year for the same examination procedures. A dietary history was recorded for every participant at the start of the trial, and in the intervention group, at every follow-up |
| **Length of follow up** | 7 and a half years (trial lasted 5 years, 2.5 years follow up) |
| **SEP measurement** | Social Class (Low = IV and V, middle = III, high = I +II) |
| **Primary Outcomes SEP** | % reduction in cholesterol (mg/d) by social class over time (5 years of the trial)   \| Social class \| Intervention \| Control \| \| --- \| --- \| --- \| \| I+II \| -46 \| -12 \| \| III \| -35 \| -9 \| \| IV \| -39 \| -7 \| \| V \| -29 \| -5 \| |
| **Secondary Outcomes SEP** | Incidence of CHD by social class in intervention and control groups   \| Social Class \| Intervention \| \| \| Control \| \| \| Total (excluding unknown) \| \| \| \| --- \| --- \| --- \| --- \| --- \| --- \| --- \| --- \| --- \| --- \| \|  \| At risk \| CHD cases \| % rate \| At risk \| CHD cases \| % rate \| At risk \| CHD cases \| % rate \| \| I+II \| 400 \| 10 \| 2.5 \| 394 \| 19 \| 4.8 \| 794 \| 29 \| 3.7 \| \| III \| 147 \| 8 \| 5.4 \| 163 \| 12 \| 7.4 \| 310 \| 20 \| 6.5 \| \| IV+V \| 38 \| 1 \| 2.6 \| 51 \| 5 \| 9.8 \| 89 \| 6 \| 6.7 \| \| Total \| 585 \| 19 \| 3.2 \| 608 \| 36 \| 5.9 \| 1193 \| 55 \| 4.6 \|   Other changes that were reported by social class relate to the smoking intervention component |
| **Primary Outcomes age/sex** | NA |
| **Secondary Outcomes age/sex** | NA |
| **Effect on inequalities** | **The % reduction in cholesterol is the primary outcome of interest. Since there are no measurement errors or confidence intervals concerning this information, only a comparison of the magnitude of change between the lowest and highest SEP can be made. The highest SEP achieved a larger reduction in cholesterol than the lowest SEPs. This intervention appears to increase inequalities but it cannot be inferred that this is a significant difference in change between the groups.** |
| **Study authors conclusions** | The men in the intervention group were at lower risk of CHD incidence than controls across all social strata, which fits well with the observed group differences in serum cholesterol among the various social strata, but not so well with regard to smoking habits. This observation supports the view of serum cholesterol as a key modifiable causal factor for CHD |
| **Limitations** | The authors do not list any – only conducted in men, authors note that some intervention materials were given to the men’s wives to help with make dietary changes at home – for those men who were not married, they may have been disadvantaged as a result. |
| **Notes** |  |

| Jeffery RW, French SA: **Preventing weight gain in adults: design, methods and one year results from the Pound of Prevention study.** *International journal of obesity and related metabolic disorders: journal of the International Association for the Study of Obesity* 1997, **21:**457-464. | |
| --- | --- |
| **Funder(s)** | National Institute of Diabetes and Digestive and Kidney Diseases; Centres for Disease Control and Prevention. |
| **“P”** | Person |
| **Aim(s)** | To determine whether an intervention involving an education program and a financial incentive could reduce weight gain on a larger scale that has been done previously. The authors present one year of results of this prospective 5 year intervention. |
| **Setting** | Suburban communities in St. Paul Minnesota and Minneapolis. |
| **Recruitment and sample size** | Recruited from a variety of sources – generally through direct phone solicitation in neighbourhoods with a sociodemographic representation similar to the metropolitan area as a whole, newspaper ads and email adverts to employees of the University of Minnesota. 228 men, 594 high income women and 404 low income women. |
| **Study design** | RCT |
| **Intervention(s) analysed** | Health education |
| **Nutrient** | Fat, fruit and vegetables |
| **Methods/**  **Intervention details** | One half of the participants were randomised to control, and one quarter to each of the intervention conditions which was education only (E-) and education plus lottery incentive (E+). At baseline and each year of the study, participants had their height and weight measured and completed a battery of questionnaires. Intervention based on the hypothesis that gradual weight gain in adults is due to people not being attentive to small changes in weight that could be prevented from worsening. Both intervention groups got the same educational and behavioural messages: i) pay attention to your weight; ii) eat 2 portions of fruit and 3 portions of veg daily; iii) reduce intake of high fat foods iv) walk 3 times a week for 20 minutes. These messages were delivered to them in monthly newsletters. Each newsletter included self-addressed stamped postcards for them to complete and return detailing their previous days intake/activity. There were 2 classes in the first year of the study also for the intervention group covering nutrition and physical activity. The only difference between E- and E+ was E+ participants were entered in a lottery to win $100 every month. |
| **Length of follow up** | 1 year |
| **SEP measurement** | Female income (low income = total household income of <= $25,000) |
| **Primary Outcomes SEP** | Adjusted mean (SEM) change in weight and behavioural variables in high and low income women by treatment group over 1 year of observation   \| Variable \| Control \| \| E- \| \| E+ \| \| \| --- \| --- \| --- \| --- \| --- \| --- \| --- \| \|  \| High \| Low \| High \| Low \| High \| Low \| \| Weight (lb) \| 1.38 (0.44) \| 1.3 (0.72) \| 1.03 (0.66) \| 2.11 (0.99) \| 0.51 (0.64) \| 3.23 (0.98) \| \| Energy intake (kcal/d) \| -62 (29) \| -218 (79) \| -66 (44) \| -336 (108) \| -27 (42) \| -208 (104) \| \| Fat calories (%) \| -0.87 (0.06) \| -1.04 (0.6) \| -1.98 (0.59) \| -2.49 (0.81) \| -1.96 (0.56) \| -1.76 (0.79) \| \| Veg per day \| 0.13 (0.06) \| -0.12 (0.1) \| -0.05 (0.09) \| 0.07 (0.13) \| 0.02 (0.09) \| 0.02 (0.13) \| \| Fruit per day \| -0.01 (0.05) \| -0.14 (0.06) \| -0.001 (0.07) \| -0.05 (0.09) \| 0.1 (0.07) \| -0.02 (0.13) \|   Controlling for age, ethnicity, smoking, baseline value for each variable |
| **Secondary Outcomes SEP** | NA |
| **Primary Outcomes age/sex** | NA |
| **Secondary Outcomes age/sex** | NA |
| **Effect on inequalities** | **The mean change in the women’s weight is the primary outcome of interest. The SEMs given in relation to mean weight change indicate that there is no significant difference in the amount of weight lost between the high and low SEP. Both of these policies have no impact on inequalities.** |
| **Authors conclusions** | These results suggest that the intervention may be having a greater impact on high than low income participants. Further follow up will reveal whether the low intensity educational strategy being tested is effective in reducing rate of weight gain in the groups being studied. |
| **Limitations** | Self-report; little interest and attendance to the one to one sessions element of the intervention. The presentation of the results Is confusing in this paper. |
| **Notes** |  |

| Jouret B, Ahluwalia N, Dupuy M, Cristini C, Nègre-Pages L, Grandjean H, Tauber M: **Prevention of overweight in preschool children: results of kindergarten-based interventions.** *International Journal of Obesity* 2009, **33:**1075-1083 | |
| --- | --- |
| **Funder(s)** | Not specified |
| **“P”** | Person |
| **Aim(s)** | To evaluate interventions with young children that could be incorporated into existing schools, community and health-care programs. Therefore implemented a kindergarten-based study in children (3-4 years old) to test two strategies with the primary objective of reducing the prevalence of childhood overweight at 5-6 years of age. |
| **Setting** | Kindergartens in France |
| **Recruitment and sample size** | Parents of children in the first or second section of kindergarten were sent flyers explaining the study and an invitation to participate. Posters emphasising the prevention of obesity were placed at the entrance to the kindergarten in order to reinforce this. After loss to follow up, 1253 children took part from 79 kindergartens. |
| **Study design** | RCT |
| **Intervention(s) analysed** | Health education |
| **Nutrient** | Multiple (sugar mentioned specifically in the description of the educational content) |
| **Methods/**  **Intervention details** | Study called “Epidemioloie et prevention de l’obesite infantile (EPIPOI). Two intervention = EPIPOI-1 and EPIPOI-2. Both interventions consisted of a basic strategy, with EPIPOI-2 having an additional reinforcement strategy. Basic strategy = baseline parents gave child’s medical records. Children underwent a medical visit with a study physician. Parents of children who were identified as being overweight or at risk of being so were sent a letter explaining the importance of bringing their child to their physician. These children’s physicians were also contacted to encourage follow up care. The reinforcement strategy = educational intervention – ten 20 minute sessions (5 a school year) which aimed to promote healthy nutrition habits (e.g. eating balances meals and snacks, limiting sugar sweetened beverages, drinking water etc), practicing physical activity and reducing sedentary behaviour. Children got audio story books to reinforce these messages at home while the parents got information packs. The effectiveness of the strategy was determined by comparing these children to children who were in kindergartens who had no intervention by examining their school medical records (1295 children). Data only extracted for EPIPOI-2 as this is the most appropriate intervention. |
| **Length of follow up** | 2 academic years |
| **SEP measurement** | Area the kindergarten was located – privileged/underprivileged |
| **Primary Outcomes SEP** | number of children overweight (BMI >=90^th^ percentile) at baseline and the end of the study (% in brackets) by group and school area (privileged, n = 591, underprivileged, n = 106)   \| Time/area \| EPIPOI-2 \| Control \| P value EPIPOI v control \| \| --- \| --- \| --- \| --- \| \| Underprivileged/baseline \| 13 (12.3) \| 15 (14.2) \| 0.685 \| \| Underprivileged/end \| 18 (17) \| 39 (36.8) \| 0.001 \| \| Privileged/baseline \| 49 (8.3) \| 20 (6.6) \| 0.363 \| \| Privileged/end \| 61 (10.3) \| 34 (11.2) \| 0.691 \| |
| **Secondary Outcomes SEP** | Evolution of median BMI z-score (IQR) by group and school area   \| Time/area \| EPIPOI-2 \| Control \| \| --- \| --- \| --- \| \| Underprivileged/baseline \| -0.37 (-0.97; 0.46) \| -0.26 (-1.39; 0.82) \| \| Underprivileged/end \| 0.14 (-0.49; 1.06) \| 0.71 (-0.21; 1.87) \| \| Privileged/baseline \| -0.48 (-1.22; 0.43) \| -0.48 (-1.3; 0.22) \| \| Privileged/end \| -0.19 (-0.97; 0.59) \| -0.04 (-0.82; 0.7) \|   At baseline and at the end, group significantly differed from the control group (smallest p = 0.002). |
| **Primary Outcomes age/sex** | NA |
| **Secondary Outcomes age/sex** | NA |
| **Effect on inequalities** | **The prevalence of overweight is the primary outcome of interest. At the end of the intervention, the underprivileged children had a significantly lower prevalence of overweight than the control group, whereas the privileged children did not. The absolute number of overweight children in both SEP’s increased over time, however, considering the difference between both groups with their control schools at the end of the intervention, this intervention may have prevented more potential overweight children from increasing their CMI in these schools. Therefore this intervention reduces inequalities.** |
| **Authors conclusions** | Simple early intervention reduced the risk of overweight compared with the control group in underprivileged areas. The additional educational component (EPIPOI-2) did not yield any improvement over the basic strategy (EPIPOI-1) in the sample as a whole (but in privileged children, this was effective in terms of change in BMI z-score as compared to the control group and the basic intervention. |
| **Limitations** | The control group was not recruited in a similar way and therefore differed in age and area as the original sample diminished more than originally expected due to drop out (accounted for in the analysis). Cannot determine longer term effects of the intervention as it only lasted two academic years. Also, even though the parents of overweight or at risk children were sent letters to advise them to take these children to their physicians, this was not followed up. |
| **Notes** |  |
| Lowe CF, Horne PJ, Tapper K, Bowdery M, Egerton C: **Effects of a peer modelling and rewards-based intervention to increase fruit and vegetable consumption in children.** *European Journal of Clinical Nutrition* 2004, **58:**510-522 | |
| **Funder(s)** | Department of Health, Department for Education and Employment, Department of Environment, Food and Rural Affairs and the Food Standards Agency. |
| **“P”** | Person |
| **Aim(s)** | To draw upon the literature of children’s learning and imitation and carry out an intervention that influences children to repeatedly taste fruit and vegetables and to sustain their consumption of these foods over time. |
| **Setting** | 3 primary schools in different parts of the UK |
| **Recruitment and sample size** | Evaluated in three schools: Bangor, North Wales (n = 10-5, age 4-11); Harwell, Oxfordshire (n = 134, age = 4-11) and Salford, Manchester (n = 163, age = 5-11). Each school was selected by its local health promotion unit or education authority to represent either higher or lower SEG as assessed by free meal entitlement. |
| **Study design** | Cohort study |
| **Intervention(s) analysed** | Health education |
| **Nutrient** | Fruit and vegetables |
| **Methods/**  **Intervention details** | 8-12 day baseline phase followed by a 16 day intervention phase. At “snack time” throughout baseline and intervention, the children were given a “snack pack” that contained two 20g portions of wither fruit or, on alternate days, raw vegetables. In each school, 4 different fruits and four different vegetables were presented in a fixed cycle. Each was presented 3 times at baseline, 4 times during the intervention. At lunch, children who had school lunches received a serving of fruit or veg. This who brought their own lunches, were given the equivalent of what as presented at “snack time”. Materials used were six 6 minute videos of “the food dudes” who fought the “junk punks” who plan to take over the world by depriving people of life giving fruit and vegetables. There were reward systems in place for completing activities (stickers, pens etc.) In Salford, there were home packs also to help parents become involved in the project which included yips about how to get the children to achieve 5 a day. The difference between baseline and intervention = at baseline the foods were just given out, at intervention they were given out while the teacher read out a food dude letter or showed a video, with rewards for eating all of the fruit and vegetables. Amount of fruit and veg consumed was recorded via visual estimation. Home consumption was measured in Salford also via a FFQ the parents completed. |
| **Length of follow up** | Study lasted approximately 28 days with no follow up |
| **SEP measurement** | School – deprivation level (measured by free meal entitlement) in Harwell = 6%, Bangor = 10%, and Salford = 31% (national average = 17%). |
| **Primary Outcomes SEP** | The mean % of fruit and vegetables consumed during the baseline and intervention phases in the three schools (in some schools there was just one presentation of a food at baseline so there is only one value). Baseline 1 = first day of baseline, baseline 2 = last day of baseline, intervention 1 = first day of intervention, intervention 2 = last day of intervention. Each child had an average calculated. Children with lunchboxes – only additional fruit and veg that was presented to them was calculated.  Fruit   \|  \| Baseline1 \| Baseline2 \| Intervention1 \| Intervention2 \| \| --- \| --- \| --- \| --- \| --- \| \| Harwell & Bangor \| 50 \| --- \| 86 \| 87 \| \| Salford \| 33 \| 21 \| 68 \| 74 \|   Vegetables   \|  \| Baseline1 \| Baseline2 \| Intervention1 \| Intervention2 \| \| --- \| --- \| --- \| --- \| --- \| \| Harwell & Bangor \| 26 \| --- \| 64 \| 68 \| \| Salford \| 9 \| 9 \| 48 \| 44 \| |
| **Secondary Outcomes SEP** | NA |
| **Primary Outcomes age/sex**  Estimated increases, following the intervention, in the number of portions (s.d.’s in parentheses) and grams of fruit and vegetables consumed per weekday by children aged 4-7 and 7-11.   \|  \| Lunchtime \| \| Snack time \| \| Home \| \| Total \| \| --- \| --- \| --- \| --- \| --- \| --- \| --- \| --- \| \|  \| Fruit \| Veg \| Fruit \| Veg \| Fruit \| Veg \|  \| \| 4-7 \| 0.81 (0.42); 49g \| 0.41 (0.29); 25g \| 0.21 (0.21); 13g \| 0.18 (0.19); 11g \| 0.62 (1.95); 37g \| 0.31 (1.05); 19g \| 2.54; 153g \| \| 7-11 \| 0.6 (0.6); 36g \| 0.48 (0.31); 29g \| 0.19 (0.19); 11g \| 0.24 (0.21 ); 14g \| 0 (1.01); 0g \| 0.67 (1.55); 40g \| 2.18; 131g \| | |
| **Secondary Outcomes age/sex** | NA |
| **Effect on inequalities** | **Since there are no measurement errors or confidence intervals concerning this information, only a comparison of the magnitude of change between the lowest and highest SEP can be made. Salford has reportedly lower fruit and vegetable consumption than the other higher SEP schools at the end of the intervention. This intervention widens inequalities however this may not be a significant effect.** |
| **Study authors conclusions** | This evidence suggests a hypothesis that runs directly counter to the notion that rewards for eating particular foods produce decrements in preference and consumption of these foods. The better the rewards, both social and tangible, for eating particular foods and the more trials on which they are presented, the greater will be the subsequent increase in liking and consumption of those foods. |
| **Limitations** | Parental recall measure was not calculated. The increase in fruit and veg consumption achieved in this study is much greater than other similar studies I the literature – this may be due to what is considered “a portion” to children of these ages. These may be over/under estimates. |
| **Notes** |  |

| Plachta-Danielzik S, Pust S, Asbeck I, Czerwinski-Mast M, Langnase K, Fischer C, Bosy-Westphal A, Kriwy P, Muller MJ: **Four-year follow-up of school-based intervention on overweight children: the KOPS study.** *Obesity*, **15:**3159-3169 | |
| --- | --- |
| **Funder(s)** | Deutsche Forschungsgemeinschaft and the World Cancer Research Fund-UK |
| **“P”** | Person |
| **Aim(s)** | To evaluate the 4 year outcome of a school based health promotion intervention on weight status as part of The Kiel Obesity Prevention Study (KOPS). This investigates the determinants and preventive measures of childhood overweight. |
| **Setting** | 32 primary schools in Kiel, North Germany |
| **Recruitment and sample size** | Parents of the children were contacted and required to give written permission. 1764 children aged 6-10 years old. (345 intervention, 1419 no intervention) |
| **Study design** | RCT |
| **Intervention(s) analysed** | Health education |
| **Nutrient** | Fat/fruit and veg |
| **Methods/**  **Intervention details** | KOPS is an 8 year cross sectional and longitudinal study. This report is giving the findings after 4 years. Intervention = messages (eat fruit and veg every day, reduce intake of high-fat foods, keep active at least 1 h/d) were given to the children, parents and teachers. Children in intervention schools had 6 lessons on healthy nutrition – done as interactive games, fairy-tale story-telling and a practical teaching how to prepare a healthy breakfast. Each lesson then had an accompanying 20 minute physical activity. Teachers were trained by nutritionists for half a day. Schools were randomised to intervention every year to give all schools a chance at the intervention. |
| **Length of follow up** | 4 years |
| **SEP measurement** | SES – high, middle and low |
| **Primary Outcomes SEP**  Adjusted* ORs (95% CI) for prevalence of overweight and obesity by SEP   \|  \| Overweight (T1) \| \| Obesity (T1) \| \| \| --- \| --- \| --- \| --- \| --- \| \|  \| OR (95% CI) \| p \| OR (95% CI) \| p \| \| Low \| 1.31 (0.69-2.46) \| 0.406 \| 0.52 (0.17-1.62) \| 0.258 \| \| Middle \| 1.03 (0.51-2.07) \| 0.935 \| 1.18 (0.27-5.22) \| 0.824 \| \| High \| 0.35 (0.14-0.91) \| 0.031 \| 1.23 (0.28-5.39) \| 0.786 \|   *Adjusted for baseline BMI, sex, SES and BMI of the mother as well as clustering effect among schools (OR of No intervention was taken as 1) | |
| **Secondary Outcomes SEP**  NA | |
| **Primary Outcomes age/sex**  Adjusted* ORs (95% CI) for prevalence of overweight and obesity by sex   \|  \| Overweight (T1) \| \| Obesity (T1) \| \| \| --- \| --- \| --- \| --- \| --- \| \|  \| OR (95% CI) \| p \| OR (95% CI) \| p \| \| Boys \| 0.88 (0.48-1.64) \| 0.697 \| 0.87 (0.32-2.32) \| 0.778 \| \| Girls \| 0.86 (0.48-1.53) \| 0.602 \| 0.9 (0.32-2.52) \| 0.848 \|   *Adjusted for baseline BMI, sex, SES and BMI of the mother as well as clustering effect among schools (OR of No intervention was taken as 1) | |
| **Secondary Outcomes age/sex**  NA | |
| **Effect on inequalities** | **The change in the prevalence of overweight is the primary outcome of interest. The confidence intervals indicate that the higher SEG significantly reduce their odds for prevalence of overweight more than that of the lower SEG. This intervention widens inequalities.** |
| **Study authors conclusions** | A school based health promotion has sustainable effects on remission and incidence of overweight – this was most pronounced in children from families with high SES. There was no effect on obesity. These results argue in favour of additional measures of prevention. |
| **Limitations** | Dropout rate was high. Prevalence’s of overweight and children from low SES families were higher in dropout rate compared to other participants. The differences between intervention and no intervention were marginally different at baseline, suggesting internal validity (e.g. % of children who profited from the intervention may have been lower in both groups but the absolute difference between intervention and no intervention was the same. The higher prevalence of overweight mothers in no intervention compared to intervention may cause selection bias – might lead to an overestimation of the intervention effect. In addition stratification reduces study power – much larger number in no intervention therefore has a higher power. |
| **Notes** |  |

| Reynolds KD, Franklin FA, Binkley D, Raczynski JM, Harrington KF, Kirk KA, Person S: **Increasing the Fruit and Vegetable Consumption of Fourth-Graders: Results from the High 5 Project.** *Preventive Medicine* 2000, **30:**309-319. | |
| --- | --- |
| **Funder(s)** | National Cancer Institute |
| **“P”** | Person |
| **Setting** | 28 elementary schools, Alabama, USA |
| **Aim(s)** | To increase fruit and vegetable consumption in the children and parents in the intervention schools |
| **Recruitment and sample size** | The authors recruited schools from three school districts. 1698 4^th^ grade students, mean age 8.7 years; 50% female; 83% European-American; 16% African-American; 1% other. |
| **Study design** | RCT |
| **Intervention(s) analysed** | Health education |
| **Nutrient** | Fruit and vegetables |
| **Methods/**  **intervention details** | Learning methods used in the classroom component included modelling, self-monitoring, problem-solving, reinforcement and taste testing, in a 14-lesson curriculum. This was given three days a week, with the first lesson of the week lasting 30-45 minutes. Completed workbooks and recapped previous lessons. Also had related homework. Parents were given information to encourage healthy changes at home and had to help their child to complete one interactive lesson from home per week. Cafeteria managers were given health food training and increased the amount of fruit and vegetables available on the premises. They also had to help promote fruit and vegetable consumption. Control schools had a delayed intervention. |
| **Length of follow up** | 2 years |
| **SEP measurement** | Household income |
| **Primary Outcomes SEP** | Number of servings of fruit and vegetable portions consumed per day (p relates to intervention v control)   \| SEP \| Condition \| 1 year \| 2 year \| \| --- \| --- \| --- \| --- \| \| Low \| Intervention \| 4, p = <0.0003 \| 3.1, p =<0.006 \| \| Control \| 2.7 \| 2.2 \| \| Medium \| Intervention \| 3.3, p = <0.03 \| 2.3, p = 0.004 \| \| Control \| 2.4 \| 2 \| \| High \| Intervention \| 4.4, p = <0.0001 \| 3.1, p = <0.01 \| \| Control \| 2 \| 2.1 \| |
| **Secondary Outcomes SEP** | NA |
| **Primary Outcomes age/sex** | Number of servings of fruit and vegetable portions consumed per day (p relates to intervention v control)   \| Gender \| Condition \| 1 year \| 2 year \| \| --- \| --- \| --- \| --- \| \| Boys \| Intervention \| 4.2, p = <0.001 \| 3.1, p = <0.01 \| \| Control \| 2.3 \| 2.2 \| \| Girls \| Intervention \| 3.6, p = <0.002 \| 3.3, p = <0.0001 \| \| Control \| 2.4 \| 2 \| |
| **Secondary Outcomes age/sex** | NA |
| **Effect on inequalities** | **The change in servings of fruit and vegetables consumed after 2 years is the primary outcome of interest. Both the lowest and highest SEP achieved a significantly higher number of fruit and vegetables consumed than the control group. The lowest SEP achieved 0.9 servings more than the control group whereas the highest SEP achieved 1 portion of fruit and vegetables consumed more than the control group. This intervention widens inequalities.** |
| **Authors conclusions** | In sum, strong effects were found for the High 5 intervention on fruit and vegetable consumption, on macro and micro-nutrients, and on psychosocial variables. Future work is needed to enhance the intervention effects on parent consumption and to test the effectiveness of the intervention when delivered by classroom teachers. |
| **Limitations** | No intervention effects were found in the lunchtime cafeteria observation data. It is possible that the 24 hour dietary recall data were subject to reporting favouring the intervention condition, and as a result the program effects could not be confirmed using the observational data. All children were observed, whereas not all children returned their food diaries. Non participants were likely to be consuming less fruit and vegetables hence the inclusion of these children may have diminished the intervention effects. |
| **Notes** |  |

| Smith AM, Owen N, Baghurst KI: **Influence of Socioeconomic Status on the Effectiveness of Dietary Counselling in Healthy Volunteers.** *Journal of Nutrition Education* 1997, **29:**27-35 | |
| --- | --- |
| **Funder(s)** | Commonwealth Scientific and Industrial Research Organisation (CSIRO) Division of Human Nutrition |
| **“P”** | Person |
| **Aim(s)** | To move the food intake pattern of the participants closer to that of the 12345+ Food and Nutrition Plan, thereby also reducing fat, saturated fat, sugar, and salt intakes and increasing vitamin, mineral (except sodium), polyunsaturated fat, fiber, and complex carbohydrate intakes." |
| **Setting** | Residential areas in Adelaide, Australia |
| **Recruitment and sample size** | Prospective participants were randomly selected from the Australian electoral Rolls, chosen from suburbs that were accessible to the intervention site. Letters were sent out inviting them to participate. Done in two drives – spring/summer and autumn/winter. 487 people (220 intervention, 227 control). 41% male; 34% aged 18-39, 34% aged 40-59, 32% aged 60+. 43% from higher SEG, 32% from middle SEG, 19% from low SEG |
| **Study design** | RCT |
| **Intervention(s) analysed** | Health education |
| **Nutrient** | Fat, Saturated fat, ratio of polyunsaturated to saturated fats (P:S ratio), fibre, salt and sugar |
| **Methods/**  **intervention details** | Those who volunteered were sent a FFQ and a demographics Q. These were handed in at the first visit and a blood test done to assess cholesterol test and BP, height and weight. Systematic alternate allocation of condition by higher or lower status suburb as prestige level was not known at this point. People could bring a friend to take part – if they did; they were both in the same group. In the intervention group, 2 weeks later a dietician talked them through their individual food profiles and based on this they were asked to set no more than 5 dietary goals. Materials were posted out at monthly intervals for the participants to record and monitor these goals. After three months they repeated the first FFQ and blood tests. The control group had no intervention until this point (at the end of the study) when they received the dietary advice session that the intervention group received at 2 weeks. |
| **Length of follow up** | 3 months |
| **SEP measurement** | The Daniel scale of occupational prestige – measure of individual socioeconomic advantage/disadvantage. Based on the distribution of scores in this study, the original quintile were collapsed down into three categories: highest quintile had a score between1.8 and 3.7, middle = 3.8 and 4.7 and lowest = 4.8 and 6.7 |
| **Primary Outcomes SEP** | Change in dietary fat density (g/4200 kcal)   \| Group \| High prestige \| Middle prestige \| Low prestige \| \| --- \| --- \| --- \| --- \| \| Intervention \| -0.58 \| -1.17 \| -1.75 \| \| Control \| -1.42 \| -0.25 \| +0.25 \|   Change in carbohydrate density (g/4200 kcal)   \| Group \| High prestige \| Middle prestige \| Low prestige \| \| --- \| --- \| --- \| --- \| \| Intervention \| +0.18 \| +0.27 \| +2.05 \| \| Control \| +1.3 \| -0.9 \| -0.45 \| |
| **Secondary Outcomes SEP** | NA |
| **Primary Outcomes age/sex** | NA |
| **Secondary Outcomes age/sex** | NA |
| **Effect on inequalities** | **The change in dietary fat density is the primary outcome of interest. Since there are no measurement errors or confidence intervals concerning this information, only a comparison of the magnitude of change between the lowest and highest SEP can be made. The lowest SEP decreases their recorded dietary fat density than the highest SEP. This intervention reduces inequalities but it cannot be inferred is this difference in the reported change is significant.** |
| **Study authors conclusions** | The dietary changes seen in this trial indicate that a one-time dietary counselling session, incorporating dietary and biological feedback, self-monitoring, and follow-up, can be equally efficacious in bringing about favourable dietary behaviour changes in both higher and lower socioeconomic status volunteers. However, this is a less effective way of reaching lower socioeconomic status groups compared to their higher status counterparts. |
| **Limitations** | Low recruitment rate from low SEP – may only have included those most interested with their dietary intake and concerned about changing it – this may have skews the results. Effectiveness of this program cannot be extrapolated to include those who were unemployed or who could not speak English as these were in very low numbers in the low SEP group. The sample at baseline already had below the level of 33% of energy from fat which is recommended in the dietary feedback. Self-report may have been an issue. Also, the use of friends and family in the study reduced the randomness of the allocation of participants to intervention and control groups. |
| **Notes** |  |

| Toft U, Jakobsen M, Aadahl M, Pisinger C, Jørgensen T: **Does a population-based multi-factorial lifestyle intervention increase social inequality in dietary habits? The Inter99 study.** *Preventive Medicine: An International Journal Devoted to Practice and Theory* 2012, **54:**88-93. | |
| --- | --- |
| **Funder(s)** | Research grants from the Danish Research Council, The Danish Centre for Health Technology Assessment, Novo Nordisk Inc., Research Foundation of Copenhagen County, Ministry of the Interior and Health, The Danish Heart Foundation, The Danish Pharmaceutical Association, The Augustinus Foundation, The Ib Henriksen Foundation, The Becket Foundation and The Health Insurance Foundation. |
| **“P”** | Person |
| **Setting** | Copenhagen, Denmark |
| **Aim(s)** | To investigate whether the effect of an individualised multi-factorial lifestyle intervention on dietary habits differs across socioeconomic groups. |
| **Recruitment and sample size** | A sample was invited to take part from an age-sex stratified sample of the entire population. 63% response rate, leaving 6019 participants in the analysis after exclusions and recruitment. A random sample of 5264 individuals was drawn (control group). |
| **Study design** | RCT |
| **Intervention(s) analysed** | Dietary counselling |
| **Nutrient** | Fruit |
| **Methods/**  **intervention details** | Based on a baseline personal risk estimate, each individual had a lifestyle counselling talk focussing on smoking, physical activity, diet and alcohol. The staff (doctors, nurses and dieticians) were all trained in health counselling and the motivational interviewing method. In addition to the health screening program and the individual counselling, high risk individuals were offered group counselling on diet and physical activity or smoking cessation or reduction, depending on lifestyle and motivation to change lifestyle. The relatives of the participants were offered to participate in one of the meetings. |
| **Length of follow up** | 5 years |
| **SEP measurement** | Education level (low and high) – this information was given by contact from the author |
| **Primary Outcomes SEP ***  Intake of fruit in g per week consumed by men with high and low education in men (n = 2962)  Data are mean ± standard deviation   \|  \| Intervention,  High education \| Intervention,  Low education \| Control,  High education \| Control,  Low education \| \| --- \| --- \| --- \| --- \| --- \| \| Baseline \| 667 ± 34 \| 597 ± 72 \| 673 ± 38 \| 570 ± 78 \| \| 1-year follow-up \| 775 ± 35 \| 619 ± 79 \| 806 ±39 \| 782 ± 81 \| \| 3-year follow-up \| 869 ±36 \| 734 ± 83 \| 796 ± 40 \| 692 ± 86 \| \| 5-year follow-up \| 917 ± 37 \| 815 ± 90 \| 910 ± 42 \| 702 ± 92 \|   *Contacted the author to get these results | |
| **Secondary Outcomes SEP** | NA |
| **Primary Outcomes age/sex** | NA |
| **Secondary Outcomes age/sex** | NA |
| **Effect on inequalities** | **Change in the amount of fruit men ate is the primary outcome of interest. The high SEP group has a mean increase of 250g of fruit consumed per week, while the low SEP has an increase of 218g of fruit consumed per week. Both changes are significant from examining the standard deviations given by the author, therefore this intervention benefits the higher SEP more than the low SEP and widens inequalities.** |
| **Study authors conclusions** | Individualised dietary interventions may decrease or hinder further widening of the social inequalities in health due to unhealthy dietary habits among socially disadvantaged individuals. However, there is a need for more high-quality studies investigating this issue. |
| **Limitations** | Low participation rate and high rate of loss to follow-up. Likely to be social inequality in the recruitment of study participants at baseline, i.e. individuals with no vocational training are likely to be overrepresented among non-participants. The results are based on subgroup analyses. |
| **Notes** | We only have the data pertaining to men |

**Additional articles examining ethnicity included in the sensitivity analysis**

| Blakely T, Ni Mhurchu C, Jiang Y, Matoe L, Funaki-Tahifote M, Eyles HC, Foster RH, McKenzie S, Rodgers A: **Do effects of price discounts and nutrition education on food purchases vary by ethnicity, income and education? Results from a randomised, controlled trial.** *Journal of epidemiology and community health* 2011, **65:**902-908. | |
| --- | --- |
| **Funder(s)** | Health research council of new Zealand. |
| **“P”** | Price |
| **Aim(s)** | To present pre planned analyses of the effects of the SHOP interventions (the Supermarket Healthy Options Project (SHOP) was an RCT (n = 1104)) by ethnicity, income and educational qualifications in order to provide more information about equity impacts of price discounts and tailored nutrition education on food purchasing. |
| **Setting** | Shoppers in Auckland, New Zealand. |
| **Recruitment and sample size** | To be eligible for the study, participants had to be either existing users of the handheld scanner system or willing to sign up and use the system for the duration of the trial. Most study participants were recruited through mail outs to randomly select existing registered scanner users. In addition, targeted in-store and community-based recruitment strategies involving Maori and Pacific recruiters were used to encourage participation of Maori and Pacific peoples – due to lower number of Maori and pacific scanner users, these were recruited to use these first to then become eligible to take part. N = Maori = 248, Pacific = 101 and European/other = 755. |
| **Study design** | RCT |
| **Intervention(s) analysed** | Price discounts |
| **Nutrient** | Saturated fat, fruit and vegetables |
| **Methods/**  **Intervention details** | The SHOP trial = 12 week baseline period, 24 week intervention period and 24 week follow up. Eligible participants were randomly assigned to one of the following four intervention arms: (i) price discounts (12.5%) on healthier supermarket foods, (ii) tailored nutrition education promoting purchase of healthier supermarket foods (including individual computer generated feedback and shopping lists promoting healthy alternatives) – Maori and pacific people were offered additional group sessions, (iii) a combination of price discounts and tailored nutrition education, or (iv) control (i.e. no intervention). |
| **Length of follow up** | 12 months |
| **SEP measurement** | Ethnicity – Maori, Pacific and European/other. |
| **Primary Outcomes SEP**  Estimates of effect of price discounts (n = 513) versus no price discounts (n = 515) on mean change* (SE) from baseline in nutrient and food purchases by ethnicity   \|  \| Maori \| \| Pacific \| \| European/other \| \| All participants \| \| \| --- \| --- \| --- \| --- \| --- \| --- \| --- \| --- \| --- \| \|  \| Mean (SE) \| 95% CI \| Mean (SE) \| 95% CI \| Mean (SE) \| 95% CI \| Mean (SE) \| 95% CI \| \| Saturated fat (%) \| \| \| \| \| \| \| \| \| \| Intervention (6 months) \| 0.35 (0.52) \| -0.69 to 1.39 \| 1.09 (0.74) \| -0.38 to 2.56 \| -0.34 (0.21) \| -0.75 to 0.06 \| -0.02 (0.19) \| -0.4 to 0.36 \| \| Follow-up (12 months) \| -0.48 (0.57) \| -1.6 to 0.65 \| -1.04 (0.81) \| -2.64 to 0.55 \| -0.06 (0.21) \| -0.47 to 0.36 \| -0.12 (0.2) \| -0.51 to 0.27 \| \| Discounted fruit and veg (food purchases) \| \| \| \| \| \| \| \| \| \| Intervention (6 months) \| -0.05 (0.37) \| -0.78 to 0.67 \| 0.86 (0.42) \| 0.03 to 1.69 \| 0.59 (0.16) \| 0.28 to 0.89 \| 0.48 (0.14) \| 0.21 to 0.75 \| \| Follow-up (12 months) \| 0.01 (0.39) \| -0.75 to 0.78 \| 0.65 (0.46) \| -0.25 to 1.56 \| 0.33 (0.16) \| 0.02 to 0.64 \| 0.28 (0.14) \| 0 to 0.56 \|   *Change from baseline with intervention versus change from baseline without intervention, adjusted for baseline nutrient and food purchases, ethnicity, income, age and sex.  Significant interaction with ethnicity at intervention period for sat fat (p = 0.02) but not at follow up (p = 0.3). No sig interactions with ethnicity for fruit and veg (smallest p = 0.13). | |
| **Secondary Outcomes SEP** | NA |
| **Primary Outcomes age/sex** | NA |
| **Secondary Outcomes age/sex** | NA |
| **Effect on inequalities** | **The change in % energy consumed from fat is the primary outcome of interest. The confidence intervals indicate that there is no significant difference in the change between the highest and lowest SEPs. This intervention has no impact on inequalities.** |
| **Study authors conclusions** | With regard to price discounts, there are good theoretical grounds for expecting the price elasticity to be greater among lower SEP, however this study was unable to demonstrate such a differential or pro-equity impact of price discounts on healthy food. |
| **Limitations** | Insufficient numbers to conduct separate sensitivity analyses by SEP and ethnicity. Numbers of Maori and Pacific shoppers were much less than were originally targeted, therefore CI values were wide. Some inconsistent results e.g. pacific people increased purchase of fatty foods by 1.09% with price discounts (however it can be argues that the price discounts on other foods enabled them to spend money on fatty foods). Systematic bias – higher attrition rate for Maori (17%) and Pacific (16%) shoppers than European/other (3%).Made the assumption that missing data occurred at random however this is not the case when given the adjusted covariates. Selection bias for Maori and Pacific people as they were not used to the scanners and were trained in advance. |
| **Notes** |  |

| Coates RJ, Bowen DJ, Kristal AR, Feng Z, Oberman A, Hall WD, George V, Lewis CE, Kestin M, Davis M, et al: **The Women's Health Trial Feasibility Study in Minority Populations: Changes in Dietary Intakes.** *American Journal of Epidemiology* 1999, **149:**1104-1112. | |
| --- | --- |
| **Funder(s)** | National Cancer Institute and the National Heart, Lung and Blood Institute. |
| **“P”** | Person |
| **Aim(s)** | The primary purpose of the Women's Health Trial Feasibility Study in Minority Populations (FSMP) was to determine whether a reduction in fat intake that had been obtained in the Vanguard Women's Health Trial could be achieved in a larger, more diverse population of postmenopausal women and whether the intervention could produce similar effects among women of different racial or ethnic groups and of different socioeconomic circumstances. This study also examined the intervention's effectiveness in reducing intakes of saturated fat and cholesterol and increasing intakes of fruits, vegetables, and grain products. Primary goal was to reduce total fat intake to 20% or less. |
| **Setting** | Clinics in Atlanta, Georgia; Birmingham, Alabama; and Miami, Florida, USA |
| **Recruitment and sample size** | Post-menopausal women aged 50-79 were invited to participate in what was called “a low-fat dietary modification study for women”. 2208 women took part. (no more details on recruitment methods available) |
| **Study design** | RCT |
| **Intervention(s) analysed** | Dietary counselling |
| **Nutrient** | Fat |
| **Methods/**  **Intervention details** | Women were randomised to intervention or control (60% intervention, 40% control) Half of the enrolees in Miami were to be Hispanic and half in Atlanta black, while those in Birmingham were to be representative of the metropolitan area by race and socioeconomic status. Nutritionists assigned personal fat gram goals to each participant and delivered intervention in group sessions that met weekly for 6 weeks, monthly for 9 months and then bi quarterly. Each session integrated nutritional and behavioural health strategies and encouraged food substitution. Group work included problem solving, peer support and role playing. Each participant selected specific changes that met her own needs and self-monitored her own fat intake. Several elements were tailored to suit the ethnic group and all forms/reading materials were of a 6^th^ grade reading level. Materials on good dietary practices including the Dietary Guidelines for Americans. (this is all the detail given for the control group) |
| **Length of follow up** | 18 months |
| **SEP measurement** | Ethnicity (black, Hispanic and white) |
| **Primary Outcomes SEP**  Mean change in daily intake of nutrients by ethnicity:   \| Variable \| 6 months \| 12 months \| 18 months \| \| --- \| --- \| --- \| --- \| \|  \| Intervention – control (CI) \| Intervention – control (CI) \| Intervention – control (CI) \| \| Fat % of energy (g) \| \| \| \| \| Black \| -10.78 (-12.27 to -9.29) \| -11.24 (-13.02 to -9.46) \| -10.33 (-13.15 to -7.51) \| \| Hispanic \| -5.65 (-8.51 to -2.79) \| -8.06 (-11.53 to -4.59) \| -11.61 (-18.49 to -4.73) \| \| White \| -11.89 (-12.97 to -10.81) \| -11.88 (-13.27 to -10.49) \| -12.11 (-13.85 to -10.37) \| | |
| **Secondary Outcomes SEC** | NA |
| **Primary Outcomes age/sex** | NA |
| **Secondary Outcomes age/sex** | NA |
| **Effect on inequalities** | **The mean change in fat % of energy consumed is the primary outcome of interest. The confidence intervals indicate that there is no significant difference in the change between the highest and lowest SEPs. This intervention has no impact on inequalities.** |
| **Study authors conclusions** | Our results suggest that the level of self-reported change observed in most other trials, approximately 10% of energy from fat, can be achieved in a large trial among postmenopausal women of diverse backgrounds. |
| **Limitations** | The intervention may seem to have been less effective among Hispanics due to the population of Hispanics which were recruited – there was complications with recruitment (slowing down and speeding up) due to hurricane Andrew in 1992, potentially enrolling a less select population, as suggested by lower levels of participation. Dropout rate. The intervention had no quantitative objective for goals other than total fat. Self-report. |
| **Notes** |  |

| Frenn M, Malin S, Bansal N, Delgado M, Greer Y, Havice M, Ho M, Schweizer H: **Addressing Health Disparities in Middle School Students' Nutrition and Exercise.** *Journal of Community Health Nursing* 2003, **20:**1-14 | |
| --- | --- |
| **Funder(s)** | Milwaukee Area Health Education Centre and the State of Wisconsin. |
| **“P”** | Person |
| **Aim(s)** | To examine the improvement related to the Health People 2010 objectives for low fat diet, moderate and vigorous physical activity from taking part in an interne and video intervention. |
| **Setting** | Two urban middle schools in the mid-west of the USA. |
| **Recruitment and sample size** | Entire classrooms of students were recruited to intervention or control groups based on classroom assignment to prevent diffusion of the intervention to the control group. Sample included 341 students; however, due to some students missing data, list wise deletion to include all variables in the analyses resulted in a sample of 67 in intervention and 63 in control. 41 eligible for free lunch, 15 had reduced fee and 68 had no reduction in fee. 58 African American, 47 white, 9 Asian, 4 Hispanic, 4 Native-American. Age ranged from 12-15; 56 boys, 68 girls. |
| **Study design** | Quasi experimental design |
| **Intervention(s) analysed** | Health education |
| **Nutrient** | Fat |
| **Methods/**  **Intervention details** | Based on Prochaska’s stages of change model. The intervention was conducted in 4 internet sessions plus a health snack session and a gym class (in one school only) of approximately 50 minutes (total of 6 sessions). Intervention was delivered in the classroom computer lab. The sessions covered topics such as identifying healthy foods in a food pyramid, identifying healthy alternative foods, the importance of exercise and a practical in which they prepared healthy snacks in groups. Participants completed surveys before and immediately after the intervention ended. |
| **Length of follow up** | Does not state – they do say however that the study was conducted during the school term in one year (2000-2001). |
| **SEP measurement** | Ethnicity |
| **Primary Outcomes SEP** | Differences in % fat in food for females only (n = 68). Pre-test levels were subtracted from post-test totals of % fat in food, so a lower number is the desired difference, 0 = no change in % fat.   \|  \| Intervention \| Control \| \| --- \| --- \| --- \| \| African American \| -0.53 \| 2 \| \| White \| 0.53 \| 1.73 \| \| Hispanic \| -5.3 \| 0 \| \| Asian \| 0.13 \| -0.8 \| \| Native American \| 0.53 \| 2.4 \| |
| **Secondary Outcomes SEP** | NA |
| **Primary Outcomes age/sex** | NA |
| **Secondary Outcomes age/sex** | NA |
| **Effect on inequalities** | **The difference in % fat from food consumed is the primary outcome of interest. Since there are no measurement errors or confidence intervals concerning this information, only a comparison of the magnitude of change between the lowest and highest SEP can be made. Lower SEP women decreased their % fat from food consumed while the highest SEP women increased their fat intake. This intervention reduces inequalities, however it cannot be inferred that this is a significant difference in the change between the groups.** |
| **Study authors conclusions** | The effect of the intervention varies from race to race and from boys to girls. Both the low fat diet and physical activity aspects of the intervention resulted in significantly better outcomes for all but one racial subgroup in girls, who have the greatest risk of obesity later in life. |
| **Limitations** | Missing data – the resulting usable sample did not permit analyses of both diet and exercise variables in the same equation. A larger study is warranted once additional sessions have been added to attain the 30% of fat in diet target in Healthy People 2010. There was a low test-retest correlation for the instruments used. Self-report. There were differences in access to healthy foods and exercise equipment between boys and girls and between levels of SEG that affected the participants responses in the surveys. |
| **Notes** |  |

| Kristal AR, Shattuck AL, Patterson RE: **Differences in fat-related dietary patterns between black, Hispanic and white women: results from the Women's Health Trial Feasibility Study in Minority Populations.** *Public Health Nutrition* 1999, **2:**253-262. | |
| --- | --- |
| **Funder(s)** | Not disclosed |
| **“P”** | Person |
| **Setting** | Health care clinics, Atlanta, Birmingham, Miami, USA |
| **Aim(s)** | One specific aim of the WHT:FSMP was to test whether a single nutrition intervention programme would be effective among women with markedly different culturally associated dietary patterns. The purpose of this paper is to better understand how the behavioural effects of the intervention differed across black, white and Hispanic participants. Specifically, it examines how sources of fat and patterns of fat-related dietary habits differed between racial and ethnic groups at baseline, and how dietary patterns in each racial and ethnic group changed as a result of the intervention. |
| **Recruitment and sample size** | Three clinical centres, 1702 women, mean age 60.2 years (SD 6.6), 60% white, 9% Hispanic, 31% black |
| **Study design** | RCT |
| **Intervention(s) analysed** | Dietary counselling |
| **Nutrient** | Fat |
| **Methods/**  **intervention details** | The nutrition intervention was delivered in group sessions led by centrally trained registered dieticians, which met weekly for 6 weeks, biweekly for 6 weeks, monthly for 9 months, and then quarterly. Sessions integrated both nutritional and behavioural topics, and consisted of problem-solving, role playing, sharing experiences, food tasting and didactic nutrition education. Each participant received a personal goal for fat intake based on height and estimated energy intake from the FFQ at baseline, which she monitored using a self-administered and scored ‘fat scan’. Participants selected the specific changes in food choices and food preparation methods that best fitted their own eating pattern, preferences and lifestyle. This report uses data from two dietary assessment instruments completed at baseline and 6 months post randomization. |
| **Length of follow up** | 6 months |
| **SEP measurement** | Ethnicity – black, white and Hispanic. White is described as having the highest income in the sample, (high SEP), with the black and Hispanic women having lower income (low SEP). |
| **Primary Outcomes SEP**  Mean fat intake (g) at baseline and effects of the WHT:FMSP at 6 months by ethnicity   \| Time point \| Atlanta and Birmingham \| \| Miami \| \| \| --- \| --- \| --- \| --- \| --- \| \| Black (n=422) \| White (n=718) \| Black (n=123) \| White (n=174) \| \| Total fat baseline \| 78.7 \| 82.3 \| 87.1 \| 82.3 \| \| Total fat intervention effect at 6 months \| -27 \| -29.4 \| -24.6 \| -26 \| \| % energy from fat baseline \| 39.8 \| 39.8 \| 38.4 \| 39.9 \| \| % energy from fat intervention effect at 6 months \| -11.3 \| -12.1 \| -5.9* \| -11 \|   *p = <0.05 vs white | |
| **Secondary Outcomes SEP** | Mean vegetable/salads intake (g) at baseline and effects of the WHT:FMSP at 6 months by ethnicity   \| Time point \| Atlanta and Birmingham \| \| Miami \| \| \| --- \| --- \| --- \| --- \| --- \| \| Black (n=422) \| White (n=718) \| Black (n=123) \| White (n=174) \| \| Vegetables/salads baseline \| 3.1* \| 3.6 \| 6.4** \| 3.1 \| \| Vegetables/salads intervention effect at 6 months \| -1.2 \| -1.2 \| -2.2 \| -0.9 \|   *p = <0.05 vs white; **p = <0.01 vs white |
| **Primary Outcomes age/sex** | NA |
| **Secondary Outcomes age/sex** | NA |
| **Effect on inequalities** | **The primary outcome of interest is the change in total fat intake between black and white women in the Atlanta and Birmingham sites as this has the largest sample. None of the results pertaining to the black women were significantly different to the white women. This intervention had no impact on inequalities.** |
| **Study authors conclusions** | A single dietary intervention programme can work well in culturally diverse groups. The intervention approach used in the WHT:FSMP was to educate participants about how to choose a low-fat diet and motivate them to do so, without prescribing specific foods or meal patterns. This approach may be effective for promoting long-term dietary change because it gives participants skills to select a personalized low-fat dietary plan. |
| **Limitations** | The results may not be generalizable to representative samples of black, Hispanic and white women. Participants in the WHT:FSMP had high fat intakes at baseline, were interested in nutrition and health, and were highly motivated to participate in nutrition research. In addition, most Hispanic participants were Cuban-Americans, and dietary patterns differ among Hispanic subgroups. The sample for the analyses reported here was a select group of WHT:FSMP participants who had completed both baseline and 6- month dietary assessments. The generalizability of Hispanic vs. white comparisons is weak, because only 36% of Hispanics had completed two valid FFQs. This was due, in part, to the disruption to Miami clinic operations caused by Hurricane Andrew in August 1992. |
| **Notes** |  |

| Reinaerts E, Nooijer Jd, Candel M, de Vries N: **Increasing children's fruit and vegetable consumption: distribution or a multicomponent programme?** *Public Health Nutrition* 2007, **10:**939-947 | |
| --- | --- |
| **Funder(s)** | The Netherlands Organisation for Health Research and development |
| **“P”** | Person |
| **Setting** | Primary schools in the Netherlands |
| **Aim(s)** | To test the effects on fruit and vegetable intake of a free F&V distribution and a multicomponent curriculum compared with a control group. |
| **Recruitment and sample size** | Schools were recruited by the Regional Health Service, which invited every school in the middle region of the province of Limburg, that had at least 200 students. Final sample of 12 primary schools, approx. 939 children aged 4–12 years |
| **Study design** | Controlled clinical trial |
| **Intervention(s) analysed** | Health education |
| **Nutrient** | Fruit and vegetables |
| **Methods/**  **intervention details** | There were two interventions. i) free F&V distribution at school. Every school day, all children were provided with one serving of fruit (twice a week), fruit juice (once a week) or raw vegetables (twice a week). The distributor ensured a large variety of F&V during the intervention year and ensured that the same kind of F&V was not served within 1 week. ii) a multicomponent school-based programme that consisted of a classroom curriculum and parental involvement. Children received a lunchbox that was especially designed to bring F&V to school undamaged. Classroom activities were developed in cooperation with school teachers and were adapted to the children’s age. Every 2 months, new activities were distributed among the teachers. By use of recurrent newsletters and homework activities taken home by the children, we tried to motivate parents to create a home environment that facilitates F&V consumption. Furthermore, posters with the project mascots were displayed at local supermarkets, serving as an environmental component aimed at reminding parents to buy F&V for their children. |
| **Length of follow up** | 12 months |
| **SEP measurement** | Children were classified as ‘native’ when both parents had been born in The Netherlands and as ‘non-native’ when one or both parents had been born outside The Netherlands. It is assumed that native children are high SEG and non-native children at low SEG. |
| **Primary Outcomes SEP**  Mean (sd) 24 hour fruit and vegetable juice consumption in times per day as a result of the multicomponent intervention   \| Ethnicity \| Baseline multicomponent programme \| Control \| Follow up multicomponent programme \| Control \| Regression coefficient \| Net effect \| \| --- \| --- \| --- \| --- \| --- \| --- \| --- \| \| Native \| 2.5 (1.2) \| 2.4 (1.1) \| 3 (1.2) \| 2.7 (1.2) \| 0.24* \| 0.2 \| \| Non-native \| 2.7 (1.3) \| 3.8 (1.5) \| 3.8 (1.4) \| 3.3 (1.8) \| 1.3** \| 1.6 \|   *p = <0.05, **p = <0.01 | |
| **Secondary Outcomes SEP** | NA |
| **Primary Outcomes age/sex**  Mean (sd) vegetable snack consumption in times per day as a result of the multicomponent intervention   \| Age /sex \| Baseline multicomponent programme \| Control \| Follow up multicomponent programme \| Control \| Regression coefficient \| Net effect \| \| --- \| --- \| --- \| --- \| --- \| --- \| --- \| \| 4-5 \| 0.2 (0.2) \| 0.3 (0.3) \| 0.3 (0.3) \| 0.3 (0.3) \| -0.01 \| 0.1 \| \| 6-8 \| 0.2 (0.2) \| 0.3 (0.2) \| 0.3 (0.3) \| 0.3 (0.3) \| 0.06* \| 0.1 \| \| 9-12 \| 0.2 (0.2) \| 0.3 (0.2) \| 0.3 (0.3) \| 0.2 (0.2) \| 0.03 \| 0.1 \| \| Boys \| 0.2 (0.2) \| 0.2 (0.3) \| 0.2 (0.3) \| 0.3 (0.2) \| 0 \| -0.1 \| \| Girls \| 0.2 (0.2) \| 0.3 (0.2) \| 0.3 (0.3) \| 0.3 (0.3) \| 0.07** \| 0.1 \|   *p = <0.05, **p = <0.01 | |
| **Secondary Outcomes age/sex** | NA |
| **Effect on inequalities** | **The change in fruit and vegetable juice consumption is the primary outcome of interest. Both groups of children significantly changed their fruit and vegetable juice consumption. 24-h fruit and vegetable juice consumption increased more in non-native children (beta coefficient =1.30, p = 0.01) than in native children (beta coefficient =0.24, p = 0.05). This intervention reduces inequalities.** |
| **Study authors conclusions** | It cannot simply be concluded that one of the two interventions was the most effective for all children, because both interventions showed different effects on children of different age, gender and ethnicity. However, when comparing both interventions, distributing fruit and vegetables was more effective: beside an increase in fruit and 24 hour fruit and vegetable juice consumption, the distribution programme was also effective in increasing vegetable consumption for different subgroups. |
| **Limitations** | As the authors were interested in the effects on all children (aged 4–12 years), they had to use self-reported data from parents, since the youngest children were not able to fill in questionnaires. The measures we used were not validated among the target population. Schools were not randomly assigned to the intervention or control groups, but were matched based on school size and ethnicity. Baseline analyses, however, showed that schools were comparable except for ethnicity and baseline consumption levels. These were therefore included as covariates in all analyses. |
| **Notes** |  |

| Webber LS, Osganian SK, Feldman HA, Wu M, McKenzie TL, Nichaman M, Lytle LA, Edmundson E, Cutler J, Nader PR, Luepker RV: **Cardiovascular Risk Factors among Children after a 212-Year Intervention—The CATCH Study.** *Preventive Medicine* 1996, **25:**432-441 | |
| --- | --- |
| **Funder(s)** | National Heart Lung and Blood Institute of the US Public Health Service |
| **“P”** | Place |
| **Aim(s)** | The Child and Adolescent Trial for Cardiovascular Health (CATCH)’s main physiological goal was to reduce serum total cholesterol levels. Behavioural goals included the reduction of dietary fat (total, saturated) and sodium intake, increased physical activity and the prevention of the onset of smoking. |
| **Setting** | Secondary schools in the US |
| **Recruitment and sample size** | Recruited for the study with extensive negotiations and presentations with school district staff, principals and teachers; a formal contract to 3 years of intervention and evaluation activities that was signed by the school district administrators and the principal investigators. 4019 children from four US states were measured for this analysis after 2.5 years. |
| **Study design** | RCT |
| **Intervention(s) analysed** | School based intervention |
| **Nutrient** | Fat, salt |
| **Methods/**  **Intervention details** | CATCH consisted of the Eat Smart school food service program, CATCH PE (physical education), classroom curricula, and parental involvement programs. Eat Smart was a school-level intervention designed to incorporate the current dietary recommendations into the schools' food service program. The goal was to provide children with reduced fat, saturated fat, and sodium in meals while maintaining recommended levels of essential nutrients and calories, as well as maintaining school meal participation. Food providers and all staff were also trained by dieticians concerning food choices. CATCH PE was designed to increase the amount of time students spent in enjoyable moderate-to-vigorous physical activity (MVPA) at school during PE, as well as to teach students appropriate activities for other times of the day that could be maintained throughout life. The third- through fifth-grade classroom curricula were developed in order for students to be able to identify and choose lower fat and lower sodium foods and to be more physically active. In addition, fifth grade included a tobacco use prevention curriculum with the goal to change students’ intentions to use tobacco. CATCH used two primary strategies for family involvement. The first strategy, home curricula involved skill-building activity packets that complemented the four classroom curricula and that the third- through fifth-grade students brought home to complete with their parents. There were also activity nights at the school that both the parents and children attended. |
| **Length of follow up** | This study examined the intervention effects of the study 2.5 years in to the intervention. |
| **SEP measurement** | The authors measure ethnicity (Caucasian, African-American, Native American, Asian/Pacific and other). It should be noted that they do not infer any SEG with any of these groups. |
| **Primary Outcomes SEP**  Serum total cholesterol levels (mg/d) among children from third to fifth grade by ethnicity:   \|  \| Caucasian \| \| Afric-Amer \| \| Hispanic \| \| Nativ-Amer \| \| Asian/Pacific \| \| Other \| \| \| --- \| --- \| --- \| --- \| --- \| --- \| --- \| --- \| --- \| --- \| --- \| --- \| --- \| \|  \| 3rd \| 5th \| 3rd \| 5th \| 3rd \| 5th \| 3rd \| 5th \| 3rd \| 5^th^ \| 3rd \| 5th \| \| Girl interv \| 171 \| 167 \| 172 \| 165 \| 166 \| 164 \| 173 \| 170 \| 173 \| 177 \| 171 \| 176 \| \| Girl control \| 173 \| 170 \| 175 \| 169 \| 167 \| 165 \| 166 \| 155 \| 177 \| 173 \| 167 \| 168 \| \| Boy interv \| 166 \| 167 \| 171 \| 170 \| 172 \| 170 \| 170 \| 175 \| 170 \| 171 \| 171 \| 169 \| \| Boy control \| 167 \| 168 \| 170 \| 173 \| 165 \| 170 \| 167 \| 166 \| 166 \| 170 \| 166 \| 171 \|   Results taken and measured from very small and unclear graphs therefore some measurement error needs to be considered. Also, graphs have standard error bars but these were simply too small to measure accurately. However, these Se bars were assessed for overlap. | |
| **Secondary Outcomes SEP**  HDL cholesterol levels (mg/d) among children from third to fifth grade by ethnicity:   \|  \| Caucasian \| \| Afric-Amer \| \| Hispanic \| \| Nativ-Amer \| \| Asian/Pacific \| \| Other \| \| \| --- \| --- \| --- \| --- \| --- \| --- \| --- \| --- \| --- \| --- \| --- \| --- \| --- \| \|  \| 3rd \| 5th \| 3rd \| 5th \| 3rd \| 5th \| 3rd \| 5th \| 3rd \| 5^th^ \| 3rd \| 5th \| \| Girl interv \| 51 \| 48 \| 54 \| 50 \| 51 \| 47 \| 50 \| 45 \| 48 \| 49 \| 45 \| 43 \| \| Girl control \| 51 \| 48 \| 55 \| 50 \| 50 \| 47 \| 59 \| 50 \| 50 \| 46 \| 45 \| 43 \| \| Boy interv \| 51 \| 48 \| 58 \| 54 \| 54 \| 50 \| 52 \| 53 \| 51 \| 51 \| 46 \| 50 \| \| Boy control \| 51 \| 50 \| 52 \| 51 \| 51 \| 48 \| 43 \| 47 \| 51 \| 50 \| 48 \| 47 \|   Results taken and measured from very small and unclear graphs therefore some measurement error needs to be considered. Also, graphs have standard error bars but these were simply too small to measure accurately. | |
| **Primary Outcomes age/sex** | NA |
| **Secondary Outcomes age/sex** | NA |
| **Effect on inequalities** | **The change in serum cholesterol for boys is the primary outcome of interest. From examining the standard error bars on the graphs displaying this information, there was substantial overlap relating to both SEP results. This intervention had no impact on inequalities.** |
| **Study authors conclusions** | CATCH positively affected the school environment and the health behaviours of children. Although these changes did not translate to significant changes in risk factors at these ages, they have the potential for promoting positive health behaviours into adulthood. In addition, the differential effects noted by gender and ethnicity add to the body of literature on the epidemiology risk factors during childhood and may enhance the development of more specific intervention programs. |
| **Limitations** | The dose (one to two sessions of 40 mins per week for 12-15 weeks) and the duration (2.5 years) of the intervention were minimal. The change in serum cholesterol was modest. This is probably due to the fact that the home environment was not targeted in this intervention, and this is undoubtedly where children consume most of their food. The onset of puberty in this age group is variable, which may mask intervention effects. Self-report. |
| **Notes** |  |

| Whetstone LM, Kolasa KM, Collier DN: **Participation in Community-Originated Interventions Is Associated With Positive Changes in Weight Status and Health Behaviors in Youth.** *American Journal of Health Promotion* 2012, **27:**10-16 | |
| --- | --- |
| **Funder(s)** | North Carolina Health and Wellness Trust fund commission (NCHWTF) |
| **“P”** | Place |
| **Setting** | Communities in North Carolina, USA |
| **Aim(s)** | To conduct an independent evaluation of the NCHWTF grant program to assess changes in children's health behaviours and weight status after participating in community-originated projects |
| **Sample size** | 1144 individuals aged 4.1 – 18.6 years (mean = 9.5 years). 51.7% female, 64.7% Caucasian, 35.3% African American, 2.6% Hispanic/Latino origin. |
| **Recruitment and study design** | Prospective cohort study |
| **Intervention(s) analysed** | Area based intervention |
| **Nutrient** | Not specified |
| **Methods /intervention details** | The NCHWTF were running an existing childhood obesity grant programme which included multiple interventions. These included physical activity and nutrition interventions. Most projects were school based. 10 were school based only, 2 were after school based only, one was faith based only, one was community based only, 4 were school and after school based, and one was after school and community based. The authors therefore decided to follow the progress of some children in these interventions. Each site was given the instruction of collecting a cohort of children who was exposed to at least one program activity and who were representative of all children involved. Only recorded baseline and final measurements. |
| **Length of follow up** | 20 months |
| **SEP measurement** | ethnicity |
| **Primary Outcomes SEP** | Baseline z scores and final z scores (standard deviation) by different weight status’ and race   \| Race \| No. (%) \| Baseline \| Final \| Change in z score \| \| --- \| --- \| --- \| --- \| --- \| \| African American \| 404 (35) \| 1.13 (1.15) \| 1.02 (1.24) \| -0.1023 (0.57367) \| \| Caucasian \| 740 (65) \| 0.66 (1.09) \| 0.66 (1.08) \| -0.0007 (0.54635) \| |
| **Secondary Outcomes SEP** | NA |
| **Primary Outcomes age/sex** | Baseline z scores ad final z scores (standard deviation) by different weight status’ and gender   \| Gender \| No. (%) \| Baseline \| Final \| \| --- \| --- \| --- \| --- \| \| Total \| \| \| \| \| Female \| 592 (52) \| 0.83 (1.15) \| 1.02 (1.24) \| \| Male \| 552 (48) \| 0.81 (1.12) \| 0.66 (1.08) \| |
| **Secondary Outcomes age/sex** | NA |
| **Effect on inequalities** | **The change in BMI z score is the primary outcome of interest. The standard deviations for the change in z score reveal no significant difference between the highest and lowest SEP. This intervention had no impact on inequalities.** |
| **Study authors conclusions** | A global approach to childhood obesity prevention and treatment may have small but positive influences on target health behaviours. Found a surprisingly large positive change in weight status for overweight and obese children who participated in these community originated interventions |
| **Limitations** | Baseline scores are very different, with the African American sample having a higher baseline BMI z score – makes comparisons v difficult and an opinion on inequalities biased. The authors have no indication of the level of control that was applied to all of the interventions as they did not run them. Did not randomise participants to be included in the cohort. Specific programme activities cannot be linked to individuals. Cannot determine how much of the effect is due to the physical activity interventions. |
| **Notes** |  |

| Willi SM, Hirst K, Jago R, Buse J, Kaufman F, El ghormli L, Bassin S, Elliot D, Hale DE: **Cardiovascular risk factors in multi‐ethnic middle school students: The HEALTHY primary prevention trial.** *Pediatric Obesity* 2012, **7:**230-239 | |
| --- | --- |
| **Funder(s)** | The National Institute of Diabetes and Digestive and Kidney Diseases; The National Institute of Health and additional support from the American Diabetes Association |
| **“P”** | Place |
| **Aim(s)** | To examine the effects of an integrated, multi-component school-based intervention programme on CVD risk factors among a multi-ethnic cohort of middle school students |
| **Setting** | 42 Middle schools in Philadelphia, USA |
| **Recruitment and sample size** | 42 middle schools with at least 50% of students eligible for free or reduced price lunch or belonging to a minority group were recruited by 7 participating centres. Total sample = 4363 participants (2062 male) |
| **Study design** | RCT |
| **Intervention(s) analysed** | School based intervention |
| **Nutrient** | Not specified |
| **Methods/**  **Intervention details** | Intervention had 4 parts: i) Nutritional intervention – attempted to change the nutritional quality of foods offered in the cafeteria, as snacks after school and in vending machines (details not presented in this or supplemental referenced paper). This was supported with taste tests and cafeteria educational sessions to encourage children to try new foods. ii) PE – Brought in new equipment and lesson plans with training for PE teachers to increase the time children did PE. iii) Behaviour intervention – classroom activities, individual and group work, goal setting, self-monitoring and problem solving. iv) Communications integration – promoted change in nutrition, activity and behaviour through messages, images, events and activities. Children did a full health screening at baseline (6^th^ grade) and at the end of the study (8^th^ grade). Half the schools randomly assigned to intervention group. |
| **Length of follow up** | 2.5 years |
| **SEP measurement** | ethnicity |
| **Primary Outcomes SEP** | Descriptive statistics for CVD risk factors at baseline (6^th^ grade) and end of study (8^th^ grade) – intervention schools in mean (SD) and %   \| CVD risk factor \| Hispanic \| \| Non-Hispanic black \| \| Non-Hispanic white \| \| \| --- \| --- \| --- \| --- \| --- \| --- \| --- \| \| BP: \| 6th \| 8th \| 6th \| 8th \| 6th \| 8th \| \| Normal \| 84.4% \| 82.4% \| 77.9% \| 86.6% \| 84.3% \| 91.4% \| \| Pre-HTN \| 8.6% \| 8.3% \| 10.3% \| 6.2% \| 3.8% \| 3% \| \| HTN stage 1 \| 8.6% \| 7.7% \| 8.9% \| 5.2% \| 8.9% \| 4.6% \| \| HTN stage 2 \| 5.5% \| 1.7% \| 3% \| 2.1% \| 3% \| 1.1% \| \| Total cholesterol (mm0 L^-1^) \| 4 (0.7) \| 3.8 (0.7) \| 4.2 (0.7) \| 3.9 (0.7) \| 4.1 (0.7) \| 3.8 (0.7) \|   Descriptive statistics for CVD risk factors at baseline (6^th^ grade) and end of study (8^th^ grade) – control schools in mean (SD) and %   \| CVD risk factor \| Hispanic \| \| Non-Hispanic black \| \| Non-Hispanic white \| \| \| --- \| --- \| --- \| --- \| --- \| --- \| --- \| \| BP: \| 6th \| 8th \| 6th \| 8th \| 6th \| 8th \| \| Normal \| 82.9% \| 83.4% \| 78.2% \| 78.2% \| 78.1% \| 84.4% \| \| Pre-HTN \| 7.9% \| 7.6% \| 9.7% \| 8.8% \| 9.3% \| 7.4% \| \| HTN stage 1 \| 6.7% \| 7% \| 8.5% \| 10% \| 9.1% \| 6.5% \| \| HTN stage 2 \| 2.3% \| 2% \| 3.6% \| 3% \| 3.6% \| 1.7% \| \| Total cholesterol (mm0 L^-1^) \| 4 (0.7) \| 3.8 (0.7) \| 4.3 (0.7) \| 4 (0.7) \| 4.1 (0.7) \| 3.8 (0.7) \| |
| **Secondary Outcomes SEP** | NA |
| **Primary Outcomes age/sex** | Descriptive statistics for CVD risk factors at baseline (6^th^ grade) and end of study (8^th^ grade) – intervention schools in mean (SD) and %   \| CVD risk factor \| Male \| \| Female \| \| \| --- \| --- \| --- \| --- \| --- \| \| BP: \| 6th \| 8th \| 6th \| 8th \| \| Normal \| 83% \| 82.4% \| 82.3% \| 87.4% \| \| Pre-HTN \| 7.8% \| 7.3% \| 8.8% \| 6.5% \| \| HTN stage 1 \| 7.4% \| 7.7% \| 6.4% \| 5.2% \| \| HTN stage 2 \| 1.7% \| 2.6% \| 2.5% \| 0.9% \| \| Total cholesterol (mm0 L^-1^) \| 4.3 (0.7) \| 3.8 (0.7) \| 4 (0.7) \| 3.9 (0.7) \|   Descriptive statistics for CVD risk factors at baseline (6^th^ grade) and end of study (8^th^ grade) – control schools in mean (SD) and %   \| CVD risk factor \| Male \| \| Female \| \| \| --- \| --- \| --- \| --- \| --- \| \| BP: \| 6th \| 8th \| 6th \| 8th \| \| Normal \| 78.1% \| 78.4% \| 84.4% \| 87% \| \| Pre-HTN \| 11.2% \| 9.5% \| 6% \| 6.4% \| \| HTN stage 1 \| 8.5% \| 9% \| 6.2% \| 5.6% \| \| HTN stage 2 \| 2.1% \| 3.1% \| 3.5% \| 1% \| \| Total cholesterol (mm0 L^-1^) \| 4.3 (0.7) \| 3.8 (0.7) \| 4 (0.7) \| 3.9 (0.7) \| |
| **Secondary Outcomes age/sex** | NA |
| **Effect on inequalities** | The change in total cholesterol is the primary outcome of interest. The authors state that there were no significant t differences in change of total cholesterol between the ethnic groups. Therefore this intervention had no impact on inequalities. |
| **Study authors conclusions** | **The total cholesterol at the end of the intervention is the primary outcome of interest. The standard deviations indicate that there is no difference in this value between the lowest and highest SEP. This intervention had no impact on inequalities.** |
| **Limitations** | About one half of the students were overweight or obese at baseline – this is supposedly a preventive strategy and should have been aimed at the normal population. The intervention only involved school time and therefore only a third of the students time. Family support was not evaluated. Multicomponent and cannot determine which part of the intervention was effective. May have had an effect on sodium levels but this was not measured. |
| **Notes** |  |
