# Supplementary material for: Are interventions to promote healthy eating equally effective for all? Systematic review of socioeconomic inequalities in impact
Source: BMC Public Health. 2015 May 2;15:457. doi: 10.1186/s12889-015-1781-7 (PMC4423493; doi:10.1186/s12889-015-1781-7)
Supplement: Additional file 4: — Sensitivity analysis. [file 12889_2015_1781_MOESM4_ESM.docx]

**Additional file 4 – Sensitivity analysis**

We conducted a sensitivity analysis to determine if the general trends seen in the Harvest plot would change if we had been more or less selective in our screening process.

The first sensitivity analysis included only the studies which gave indicators of significance concerning the quantitative data that they split by SEG (p values, standard error, confidence intervals or standard deviation)[[1-28](#_ENREF_1)]. The removal of interventions that provided no indicators of statistical significance concerning outcome has no impact on our main findings.

**Harvest plot summarising the effects of only the healthy eating interventions that indicated levels of significance concerning the quantitative effect on inequalities**


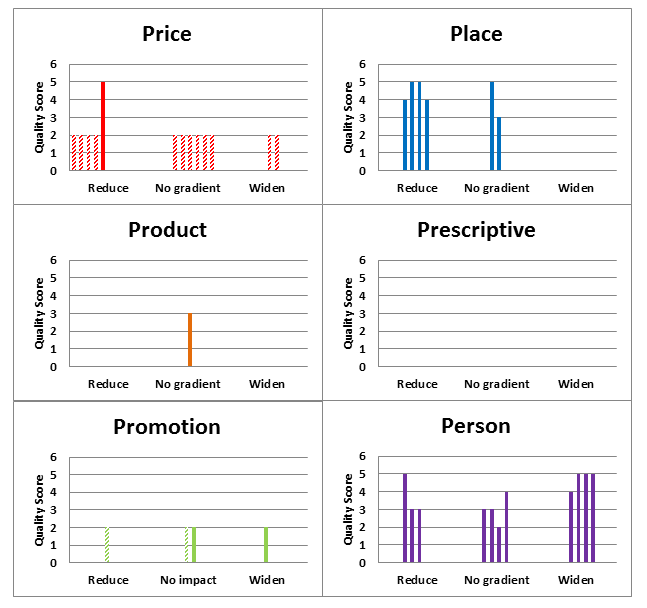


*Each matrix within the Harvest plot ‘supermatrix’ illustrates our findings for each “P”. Each matrix consists of three columns indicating whether inequalities were reduced, widened or showed no change. Each bar represents one intervention. The height of the bar indicates the quality score of the study graded out of 6. Modelling studies are indicated by patterned bars.*

The second sensitivity analysis was more inclusive and included those studies that split their findings quantitatively by ethnicity, but did not indicate that this was a measure of SEG, and therefore could have been a cultural measure[[29-36](#_ENREF_29)].

**Harvest plot summarising the effects of healthy eating intervention on inequalities including those who split their effects by ethnicity without indicating a socioeconomic parameter**


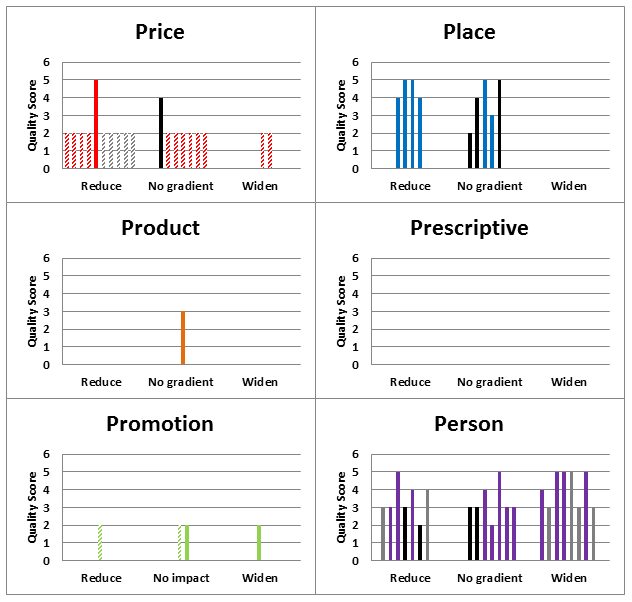


*Each matrix within the Harvest plot ‘supermatrix’ illustrates our findings for each “P”. Each consists of three columns indicating whether inequalities were reduced, widened or showed no gradient. Each bar represents one intervention. The height of the bar indicates the quality score of the study graded out of 6. Grey bars indicate interventions with no significance values given concerning the difference in effect of the intervention on SEG. Black bars are additional interventions that only examined ethnicity. Modelling studies are indicated by patterned bars*.

Eight studies were identified as having only split their outcome quantitatively by ethnicity (1 “Price[[29](#_ENREF_29)], 3 “Place”[[34-36](#_ENREF_34)], 2 “Promotion”[[31](#_ENREF_31), [33](#_ENREF_33)] and 2 “Personalised”[[30](#_ENREF_30), [32](#_ENREF_32)]). The addition of these studies does not alter the finding that “Price” interventions are the most likely to reduce inequalities and “Personalised” interventions the most likely to widen inequalities.

References

1. Bürgi F, Niederer I, Schindler C, Bodenmann P, Marques-Vidal P, Kriemler S, Puder JJ: **Effect of a lifestyle intervention on adiposity and fitness in socially disadvantaged subgroups of preschoolers: A cluster-randomized trial (Ballabeina).** *Preventive Medicine* 2012, **54:**335-340.

2. Campbell MK, Demark-Wahnefried W, Symons M, Kalsbeek WD, Dodds J, Cowan A, Jackson B, Motsinger B, Hoben K, Lashley J, et al: **Fruit and vegetable consumption and prevention of cancer: the Black Churches United for Better Health project.** *American Journal of Public Health* 1999, **89:**1390-1396.

3. Capacci S, Mazzocchi M: **Five-a-day, a price to pay: An evaluation of the UK program impact accounting for market forces.** *Journal of Health Economics* 2011, **30:**87-98.

4. Carcaise-Edinboro P, McClish D, Kracen AC, Bowen D, Fries E: **Fruit and vegetable dietary behavior in response to a low-intensity dietary intervention: the rural physician cancer prevention project.** *Journal of Rural Health*, **24:**299-305.

5. Cash SB, Sunding DL, Zilberman D: **Fat taxes and thin subsidies: Prices, diet, and health outcomes.** *Food Economics - Acta Agriculturae Scandinavica, Section C* 2005, **2:**167-174.

6. Curtis PJ, Adamson AJ, Mathers JC: **Effects on nutrient intake of a family-based intervention to promote increased consumption of low-fat starchy foods through education, cooking skills and personalised goal setting: the Family Food and Health Project.** *British Journal of Nutrition*, **107:**1833-1844.

7. Dallongeville J, Dauchet L, Mouzon Od, Réquillart V, Soler L-G: **Increasing fruit and vegetable consumption: a cost-effectiveness analysis of public policies.** *The European Journal of Public Health* 2011, **21:**69-73.

8. Estaquio C, Druesne-Pecollo N, Latino-Martel P, Dauchet L, Hercberg S, Bertrais S: **Socioeconomic differences in fruit and vegetable consumption among middle-aged French adults: adherence to the 5 A Day recommendation.** *Journal of the American Dietetic Association*, **108:**2021-2030.

9. Finkelstein Ea ZC: **IMpact of targeted beverage taxes on higher- and lower-income households.** *Archives of Internal Medicine* 2010, **170:**2028-2034.

10. Friel S, Kelleher C, Campbell P, Nolan G: **Evaluation of the Nutrition Education at Primary School (NEAPS) programme.** *Public Health Nutrition*, **2:**549-555.

11. Haerens L, Deforche B, Maes L, Brug J, Vandelanotte C, De Bourdeaudhuij I: **A computer-tailored dietary fat intake intervention for adolescents: Results of a randomized controlled trial.** *Annals of Behavioral Medicine* 2007, **34:**253-262.

12. Havas S, Anliker J, Damron D, Langenberg P, Ballesteros M, Feldman R: **Final results of the Maryland WIC 5-A-Day Promotion Program.** *American Journal of Public Health* 1998, **88:**1161-1167.

13. Havas S, Anliker J, Greenberg D, Block G, Block T, Blik C, Langenberg P, DiClemente C: **Final results of the Maryland WIC food for life program.** *Preventive Medicine* 2003, **37:**406-416.

14. Hughes RJ, Edwards KL, Clarke GP, Evans CEL, Cade JE, Ransley JK: **Childhood consumption of fruit and vegetables across England: a study of 2306 6–7-year-olds in 2007.** *British Journal of Nutrition* 2012, **108:**733-742.

15. Jeffery RW, French SA: **Preventing weight gain in adults: design, methods and one year results from the Pound of Prevention study.** *International Journal of Obesity and Related Metabolic Disorders: Journal of the International Association for the Study of Obesity* 1997, **21:**457-464.

16. Jouret B, Ahluwalia N, Dupuy M, Cristini C, Nègre-Pages L, Grandjean H, Tauber M: **Prevention of overweight in preschool children: results of kindergarten-based interventions.** *International Journal of Obesity* 2009, **33:**1075-1083.

17. Millett C, Laverty AA, Stylianou N, Bibbins-Domingo K, Pape UJ: **Impacts of a national strategy to reduce population salt intake in England: serial cross sectional study.** *PLoS ONE,* 2012, **7(1)**.

18. Nederkoorn C, Havermans RC, Giesen JCAH, Jansen A: **High tax on high energy dense foods and its effects on the purchase of calories in a supermarket. An experiment.** *Appetite*, **56:**760-765.

19. Nnoaham KE, Sacks G, Rayner M, Mytton O, Gray A: **Modelling income group differences in the health and economic impacts of targeted food taxes and subsidies.** *International Journal of Epidemiology*, **38:**1324-1333.

20. Plachta-Danielzik S, Pust S, Asbeck I, Czerwinski-Mast M, Langnase K, Fischer C, Bosy-Westphal A, Kriwy P, Muller MJ: **Four-year follow-up of school-based intervention on overweight children: the KOPS study.** *Obesity*, **15:**3159-3169.

21. Reynolds KD, Franklin FA, Binkley D, Raczynski JM, Harrington KF, Kirk KA, Person S: **Increasing the Fruit and Vegetable Consumption of Fourth-Graders: Results from the High 5 Project.** *Preventive Medicine* 2000, **30:**309-319.

22. Rush E, Reed P, McLennan S, Coppinger T, Simmons D, Graham D: **A school-based obesity control programme: Project Energize. Two-year outcomes.** *British Journal of Nutrition*, **107:**581-587.

23. Sharma A, Hauck K, Hollingsworth B, Siciliani L: **The Effect of Taxing Sugar-Sweetened Beverages across different Income Groups: A Censored Demand Approach.** *Journal of Economic Literature* in press.

24. Sorensen G, Linnan L, Hunt MK: **Worksite-based research and initiatives to increase fruit and vegetable consumption.** *Preventive Medicine: An International Journal Devoted to Practice and Theory* 2004, **39:**S94-S100.

25. Sorensen G, Stoddard A, Hunt MK, Hebert JR, Ockene JK, Avrunin JS, Himmelstein J, Hammond SK: **The effects of a health promotion-health protection intervention on behavior change: the WellWorks Study.** *American Journal of Public Health* 1998, **88:**1685-1690.

26. Stables GJ, Subar AF, Patterson BH, Dodd K, Heimendinger J, Van Duyn MAS, Nebeling L: **Changes in vegetable and fruit consumption and awareness among US adults: Results of the 1991 and 1997 5 A Day for Better Health Program surveys.** *Journal of the American Dietetic Association* 2002, **102:**809-817.

27. Toft U, Jakobsen M, Aadahl M, Pisinger C, Jørgensen T: **Does a population-based multi-factorial lifestyle intervention increase social inequality in dietary habits? The Inter99 study.** *Preventive Medicine: An International Journal Devoted to Practice and Theory* 2012, **54:**88-93.

28. Wendel-Vos GCW, Dutman AE, Verschuren WMM, Ronckers ET, Ament A, van Assema P, van Ree J, Ruland EC, Schuit AJ: **Lifestyle Factors of a Five-Year Community-Intervention Program: The Hartslag Limburg Intervention.** *American Journal of Preventive Medicine* 2009, **37:**50-56.

29. Blakely T, Ni Mhurchu C, Jiang Y, Matoe L, Funaki-Tahifote M, Eyles HC, Foster RH, McKenzie S, Rodgers A: **Do effects of price discounts and nutrition education on food purchases vary by ethnicity, income and education? Results from a randomised, controlled trial.** *Journal of Epidemiology and Community Health* 2011, **65:**902-908.

30. Coates RJ, Bowen DJ, Kristal AR, Feng Z, Oberman A, Hall WD, George V, Lewis CE, Kestin M, Davis M, et al: **The Women's Health Trial Feasibility Study in Minority Populations: Changes in Dietary Intakes.** *American Journal of Epidemiology* 1999, **149:**1104-1112.

31. Frenn M, Malin S, Bansal N, Delgado M, Greer Y, Havice M, Ho M, Schweizer H: **Addressing Health Disparities in Middle School Students' Nutrition and Exercise.** *Journal of Community Health Nursing* 2003, **20:**1-14.

32. Kristal AR, Shattuck AL, Patterson RE: **Differences in fat-related dietary patterns between black, Hispanic and white women: results from the Women's Health Trial Feasibility Study in Minority Populations.** *Public Health Nutrition* 1999, **2:**253-262.

33. Reinaerts E, Nooijer Jd, Candel M, de Vries N: **Increasing children's fruit and vegetable consumption: distribution or a multicomponent programme?** *Public Health Nutrition* 2007, **10:**939-947.

34. Webber LS, Osganian SK, Feldman HA, Wu M, McKenzie TL, Nichaman M, Lytle LA, Edmundson E, Cutler J, Nader PR, Luepker RV: **Cardiovascular Risk Factors among Children after a 212-Year Intervention—The CATCH Study.** *Preventive Medicine* 1996, **25:**432-441.

35. Whetstone LM, Kolasa KM, Collier DN: **Participation in Community-Originated Interventions Is Associated With Positive Changes in Weight Status and Health Behaviors in Youth.** *American Journal of Health Promotion* 2012, **27:**10-16.

36. Willi SM, Hirst K, Jago R, Buse J, Kaufman F, El ghormli L, Bassin S, Elliot D, Hale DE: **Cardiovascular risk factors in multi‐ethnic middle school students: The HEALTHY primary prevention trial.** *Pediatric Obesity* 2012, **7:**230-239.
