# Supplementary material for: Are interventions to promote healthy eating equally effective for all? Systematic review of socioeconomic inequalities in impact
Source: BMC Public Health. 2015 May 2;15:457. doi: 10.1186/s12889-015-1781-7 (PMC4423493; doi:10.1186/s12889-015-1781-7)
Supplement: Additional file 5: — Summary of included studies (categorised using the 6Ps framework). [file 12889_2015_1781_MOESM5_ESM.docx]

| **Author**  **Additional file 5 – Summary of included studies** (categorised using the 6Ps framework) | **Study design** | **Sample/**  **Setting** | **Intervention** | **Quality^∆^** | **Outcome measured** | **SEP measurement** | **Amount of change by SEP#** | **Effect on SEP inequalities†** |
| --- | --- | --- | --- | --- | --- | --- | --- | --- |
| ***Price*** | | | | | | | | |
| **Allais**^33^ | Modelling study | 5000 households France | Tax on high energy density food: Modelled a 10% tax on high calorie food | 2 | Change in fat consumed (%) | Household income | Low = -3.207  High = -3.058 | **↓** |
| **Cash^39^** | Modelling study | 18,081 individuals over 2 years old USA | Subsidy on fruit and vegetables: Modelled a 1% price decrease in fruit and vegetables | 2 | CHD incidence | Household income | Low = -1152 (SE = 64.03)  High = -3492 (SE = 104.58) | **↑*** |
| **Dallongeville**[**^34^**](#_ENREF_31) | Modelling study | 2,624 adults and 1,455 children France | Subsidy on fruit and vegetables: Modelled a reduction of tax on fruit & vegetables from 5.5% to 2.1% | 2 | Change in mean fruit and vegetable consumption (g/d) | Household income | Low = 3.4 (CI = 1.2-7.5)  Rest = 5 (CI = 3.1-7.6) | **↔*** |
|  |  |  | Subsidy on fruit and vegetables: Modelled a €100 per year per person food stamp programme for fruit and vegetables |  |  |  | Low = 7 (CI =6-9.2)  Rest = -0.3 (CI = -0.5- -0.2) | **↓*** |
| **Finkelstein^40^** | Modelling study | 384,252 households Canada | Tax on high energy density food: Modelled a 20% tax on carbonated sugar sweetened beverages | 2 | Mean change in energy intake from all beverages | Household income | Low = +0.2 (SE = +4 to -4)  High = +1 (SE = +4 to -2) | **↔*** |
|  |  |  | Tax on high energy density food: Modelled a 40% tax on carbonated sugar sweetened beverages |  |  |  | Low = -0.2 (SE = +5.2 to -6)  High = +1 (SE = +6 to -5) | **↔*** |
|  |  |  | Tax on high energy density food: Modelled a 20% tax on all sugar sweetened beverages |  |  |  | Low = -2.5 (SE = +2.5 to -8)  High = +2 (SE = -5 to +2) | **↔*** |
|  |  |  | Tax on high energy density food: Modelled a 40% tax on all sugar sweetened beverages |  |  |  | Low = -5 (SE = +4 to -14)  High = +2.5 (SE = +8 to -4) | **↔*** |
| **Nederkoorn^35^** | RCT | 306 adults  Holland | Tax on high energy density food: Internet supermarket task where all products with a caloric value of more than 300 kcal/100 g were taxed by 50% | 5 | % change in calories purchased in lean individuals | Food budget | Low = -38.5  High = -2.8 | **↓*** |
| **Nnoaham**[**^36^**](#_ENREF_34) | Modelling study | UK | Tax on high energy density food: Modelled a 17.5% VAT on foods that are a major source of saturated fats | 2 | % change in calorie intake | Household income | Low = -0.53 (CI = -0.65, -0.41)  High = -0.59 (CI = -0.73, -0.46) | **↔*** |
|  |  |  | Tax on high energy density food: Modelled a 17.5% VAT on foods that are classified as ‘less healthy’ by nutrient profiling |  |  |  | Low = -3.08 (CI = -3.38, -2.78)  High = -2.02 (CI = -2.28, -1.77) | **↓*** |
|  |  |  | Combination of tax and subsidy: Combined the taxation on ‘less healthy’ foods with a 17.5% subsidy on fruit and vegetables |  |  |  | Low = -1.53 (CI= -1.74, -1.32)  High = -0.58 (CI = -0.72, -0.45) | **↓*** |
|  |  |  | Combination of tax and subsidy: Combined the taxation on ‘less healthy’ foods with a 32.5% subsidy on fruit and vegetables to neutralise the tax revenue |  |  |  | Low = -0.21 (CI = -0.28, -0.13)  High = 0.65 (CI = 0.51, 0.79) | **↓*** |
| **Sharma**^41^ | Modelling study | 1,390 households  Australia | Tax on high energy density food: Modelled a 20% tax on sugar sweetened beverages | 2 | Mean net change in body weight in kg | Household income | Low = -0.482  High = -0.541 | **↑*** |
| **Smed**^37^ | Modelling study | 2000 households  Denmark | Combination of tax and subsidy: Modelled a 5% tax on fatty meat and dairy products alongside subsidies on fruit and vegetables, potatoes and grain products scaled to give a revenue neutral scenario | 2 | Change in nutrient demand of saturated fat (%) | Social class | Low = -10.4  High = -0.3 | **↓** |
|  |  |  | Combination of tax and subsidy: Modelled a 7.89 DKK/kg tax on saturated fats alongside subsidies on fibres which were scaled to give a revenue neutral scenario |  |  |  | Low = -22.5  High = +39 | **↓** |
|  |  |  | Combination of tax and subsidy: Modelled a 7.89 DKK/kg tax on saturated fats alongside subsidies on fibres which were scaled to give a revenue neutral scenario with an additional 10.3 DKK/kg tax on sugar |  |  |  | Low = -13.4  High = +52.2 | **↓** |
| **Tiffin**^38^ | Modelling study | 6,760 households  UK | Combination of tax and subsidy: Modelled a tax that increases the price of fatty foods by 1% for every per cent of saturated fats they contain. This was capped at 15%, with a matching subsidy on fruit and vegetables to cancel the cost of the fat tax. | 2 | % change in energy intake | Occupation | Low = -2  High = -1.34 | **↓** |
| ***Place*** | | | | | | | | |
| **Campbell**^42^ | RCT | 2,519 adults  USA | Church based: Multicomponent church based intervention including help with growing fruit and vegetables, educational sessions, cookbook and recipe tasting and increasing the presence of fruit and veg offered at church functions | 5 | Mean change in portions of fruit and vegetables consumed | Household income | Low = 0.84 p = 0.001  High = 0.60, p = 0.014 | **↔*** |
|  |  |  |  |  |  |  |  |  |
| **Hughes**^45^ | Cross sectional survey | 2,306 school children  England | School based: Used a cross section of children to assess the school fruit and vegetable scheme where portions of fruit or vegetables were given to each child. | 4 | Change in portions of fruit and vegetables consumed | Index of Multiple Deprivation | Low = +0.8  High = +0.7 | **↓*** |
| **Rush**^47^ | RCT | 1,352 school children  New Zealand | School based: Multicomponent health promotion activities in Primary schools, including weekly newsletters, a home–school link programme that provided opportunities for parents to attend three information-based sessions, gala open days and edible gardens | 3 | Change in BMI standard deviation score in 5-7 year olds | Household income | Low = 0.07 (CI = -0.08, 0.21), p = 0.35  High = -0.03 (CI = -0.1, 0.04), p = 0.46 | **↔*** |
| **Sorenson**^43^ | RCT | 974 employees  USA | Work based: Worksite based multicomponent intervention including monthly Ran health and increasing the availability of healthy foods offered at meetings and events | 5 | % change in those achieving 5 a day | Occupation | Low = +7.5  High = -5.5  p = 0.048 for difference | **↓*** |
| **Sorenson**^44^ | RCT | 2,386 employees USA | Work based: Worksite multicomponent interventions including increasing the availability of healthy foods offered on the sites and health education to the employees | 5 | Change in geometric mean grams of fibre per 1000 kcals | Occupation | Low = +12  High = +6  p = 0.012 for difference | **↓*** |
| **Wendel-Vos**^46^ | Cohort study | 3,114 adults Holland | Area based: Cohort used to investigate the net effect of a total of 790 health interventions comprising the Hartslag Limburg project | 4 | Difference in mean energy intake between intervention and control (MJ/d) | Education level | (standard error)  Low = -0.2 (0.12), p = <0.05  High = -0.2 (0.1), p = >0.05 | **↓*** |
| ***Product*** | | | | | | | | |
| **Millet**^48^ | Observational study | 22,264 people England | Reformulation: Assessed the impact of the national strategy in the UK to reduce salt intake (including salt reformulation and increasing public awareness) using national survey data from the health survey for England from 2003-2007 | 3 | Salt intake (g/d) | Social class | Low = -0.74  High = -0.72 | **↔*** |
| ***Prescriptive*** | | | | | | | | |
| *No studies were identified examining the potential SEP differentials effects of restrictions on advertising/marketing through controls or bans; labelling, recommendations or guidelines* | | | | | | | | |
| ***Promotion*** | | | | | | | | |
| **Cappacci**^49^ | Modelling study | 20,722 people  UK | Health information campaign: Estimated the impact of the UK “Five a day” campaign | 2 | Change in fruit and vegetable intake (portions) | Household income | Low = +0.51, p = <0.05  High = +0.37, p = >0.05 | **↓*** |
| **Dallongeville**^34^ | Modelling study | 2,624 adults and 1,455 children France | Health information campaign: Modelled a pessimistic scenario examining the impact of a €10M information campaign promoting fruit and vegetable consumption in France | 2 | Change in fruit and vegetable consumption (g/d) | Household income | Low = +0.3 (CI = -0.1-0.5)  Rest = +0.4 (CI = 0.2-0.7) | **↔*** |
| **Estaquio**^50^ | Cohort study | 4282 adults  France | Health information campaign: Retrospective cohort examining the adherence to the French “five a day” campaign | 2 | % of males consuming ≥ five portions of fruit and vegetable per day | Education level | Low = 50.4 (CI = 0.54-0.92)  High = 58.1 1 (referent) | **↑*** |
| **Stables**^51^ | Cross sectional survey | 5,297 adults  USA | Health information campaign: Two telephone surveys in 1991 and 1997 to assess the “Five a day” campaign in the USA | 2 | Change in portions of fruit and vegetables consumed | Poverty Index Ratio | Low = +0.29, p = 0.122  High = 0, p = 0.977 | **↔*** |
| ***Person*** | | | | | | | | |
| **Brownson**^61^ | Cross sectional survey | 2,516 adults  USA | Health education: Community level multicomponent interventions including health festivals and education campaigns | 3 | % change of the % of people who consume five portions of fruit and vegetables per day | Education level | Low = +4.6  High = +0.3 | **↓** |
| **Burgi**^52^ | RCT | 652 preschool children Switzerland | Health education: Children completed 22 lessons in healthy nutrition. Health foods were promoted for snacks and treats with teacher and parent training | 5 | Mean BMI (kg/m²) | Parental education level | Low = diff = 0, p = 0.668  High =intervention had 0.3 lower than control, p = 0.253 | **↔*** |
| **Carcaise-Edinboro**^62^ | RCT | 623 adults  USA | Health education: Tailored feedback and self-help dietary intervention. Individualised intervention booklets posted to the homes of participants once a week for four weeks detailing advice on healthful eating practices | 5 | Mean fruit and veg intake score (Score out of 3, 3 = less F/V intake, 1 = more F/V intake) | Education level | Low = 1.74, p = 0.02  High = 1.83, p = 0.68 | **↓*** |
| **Connett**^63^ | RCT | 12,866 adults  USA | Dietary counselling intervention: Randomised, primary prevention trial involving dietary counselling to maintain a fat-modified diet | 3 | Change in serum cholesterol (mg/dl) | Household income | Low = -6  High = -7.3 | **↑** |
| **Curtis**^53^ | Randomised parallel groups comparison study | 169 families (589 individuals)  UK | Health education: Cooking fair with cooking lessons accompanying personalised dietary goal settings | 3 | % change in mean food energy from fat | Quintile of Deprivation Index | Low = -2.3  High = -0.4 | **↓*** |
| **Friel**^54^ | RCT | 821 school children Republic of Ireland | Health education: Piloted a ten week intervention of a pre-existing American intervention called “hearty heart” which is a children’s fictional character. This was used in intervention schools receiving 20x30 minute sessions using a cross-curriculum approach | 2 | Change in % of children consuming >4 portions of fruit and veg per day | Area level deprivation | Low = +1  High = 0 | **↔*** |
| **Haerens**^55^ | RCT | 304 school children Belgium | Health education: The intervention was an adaptation of an adult computer tailored dietary intervention. Students completed a 50 min CD-ROM alone which covered detailing the student’s dietary fat intake and then producing individual feedback based on their responses which could be saved | 4 | Change in mean dietary fat intake (g/d) | Education level | Low = Pre = 97.8 (SD = 38.9)  Post = 86.2 (SD = 39.3)  High = Pre = 118.4 (SD = 50.1)  Post = 102 (SD = 43.8) | **↔*** |
| **Havas**^64^ | RCT | 3,122 women  USA | Health education: Intervention consisted of several sessions including self-assessment and goal setting, identifying and overcoming potential barriers and maintenance strategies. Each session had a food demonstration. Individual letter sent to participants throughout | 5 | Change in mean daily servings consumed of fruit and vegetables | Education level | Low = 0.12 p = 0.43  High = 0.62, p = 0.003 | **↑*** |
| **Havas**^65^ | RCT | 2,066 women  USA | Dietary counselling intervention: Intervention involved individualised dietary counselling with a kick off fair (including taste tests, four 45 minute, behaviour-reinforcing incentives and phone calls | 5 | % change in fruit and vegetables consumed | Education level | Low = 0.13, p = 0.22  High = 0.2, p = 0.04 | **↑*** |
| **Holme**^56^ | RCT | 1,232 men  Norway | Dietary counselling intervention: Intervention involved dietary counselling and education with a clinical examination twice a year | 5 | % change in cholesterol | Social class | Low = -29  High = -46 | **↑** |
| **Jeffery**^66^ | RCT | 1,226 adults  USA | Health education through monthly newsletters in suburban communities in the US | 3 | Mean weight change in women (lb) | Household income | Low = +2.11 (SE = 0.99)  High = +1.03 (SE = 0.66) | **↔*** |
|  |  |  | Health education through monthly newsletters in suburban communities in the US with an additional financial incentive of a lottery with the chance of winning $100 per month |  |  |  | (Standard error)  Low = +3.23 (0.98)  High = +0.51 (0.64) | **↔*** |
| **Jouret**^57^ | RCT | 1,253 pre-school children  France | Health education: Educational intervention in kindergartens consisting of ten 20 minute sessions which aimed to promote healthy nutrition habits | 4 | Change in % of children overweight | Area level deprivation | Low = +4.7  High = +2 | **↓*** |
| **Lowe**^58^ | Cohort study | 402 school children  UK | Health education: The children were provided with free portions of fruit and vegetables in school and completed accompanying educational activities relating to this | 3 | % change in vegetables observed consumed | Free school meal entitlement | Low = -4  High = +4 | **↑** |
| **Plachta-Danielzik**^59^ | RCT | 1,764 school children  Germany | Health education: Lessons included healthy nutrition done as interactive games, fairytale story-telling and a practical teaching how to prepare a healthy breakfast | 5 | Change in % prevalence of overweight | Parental education level | Low = +6.5  High = -0.7 | **↑*** |
| **Reynolds**^67^ | RCT | 1,698 school children  USA | Health education: Classroom based health education with taste testing sessions of different fruits and vegetables | 3 | Portions of fruit and vegetables consumed | Household income | Difference between intervention and control  Low = +0.9  High = +1 | **↑*** |
| **Smith**^58^ | RCT | 487 adults  Australia | Health education: Participants examined their food profiles and based on this were asked to set no more than 5 dietary goals. Educational materials were posted out at monthly intervals for the participants to record and monitor these goals | 4 | Change in fat density consumed (g/4200 kcal) | The Daniel Scale of Occupational Prestige | Low = -1.75  High = -0.58 | **↓** |
| **Toft**^60^ | RCT | 11,283 adults Denmark | Dietary counselling intervention: Repeated individualised dietary counselling with the integration of family members of participants for reinforcement | 4 | Change in amount of fruit eaten by men (g/week) | Education level | Low = +218  High = +250 | **↑*** |

*^∆^Quality of empirical studies were assessed using a validated tool ^26^. Studies were scored against six criteria and this number was summed to give an overall quality score (maximum of six). The modelling studies were assessed for quality by two independent experts and their scores were converted into a score out of six to allow comparison.*

*# Quantitative change by SEP is presented with confidence intervals (95%CI), standard deviation (SD), standard error (SE) and/ or p values where available.*

†*the effect on inequalities is displayed symbolically in the table as: ↓ for an Intervention likely to reduce inequalities: the intervention preferentially improved healthy eating outcomes in people of lower SEP, ↑ for an intervention likely to widen inequalities: the intervention preferentially improved healthy eating outcomes in people of higher SEP, and ↔ for an intervention which had no preferential impact by SEP*

** indicates interventions where statistical significance values were given to the quantitative evidence relevant to our review.*
